# Focus on Chemistry of the 10-Dioxane-*nido*-7,8-dicarba-undecahydrido Undecaborate Zwitterion; Exceptionally Easy Abstraction of Hydrogen Bridge and Double-Action Pathways Observed in Ring Cleavage Reactions with OH^−^ as Nucleophile †

**DOI:** 10.3390/molecules25040814

**Published:** 2020-02-13

**Authors:** Mário Bakardjiev, Suzan El Anwar, Dmytro Bavol, Zdeňka Růžičková, Bohumír Grűner

**Affiliations:** 1 Institute of Inorganic Chemistry of Czech Academy of Sciences, 25068 Řež, Czech Republic; mario@iic.cas.cz (M.B.); elanwar@iic.cas.cz (S.E.A.); bavol@iic.cas.cz (D.B.); 2 Department of General and Inorganic Chemistry, Faculty of Chemical Technology, University of Pardubice, Studentská 95, 53210 Pardubice, Czech Republic; Zdenka.Ruzickova@upce.cz

**Keywords:** borane, carborane, dicarbollide ion, oxonium atom, nucleophilic substitution

## Abstract

Ring cleavage of cyclic ether substituents attached to a boron cage via an oxonium oxygen atom are amongst the most versatile methods for conjoining boron *closo*-cages with organic functional groups. Here we focus on much less tackled chemistry of the 11-vertex zwitterionic compound [10-(*O*-(CH_2_-CH_2_)_2_O)-*nido*-7,8-C_2_B_9_H_11_] (**1**), which is the only known representative of cyclic ether substitution at *nido*-cages, and explore the scope for the use of this zwitterion **1** in reactions with various types of nucleophiles including bifunctional ones. Most of the nitrogen, oxygen, halogen, and sulphur nucleophiles studied react via nucleophilic substitution at the C1 atom of the dioxane ring, followed by its cleavage that produces six atom chain between the cage and the respective organic moiety. We also report the differences in reactivity of this *nido*-cage system with the simplest oxygen nucleophile, i.e., OH^−^. With compound **1**, reaction proceeds in two possible directions, either via typical ring cleavage, or by replacement of the whole dioxane ring with -OH at higher temperatures. Furthermore, an easy deprotonation of the hydrogen bridge in **1** was observed that proceeds even in diluted aqueous KOH. We believe this knowledge can be further applied in the design of functional molecules, materials, and drugs.

## 1. Introduction

Eleven vertex 7,8-Dicarba-nido-dodecahydroundecaborate(1−) ion [1] belongs to the most studied boron cluster anions due to its easy availability from *ortho*-carborane [2], open pentagonal C_2_B_3_ plane with three stereochemically distinctive sites for substitution and an extra hydrogen atom sitting on it [3,4]. It is well recognized that the coupling of the [*nido-*7,8-C_2_B_9_H_12_]^−^ ion with tetrahydrofuran and dioxane promoted by various metal halides, such as FeCl_3_ [5] or HgCl_2_ [6,7], yields the neutral zwitterionic compounds [10-(CH_2_)_4_O)-*nido*-7,8-C_2_B_9_H_11_] or [10-(*O*-(CH_2_-CH_2_)_2_O)- *nido*-7,8-C_2_B_9_H_11_] (**1**). More recently, we reported that the dioxane derivative **1** can be produced in high yield by reaction of the neutral carborane *nido*-C_2_B_9_H_13_ with dioxane used as solvent [8]. This is a metal free, straightforward, and clean reaction in which the dioxane ring is attached to the *nido*-cluster by an oxonium atom to the open-face boron site B(10) that is position located opposite the cluster carbon atoms.

This compound belongs to a large and rapidly growing family of species that contain an ether ring attached to the boron cage via an oxonium atom [9,10,11,12]. All other members are derived from *closo*-borate ions such as cobalt bis(dicarbollide) [13,14], decaborate [15,16], dodecaborate [17,18,19], or 1-carba-undecahydroundecaborate [20]; most of which have been used in numerous emerging applications, among others, advanced materials [21,22], organic conducting polymers [23,24], radionuclide partitioning [25,26], biology, and medicine [8,27,28,29,30,31,32,33,34,35,36,37]. Surprisingly enough, the *nido*- system from this family of compounds, namely compound **1**, has largely remained outside the stream of main interest, despite its potential for the introduction of open-cage boron polyhedra to functional molecules and materials. This is even more surprising in the light of recent advances in the use of [7,8-C_2_B_9_H_12_]^–^ as a pharmacophore in drug design [30,38,39,40]. In this regard, the ring cleavage methodology brings a level of high versatility to a broad scope of applications. Furthermore, the resulting substituents are located on the cage in a symmetrically located position and hence the products are free from stereochemical complications such as the presence of diastereoisomeric pairs or chirality [7]. The compound, and its derivatives arising from ring cleavage, can potentially serve as new charge-compensated [41,42] or common type of ligands in the synthesis of metallacarboranes.

With regards to ring cleavage of the zwitterion **1** using nitrogen nucleophiles, only reactions with the azide ion [7] and butyl amine have been reported, the later within the synthesis of model inhibitors of HIV-Protease that bear two dicarbollide ions units interconnected with long aliphatic chains to a central n-alkylammonium group [8,36]. Compounds of this type are sometimes referred to as “dumbbells” [43]. Similar double cluster compounds resulted also from reactions of **1** with dihydroxy benzenes as oxygen nucleophile [44]. These compounds were shown to adopt crown-ether like arrangements. The reactions with other nucleophiles have been reported only for isomers of hydroxybenzoic acid [7], which lead to anionic products with one boron cage per molecule and thiourea [45].

We have been involved in the design and exploration of building blocks based on boron polyhedra, which enable the facile merging of boron cages with various organic molecules or biomolecules, particularly those applicable in drug design [40,46,47,48]. Herein we present an overview of the reactions of **1** with a variety of possible nucleophiles. Some of these reactions resulted in unexpected findings such as the easy deprotonation of the starting zwitterion even in aqueous solutions, or the detachment and substitution of the whole dioxane ring at higher temperatures. The conditions for ring cleavage have thus to be carefully considered and optimized in order for this method be used successfully for functionalization, and it is these considerations that are described herein.

## 2. Results and Discussion

### 2.1. Reaction Pathways of 1 with Nucleophiles

**Scheme 1:**
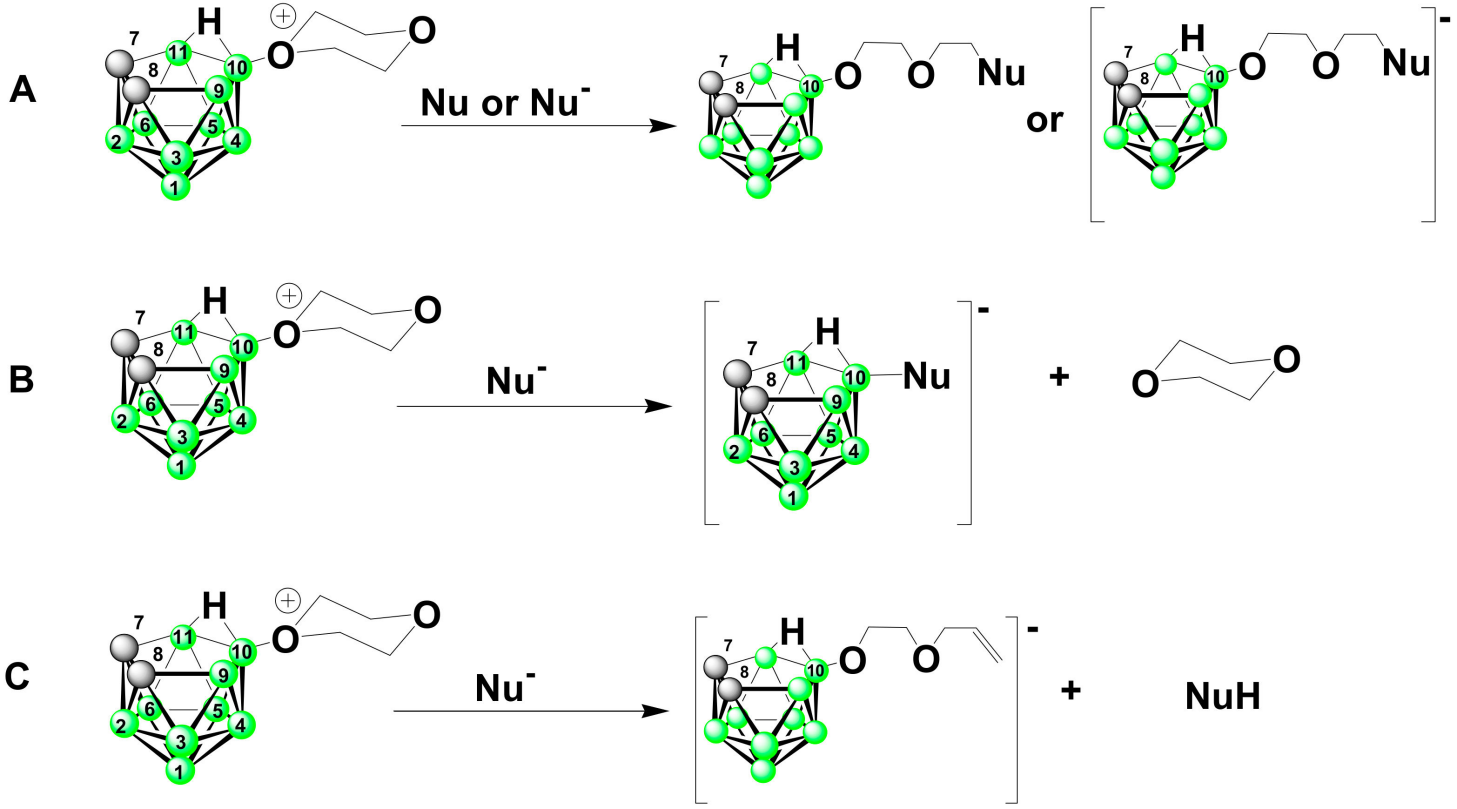
Possible reaction pathways that may apply to the reactions of the compounds containing cyclic ethers with oxonium atoms with nucleophiles, according to Ref. [49]. (**A**) Nucleophilic substitution on the ether ring at the α-carbon adjacent to the oxonium atom. This leads to the cleavage of the ring and an attachment of the nucleophile at terminal carbon atom of the diethyleneglycol chain. (**B**) Substitution of the cyclic ether by the anionic nucleophile can lead to Nu^-^ substituted boron cluster and to the elimination of the cyclic ether. (**C**) Hofmann-type dealkylation at the oxonium atom, when the anionic nucleophile Nu^–^ acts as a base abstracting the α-proton of the ring. This results in formation of a protonated nucleophile HNu and formation of a terminal double bond in the aliphatic chain.

According to the literature considerations [49], three different reaction pathways (A, B, and C below) can apply when ether rings attached to boron anions via oxonium oxygen atom, such as dioxane in compound **1** (or other tetrahydrofurane, tetrahydropyrane, etc. derivatives) are attacked with various nucleophiles, as schematically shown for the current system in Scheme 1.

To our knowledge, only the first pathway A was reported, taking into account all cyclic ether derivatives of boron clusters [9,11]. Even with anionic nucleophiles such as O^−^, Cl^−^, Br^−^, I^−^, N_3_^−^, and R-O^−^, all reactions reported in the literature led to nucleophilic substitution producing opened ether ring with nucleophile moiety attached at the carbon originally present in α-position to oxonium oxygen.

**Figure 1:**
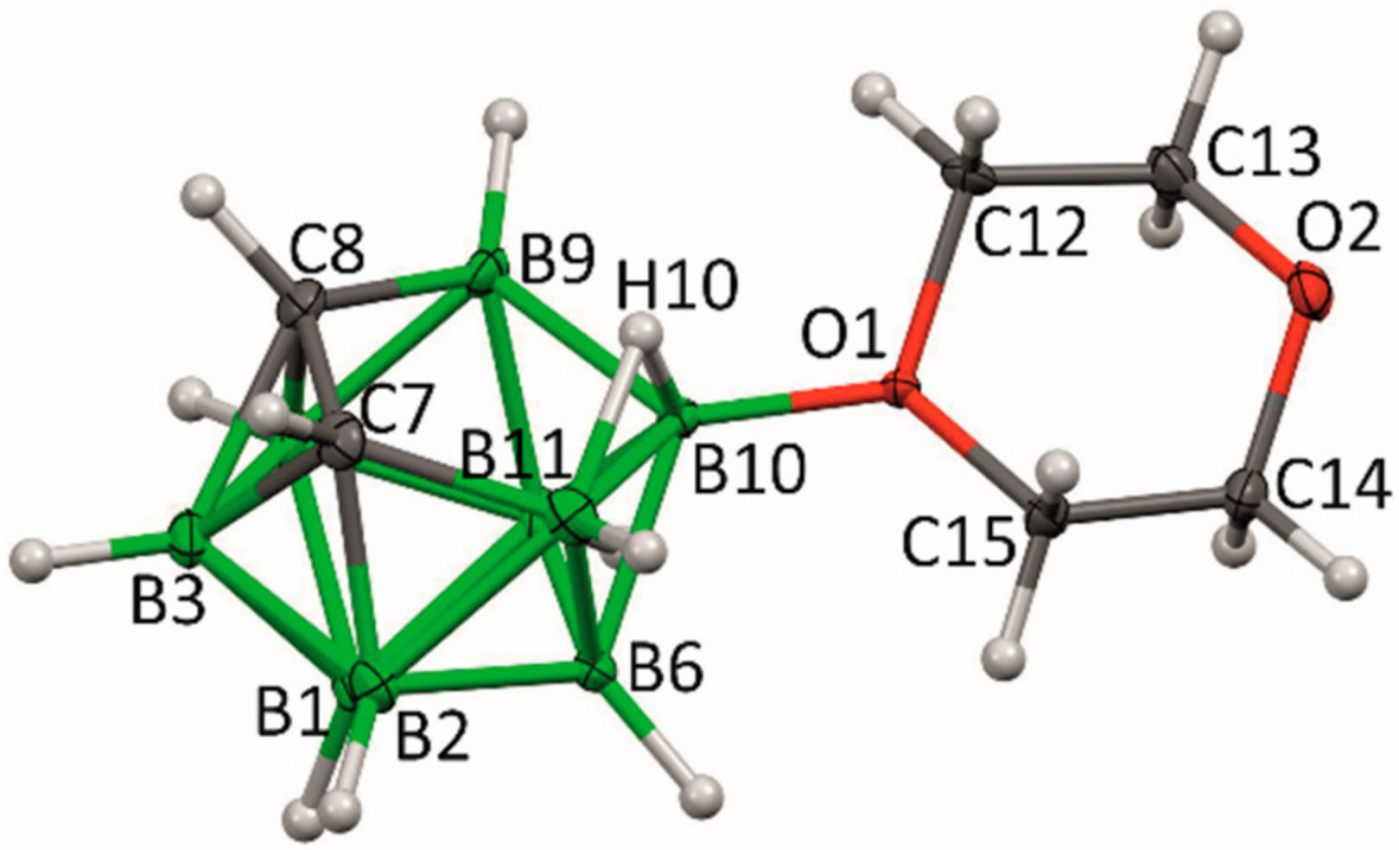
The molecular structure of **1**, ORTEP diagram, 30% probability level. Selected interatomic distances [Å] and angles [°]: O1 C12 1.484(2), O1 C15 1.490(2), O1 B10 1.518(3), B10 B6 1.771(3), B10 B5 1.771(3), B10 B9 1.778(3), B10 B11 1.855(4), O2 C13 1.419(3), O2 C14 1.420(3), B11 C7 1.623(4), B11 B6 1.798(4), B11 B2 1.811(4), C7 C8 1.552(3), C7 B2 1.705(4), C7 B3 1.718(4), B9 C8 1.606(3), B9 B5 1.767(4), B9 B4 1.784(4), C8 B4 1.725(4), C8 B3 1.731(3), B6 B2 1.763(4), B6 B1 1.794(4), B6 B5 1.811(4), B3 B2 1.760(4), B3 B4 1.763(4), B3 B1 1.774(4), B2 B1 1.753(4), C12 O1 C15 110.86(15), C12 O1 B10 115.64(15), C15 O1 B10 118.60(15), O1 B10 B6 116.41(16), O1 B10 B5 115.16(17), B6 B10 B5 61.48(15), O1 B10 B9 124.11(18), B6 B10 B9 107.75(17), B5 B10 B9 59.72(14), O1 B10 B11 125.94(18), B6 B10 B11 59.40(14), B5 B10 B11 107.75(17), B9 B10 B11 105.10(17).

**Scheme 2:**
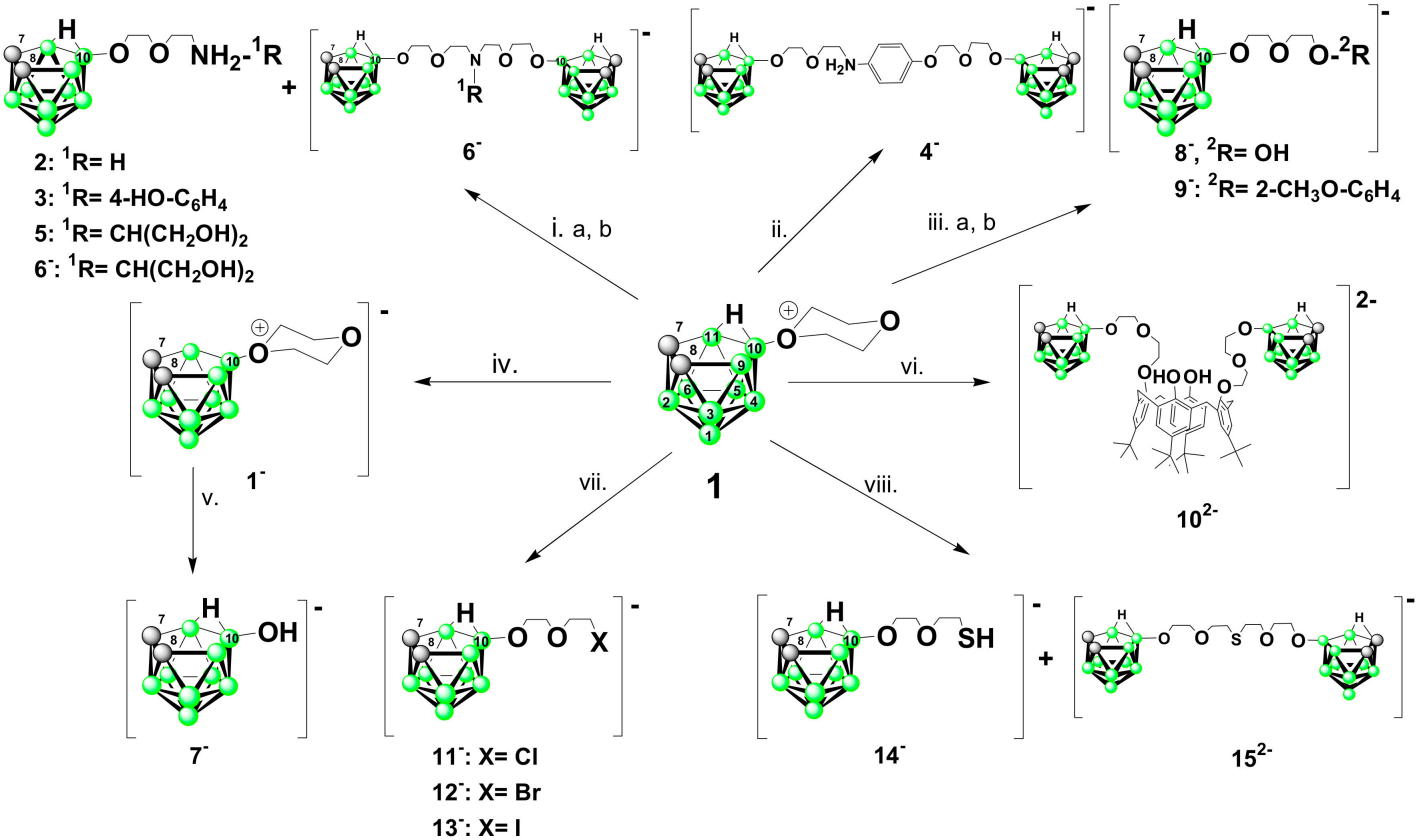
Reaction pathways observed in this study for cleavage of the ring in 1 by various nucleophiles; i. Amine in an excess, (a) THF for **2**, ACE^®^ pressure flask, (b) for **3,** THF r.t.; ii. 1-HO-C_6_H_4_-4-NH_2,_ K_2_CO_3_ in CH_3_CN, 80 °C; iii. (a) for **8^−^:** KOH water-ether, r.t. (b) for **9^−^**: K_2_CO_3_, 1-HO-2-CH_3_O-C_6_H_4_ reflux; iv. 2.5 M KOH r.t.; v. 2.5 M KOH, reflux, vi. K_2_CO_3_, CH_3_CN, t-Bu-calix[4]arene 0.5 equiv.; vii. Bu_4_NX, 2 equiv. ether, r.t.; viii. NaSH.H_2_O in excess, THF, r.t.

The ring cleavage of dioxane ring in 10-(*O*-(CH_2_-CH_2_)_2_O)-*nido*-7,8-C_2_B_9_H_11_ with nitrogen nucleophiles proceeds (Scheme 2) in a straightforward manner according to pathway A, i.e., via nucleophilic substitution that produce compounds with ammonio functions attached to the cage via a dietlyleneglycol chain [7,8]. We illustrate here reactions with amines only in three examples that highlight affinity trends observed between various types of nucleophiles (see Scheme 2). The synthesis of the simplest derivative that contains terminal –NH_3_ group is usually less straightforward than for primary and secondary amines. Thus, for other cages, protection-deprotection schemes have been sometimes reported, typically using potassium phtalimide (see e.g., Ref. [50]). Nevertheless, the amine derivative [10-(H_3_N-(CH_2_-CH_2_O)_2_-*nido-*7,8-C_2_B_9_H_11_] (**2**) can be easily prepared by reaction of **1** with NH_3_ in THF as is shown here and its molecular structure is depicted in Figure 1. (see the crystallographic discussion below; crystallographic data are given in the Materials Section and Supplementary Information).

To test further the differences between reactivity of different types of nucleophiles in this system, a single molecule, 4-aminophenol was used, which contains a weakly basic and nucleophilic amino group (pKa 5.48) and an acidic phenolic function (pKa 10.30) [51] that are separated by a phenyl ring. As expected, in the absence of a base, the ring cleavage proceeds at room temperature exclusively and quantitatively with the nitrogen end of the molecule binding via an ammonio group to the cluster to give [10-HO-C_6_H_4_-H_2_N-(CH_2_-CH_2_O)_2_-*nido-*7,8-C_2_B_9_H_11_] (**3**). However, when K_2_CO_3_ is present in dry acetonitrile, the reaction at 80 °C proceeds apparently at both nitrogen and oxygen ends of the 4-aminophenol to give as a main product the double-cluster anionic species [*nido-*7,8-C_2_B_9_H_11_-10-(CH_2_-CH_2_O)_2_-H_2_N-C_6_H_4_-O-10′-(CH_2_-CH_2_O)_2_-*nido-*7,8-C_2_B_9_H_11_]^−^ (**4^−^**). The mode of bonding could be distinguished from salt isolated directly from the reaction in the form with 4-ammonium phenol cation. ^1^H and ^13^C-NMR spectra of **4^-^** then show inequivalent signals of all eight CH_2_ groups attached to different, oxygen and nitrogen sites of aminophenol. Also aromatic signals of the central phenol unit in ^13^C-NMR are split into two sets. After metathesis to potassium salt these signals shrunk together and only 3 overlapping signals could be observed in the range of CH_2_ protons in ^1^H-NMR. Unfortunately, no crystal could be grown for XRD analysis that precluded unequivocal confirmation of the structure. Only about 15% of **3** (by NMR and HPLC) could be found in the reaction mixture stirred in CH_3_CN with K_2_CO_3_ at room temperature for 72 h. Then, another dicluster compound forms in addition to **4**^−^, which structure probably corresponds to *N*,*N*-substitution at aminophenol (according to ^1^H and ^13^C-NMR spectra of a crude product), however the analytically pure compound could not be isolated from complex mixture.

Similarly, the reaction of **1** with two equivalents of 2-amino-1,3-propanediol (Serinol), a carbohydrate mimetic that contains amino function and two hydroxyl groups on an aliphatic hydrocarbon chain, proceeds without the necessity for another base, and preferentially at amino group, to give a main product that contains a terminal serinol moiety N-attached to the diethyleneglycol chain in [10-(HOCH_2_)_2_CHNH_2_-(CH_2_-CH_2_O)_2_-*nido-*7,8-C_2_B_9_H_11_] (**5**). The second product corresponds to an anion containing a secondary amine as the central moiety to which two cluster units are bound [(HOCH_2_)_2_CHNH-(10,10′-(CH_2_-CH_2_O)_2_-*nido-*7,8-C_2_B_9_H_11_)_2_]^−^ (**6**^−^).

A fundamental difference between compound **1**, which is of an open-cage *nido*-geometry, with that of the more-studied *closo*-cages, is the presence in **1** of a bridging hydrogen atom. With the aim to investigate the relevance of this hydrogen bridge to the reactivity of zwitterion **1**, the basic protonation/ deprotonation of **1** was studied with KOH as base. The sparingly water soluble starting compound dissolves only slightly upon addition of aqueous KOH at room temperature up to 2.5 M concentration. Poor solubility was responsible for weak signals observed in NMR spectrum that precluded carry out NMR titration in deuterium oxide. However, when the compound was dissolved in 100 μL of CD_3_CN and treated with 500 μL of 2.5 M KOH in D_2_O, ^11^B-NMR spectrum of freshly prepared sample showed appreciable shift of signals of boron atoms B(1 and 10), with upfield shift 11.3 ppm (see Figure S1 in Supplementary Information). Such a high shift of boron position B(1) to lower frequency is well known from the literature and could be ascribed to the absence of the extra hydrogen atom [52,53]. Also, another diagnostic peak for hydrogen bridge in ^1^H [^11^B]-NMR spectrum at approx. −0.6 ppm completely disappeared. However, this could be also ascribed to an exchange of the hydrogen atom for deuterium. Indeed, additions of 0.5 equivalents of KOH to solution of **1** in D_2_O, to which 50% of THF-d_8_ was added for complete dissolution, caused mainly by the exchange of the bridge proton for deuterium with partial disappearing of the signals for B(1) at −40.9 ppm originally present in the spectrum, when new signal at −41.2 ppm appeared. After addition of 1.5 equivalents of KOH both signals were no longer present and moved by incremental shift ca. 10 ppm to higher field to −51.7 ppm (see Figure S2 in Supplementary Information), apparently due to complete removal of the extra hydrogen atom. However, it should be noted a partial degradation and formation of side products also occurred in THF-*d*_8_ solution. Therefore, this could partly affect actual concentrations of the species **1** and **1^−^** and KOH in solution.

Such an easy and complete abstraction of the hydrogen bridge from 11-vertex cages by diluted solution of potassium hydroxide was never reported, furthermore carried out in water at room temperature. In contrast, the hydrogen bridge of the parent ion [C_2_B_9_H_12_]^−^ can be abstracted only by a strong base in anhydrous solvents and the ratio of deprotonated species in 12 M KOH has been estimated around 1% [54]. Even in the case of the similarly univalent tricarbollide ion [7,8,9-C_3_B_8_H_12_]^–^, a strong base (e.g., NaH, RLi, or Tl^+^ and/or heating) and anhydrous solvents are necessary for deprotonation [55,56,57], though the neutral ammonium derivative exhibits equilibrium between two tautomeric forms, depending on polarity of the solvent, with a proton sitting either on the cage or at ^t^BuNH_2_- group or in both positions [58,59].

**Figure 2:**
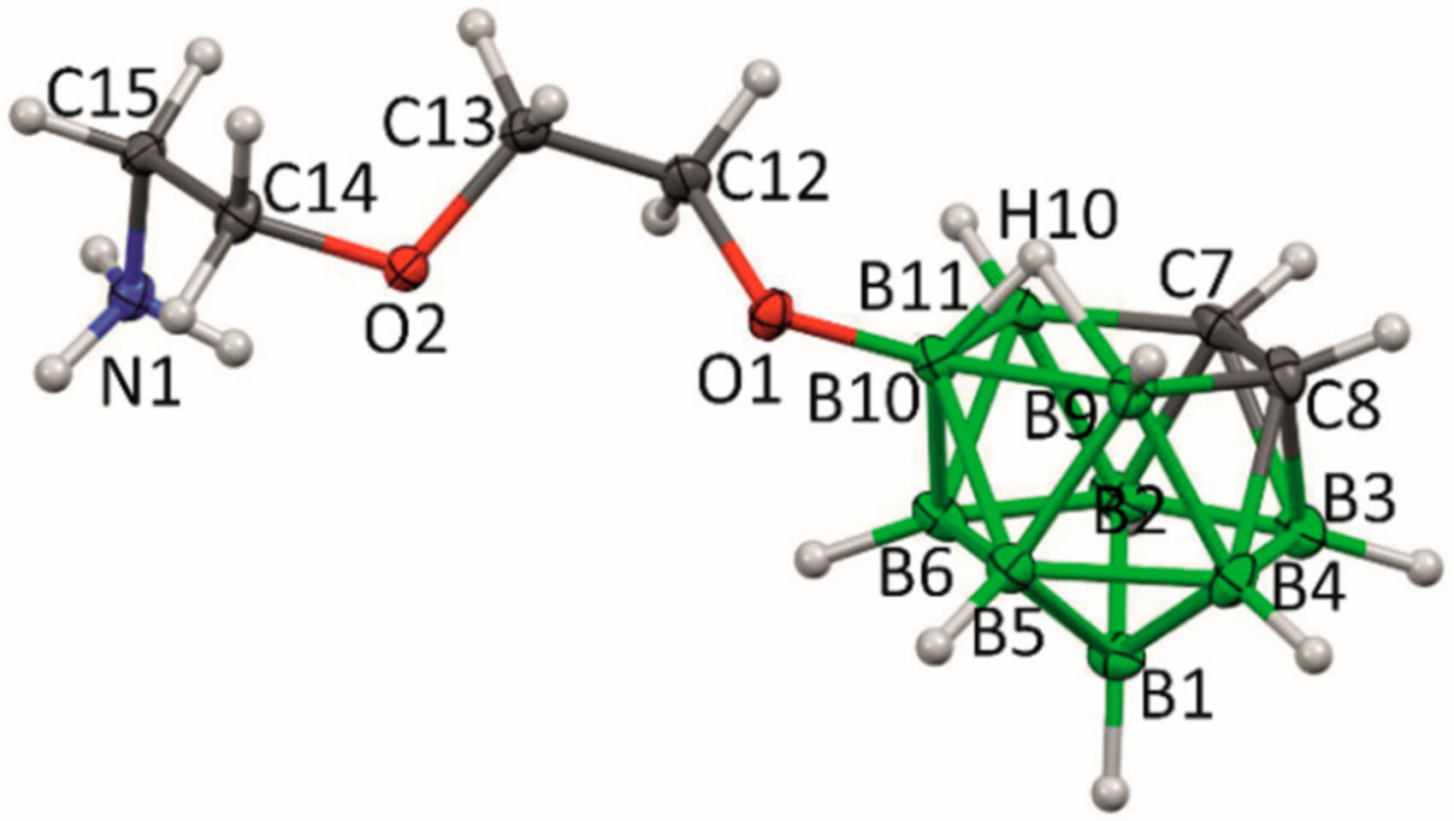
The molecular structure of **2**, ORTEP diagram, 40% probability level. Selected interatomic distances [Å] and angles [°]: O1 C12 1.4400(18), O1 B10 1.459(2), N1 C15 1.490(2), B1 B4 1.762(3), B1 B2 1.771(3), B1 B3 1.772(3), B1 B6 1.791(3), B1 B5 1.799(3), O2 C14 1.4304(18), O2 C13 1.4351(19), B2 C7 1.721(2), B2 B6 1.746(3), B2 B3 1.764(3), B2 B11 1.788(3), B3 C8 1.713(2), B3 C7 1.727(3), B3 B4 1.757(3), B4 C8 1.711(3), B9 B10 1.850(2), B10 B11 1.836(2), C12 O1 B10 113.49(12), B4 B1 B2 107.94(13), B4 B1 B3 59.62(11), B2 B1 B3 59.74(11), B4 B1 B6 108.05(13), B2 B1 B6 58.70(11), B3 B1 B6 106.19(13), B4 B1 B5 59.23(10), B2 B1 B5 107.59(13), B3 B1 B5 106.31(13), B6 B1 B5 61.06(10).

Herein we also report x-ray structure of protonated form of the zwitterionic compound **1** (see Figure 2, the crystallographic discussion below, Materials Section and Supplementary Information), unfortunately the structure of the corresponding potassium salt could not be obtained.

Further, the ring cleavage reaction by KOH was studied under several experimental conditions. Heating with 2.5 M aqueous KOH led to almost quantitative substitution of the ether by -OH to give the [10-HO-7,8-C_2_B_9_H_11_]^−^ anion (**7^−^**). This pathway corresponds to the reaction type B (Scheme 1), and is anomalous among all borate ion derivatives bearing a cyclic ether moiety. However, this mechanism we observed only for the OH^-^ ion as nucleophile and at higher temperatures. In this case, the deprotonation probably proceeds in the first step and the whole ring in [10-(O(CH_2_-CH_2_)_2_O)-*nido-*7,8-C_2_B_9_H_11_]^−^ anion is then replaced by OH^−^, according to pathway B in Scheme 1. This compound is known to form upon hydride abstraction from [C_2_B_9_H_12_]^−^ ion by AlCl_3_ in acetone [60]. Conditions described herein thus provide an alternative synthetic procedure to this symmetrically substituted 10-hydroxyderivative.

Under mild conditions, when the compound **1** is heated in a biphasic system of ether and 2.5 M KOH for 48 h, the nucleophilic addition proceeds via pathway A instead and the expected product, with a hydroxyl group attached via a diethylene glycol chain [10-HO-(CH_2_CH_2_O)_2_-7,8-C_2_B_9_H_11_]^−^ (**8^−^**) (see Scheme 2), is obtained in a good yield. However, use of KOH in other solvents such as dry dioxane, THF or acetonitrile led to quite complicated product mixtures that contained both, **7^−^** and **8^−^** as well as some other species that presumably originate from reactions with solvent and from degradation of the cage. Details are not reported here.

The ring cleavage with other oxygen nucleophiles follows the usual pathway A corresponding to SN substitution. The previously reported reactions with phenolate ions led solely to pathway A and the corresponding phenols attached to the cage by a six atom linker [7,44]. Also reactions with 2-methoxy-phenol (guaiacol) as an example of naturally occurring compound and t-Bu-calix[4]arene, as an archetype for organic platform with four phenolic -OH sites, provided the expected products (**9^−^** and **10^2−^**) according to the pathway A, when the respective compounds were reacted with K_2_CO_3_ and **1** in dry acetonitrile. A compound with a terminal guaiacol moiety [10-(2-CH_3_O-C_6_H_4_O)-(CH_2_-CH_2_O)_2_-*nido-*7,8-C_2_B_9_H_11_]^−^ (**9^−^**) was formed in high yield. This product was synthesized also with regard to easy crystallization observed previously with metal bis(dicarbollide) analogues [61,62], apparently due to five coordination sites (four oxygens and B-H site of the cage) available for alkali metal complexation. However, in this particular case these expectations proved groundless, apparently also due to different *nido*-system.

The reaction with two equivalents of **1** with t-Bu-calix[4]arene in presence of K_2_CO_3_ resulted in a doubly substituted product **10^2−^** that has two boron cages in symmetric positions 1,3- at the narrow rim of the platform that are located in positions separated by unsubstituted calix[4]arene phenol rings. The dicesium salt of the main isolated species adopts a cone-conformation at room temperature in solution as evidenced from NMR data that shows two signals for phenol ring, two AX doublets (Δδ = 1.21 ppm) for bridging -CH_2_- groups and two *^t^*Bu signals in a typical range. This could be interpreted via the analogy of a similar known derivative, in which a structure containing two cobalt bis(dicarbollide) and diethyleneglycol chains are responsible for complexation of one caesium cation above the platform. The second Cs^+^ was found incorporated inside the *^t^*Bu-calix[4]arene cavity [63]. Therefore, the presence of two caesium cations in **10^2−^** apparently also helps to fix t-Bu-calix[4]arene in cone-conformation even in hot water or acetone solutions used for crystallization and NMR measurements, respectively.

Considering other nucleophiles, halogen anions X^−^ (X = Cl^−^, Br^−^, I^−^) react with **1** readily and under mild conditions following the typical reaction pathway A, i.e., the cleavage of the ring producing the SN substitutions with halogen. Thus, stirring of the zwitterion **1** with two equivalents of Bu_4_NX (X = Cl, Br, I) in diethyl ether or THF at room temperature provides after 12 h quantitative conversion to the [10-X-(CH_2_-CH_2_O)_2_-*nido-*7,8-C_2_B_9_H_11_]Bu_4_N salts of the respective anions (**11^–^** to **13^–^**) (X = Cl, Br, I). The compounds with terminal halogens can readily serve as valuable synthons in various alkylation reactions. Despite low nucleophilic action of halide ions observed in the reactions of tetrahydropyrane derivative of [B_12_H_12_]^−^ ion [49], where the ring could not be cleaved, the reactions of **1** proceed smoothly like in the case of cobalt bis(dicarbollide) ion [64]. On the other hand, this can be a source of problems when trying to accomplish reactions with other nucleophiles and a source of halide ions, e.g., Cl^−^, is also present in a reaction mixture. Thus, according to our experience, all amines have to be carefully released from respective hydrochlorides and adequately purified before use to avoid side reactions with the chloride anion.

Another known type of strong nucleophile is represented by thiolate ion. Since thiol derivatives, may provide interactions with thiol groups in protein structure and are suitable functional group for further labelling, e.g., by fluorescent groups we focused here also on their synthesis. The ring cleavage with sodium hydrogen sulphide leads to nucleophilic substitution of type A resulting in a compound with a terminal –SH group [10-HS-(CH_2_-CH_2_O)_2_-*nido-*7,8-C_2_B_9_H_11_]^−^ (**14^−^**). This compound is identical with product that was prepared previously by two step procedure that comprised ring opening of **1** with thiourea followed by alkaline hydrolysis [45]. This derivative may be a good building block for bio-conjugation and has also potential for use in BNCT as easily available alternative of doubly charged [B_12_H_11_SH]^2−^ (BSH) [65]. Also, a double cage compound (**15^2−^**) that contains central thioether moiety [S-(10,10′-(CH_2_-CH_2_O)_2_-*nido-*7,8-C_2_B_9_H_11_)_2_]^2−^ forms as a side product of this reaction, even if an excess of sodium sulphide and a larger dilution were applied. Both compounds were isolated by chromatography and fully characterized.

### 2.2. Crystallography

The molecular structures of two compounds from the series, the starting zwitterion **1** and ammonium derivative **2** were unambiguously confirmed by sc-XRD determination. Both compounds have the eleven-vertex *nido*-type structures with the oxygen substitution at the B10 atom. Compound **1** crystallizes in the chiral orthorhombic space group, while **2** in the monoclinic *P*2_1_/n. All the structural parameters, e.g., interatomic distances and angles in both molecules are close to the appropriate values found for related 10-(2-dimethylsulfonioethoxy)-7,8-dicarba-*nido*-undecaborate [66]. Namely, the C7–C8 distances (see in captions of Figure 1 and Figure 2) are the shortest within the cluster but still a bit longer than the typical C*sp^3^*-C*sp^3^* bond distance. C-B separations are around the mean value 1.6 Å and the B-B distances of ~1.85 Å for atoms bound with the bridging H atoms are the longest in all boron clusters. The placement of the bridging hydrogen atom between B10 and B11 atoms in **1** is unequivocal as seen mainly from the distinct distances 1.778(3) Å for B10–B9 and 1.855(4) Å for B10–B11, resp. On the other hand, in **2** with similar separations of 1.850(2) Å for B9−B10 and 1.836(2) Å for B10–B11, the position is selected according the appearance of a stronger maximum on the Fourier electron density map. The major differences are also seen in the O1 bonding patterns, where in **1** the connections of this atom by three ‘covalent’ bonds are taking place, while ~ 0.05 Å shorter connections to B10 and C12 are observed in **2**. Similar C-O and B-O distances, as found for **1**, were already reported for 1,4-dioxane derivatives of cobalt bis(dicarbollide) [14] and *closo*-dodecaborate [10], with the exception of the B-O distance in the latter compound, which is elongated by 0.03 Å. The direct comparison of **1** with recently published 10-Et_2_O-7,8-C_2_B_9_H_11_ derivative [67] shows essentially the same interatomic distances and angles with only negligible differences in the structural parameters. However a closer look revealed the fact that the crystals of compounds **1** and 10-Et_2_O-7,8-C_2_B_9_H_11_ virtually correspond to two different enantiomers (considering the asymmetric position of extra hydrogen atoms).

In addition, the supramolecular architecture differs a lot. The molecules in the crystals of **1** are connected only by non-covalent C-H…H-B and C-H…O interactions, in the crystals of **2**, also classical H-bridging between N-H and three oxygen atoms of the OCH_2_CH_2_O groups are detected.

## 3. Materials and Methods

### 3.1. General

The starting trimethylammonium salt of dicarbollide anion was purchased from Katchem Ltd. Prague, Czech Republic. The starting compound **1** was prepared according to described procedure [8]. 1,4-dioxane, tetrahydrofuran (THF) and ethylene glycol dimethyl ether (DME) were dried with sodium diphenyl ketyl and distilled prior to use. Acetonitrile was dried over molecular sieves 4A (Fluka, Buchs, Switzerland). Other chemicals and solvents were obtained from Aldrich, Merck Lachema a.s., Neratovice and Penta Ltd., Prague, Czech Republic and used without purification. Analytical TLC was carried out on Silufol (Lachema, Votice, Czech Republic), silica gel TLC plates in CH_2_Cl_2_-CH_3_CN mixture. Unless otherwise specified, column chromatography was performed on high purity silica gel (Merck Grade, Type 7754, 70–230 mesh, 60 Å). All reactions were performed using standard Schlenk type vacuum or inert-atmosphere techniques. Some operations, such as column chromatography and crystallization, were carried out in the air.

The identities of all the reported compounds were unambiguously verified by a combination of ^11^B, ^1^H and ^13^C-NMR spectral data (complete assignment of the resonances) Mass Spectrometry (two decimal place resolution); elemental analysis; TLC; and other methods. The purity was also assayed by a previously developed analytical HPLC method with DAD detection, under chromatographic conditions specified in the paragraph below and was ≥95% for all compounds. For chemical analyses the Me_4_N^+^ or Bu_4_N^+^salts were used.

### 3.2. Instrumental Techniques

^1^H, ^1^H[^11^B], ^1^H[^11^B_selective_], ^13^C, and ^11^B-NMR spectra were measured on a Varian Mercury 400^Plus^ and a on Jeol 600 MHz spectrometers. The spectra of all compounds were measured immediately after dissolution in particular deuterated solvent, usually acetonitrile-d_3_ unless otherwise stated. ^11^B-NMR (192 MHz) chemical shifts are given in ppm to high-frequency (low field) to F_3_B OEt_2_ as the external reference. Residual solvent ^1^H resonances were used as internal secondary standards. The NMR data are presented in the text as follows: ^11^B-NMR: ^11^B chemical shifts *δ* (ppm), multiplicity. Signal assignments are based on [^11^B-^11^B] COSY NMR spectroscopy. ^1^H-NMR (600 MHz) and ^13^C (150 MHz): chemical shifts *δ* are given in ppm relative to Me_4_Si (0 ppm) as the external standard, coupling constants *J*(*H,H*) are in Hz.

Mass spectrometry measurements were performed on Thermo-Finnigan LCQ-Fleet Ion Trap instruments (San Jose, CA, USA) using electrospray ionization (ESI) for ionic species with detection of negative or positive ions. For ESI, samples dissolved in acetonitrile (concentrations approx. 100 ng mL^−1^) were introduced to the ion source by infusion. Molecular ions [M]^−^ were detected for all univalent anions as base peaks in the spectra. Full agreement between the experimental and calculated isotopic distribution pattern was observed for all isolated compounds. The isotopic distribution in the boron plot of all peaks was in complete agreement with the calculated spectral pattern. The data are presented for the most abundant mass in the boron distribution plot (100%) and for the peak on the right side of the boron plot corresponding to the *m*/*z* value.

**HPLC:** A Merck-Hitachi LaChrom Series 7000 HPLC system equipped with a DAD 7450 detector and a L7250 Programmable Autosampler was used. Anion separation was carried out according to a previously reported Ion-Pair RP method [68] for separation of hydrophobic borate anions on a RP Separon SGX C8 column, 7 μm (250 × 4 mm I. D.), Tessek Prague. The mobile phase was 4.5 mmol hexylamine acetate in 45% aqueous CH_3_CN (pH 6.0). Samples with concentration of approximately 1 mg∙mL^−1^ in the mobile phase were injected. DAD detection was carried out at fixed wavelengths 220, 225, 235, 260 nm; samples of concentration approximately 1 mg∙mL^−1^ in the mobile phase were injected.

Elemental analyses were performed on a Thermo Fisher Scientific FlashSmart Organic Elemental Analyzer (Austin, TX, USA) using a V_2_O_5_ catalyst weighted with the sample for combustion of the samples in oxygen. Most of compounds for EA were measured as K^+^ or Na^+^ or were converted to respective Me_4_N^+^ or Bu_4_N^+^ salts after careful drying in vacuum. The alkali metal and Me_4_N^+^ salts of compounds **6^−^** and **8^−^** tend to strongly bind water and sodium from the chromatographic support, thus for elemental analysis were converted to respective PPh_4_^+^ salts by precipitation with PPh_4_Cl. All compounds were dried for 12 h in vacuum at 80 °C before analyses.

**X-ray crystallography:** The X-ray data for the compound **1** and **2** (colorless crystals by slow evaporation of a methanol/water solution) was collected at 150(2)K with a Bruker D8-Venture diffractometer equipped with Mo (Mo/K_α_ radiation; λ = 0.71073 Å) microfocus X-ray (IµS) source, Photon CMOS detector (Karlsruhe, Germany) and Oxford Cryosystems cooling device (Oxford, United Kingdom) was used for data collection. The frames for both **1** and **2** were integrated with the Bruker SAINT software package (Karlsruhe, Germany) using a narrow-frame algorithm. Data were corrected for absorption effects using the Multi-Scan method (SADABS). The ratios of minimum to maximum apparent transmission were 0.892 and 0.921. The structures were solved and refined using XT-version 2014/5 and SHELXL-2017/1 software implemented in APEX3 v2016.9-0 (Bruker AXS, Karlsruhe, Germany) system [69]. *R*_int_ = ∑|*F*_o_^2^ − *F*_o,mean_^2^|/∑*F*_o_^2^, S = [∑(*w*(*F*_o_^2^ − *F*_c_^2^)^2^)/(*N*_diffrs_ − *N*_params_)]^½^ for all data, *R*(*F*) = ∑||*F*_o_| − |*F*_c_||/∑|*F*_o_| for observed data, *wR*(*F*^2^) = [∑(*w*(*F*_o_^2^ − *F*_c_^2^)^2^)/(∑*w*(*F*_o_^2^)^2^)]^½^ for all data. Hydrogen atoms were mostly localized on a difference Fourier map; however, to ensure uniformity of treatment of crystal, all hydrogen were recalculated into idealized positions (riding model) and assigned temperature factors H_iso_(H) = 1.2 U_eq_ (pivot atom) or of 1.5 U_eq_ (methyl). H atoms in methylene moieties were placed with C-H distances of 0.96 Å, 0.82Å for N-H and 1.1 Å for B-H and C-H bonds in the carborane cages. For crystallographic data and structure refinement see ESI. Crystallographic data for structural analysis have been deposited with the Cambridge Crystallographic Data Centre, CCDC nos. 1973450-1973451. Copies of this information may be obtained free of charge from The Director, CCDC, 12 Union Road, Cambridge CB2 1EY, UK (fax: +44-1223-336033; e-mail: deposit@ccdc.cam.ac.uk or www: http://www.ccdc.cam.ac.uk).

### 3.3. Synthetic Procedures

Deprotonation of the [10-(*O*-(CH_2_-CH_2_)_2_O)-*nido*-7,8-C_2_B_9_H_11_] (**1**)

400 μL of 2.5 M KOH in D_2_O was added to a 100 μL of 1.25 M solution of **1** in CD_3_CN in a 5 mm NMR tube. The insoluble zwitterion dissolved during 10 min. under shaking and the respective anion formed, from which the bridge was fully abstracted as evidenced by significant changes in ^11^B and ^1^H-NMR. The sample was measured immediately after dissolution. After several hours the salt starts to crystallize out of the solution. After acidification with few drops of 3 M HCl this ion reverts back to neutral zwitterion.

NMR data for the starting zwitterion [10-(*O*-(CH_2_-CH_2_)_2_O)-*nido*-7,8-C_2_B_9_H_11_] (**1**) ^11^B-NMR (192.6 MHz, CD_3_CN) δ (ppm) = −10.6 (s, 1B, B10), −13.3 (d, 2B, *J* = 146 Hz, B9,11), −18.0 (d, 2B, *J* = 137 Hz, B5,6), −23.2 (d, 3B, *J* = 151 Hz, B2,4,3), −40.8 (d, 1B, *J* = 146 Hz, B1); ^1^H-NMR (600 MHz, CD_3_CN): δ (ppm) = 4.48 (t, 4 H, *J* = 4.2 Hz, CH_2_O), 3.83 (t, 4 H, *J* = 4.8 Hz, CH_2_O), 1.99 (br. s, 2 H, CH_carborane_); ^13^C-NMR (100.6 MHz, CD_3_CN) δ (ppm) = 82.3 (CH_2_O), 64.8 (CH_2_O), 41.3 (CH_carborane_).

NMR data for the deprotonated [10-(*O*-(CH_2_-CH_2_)_2_O)-*nido*-7,8-C_2_B_9_H_10_]^−^ (**1**^−^) ^11^B-NMR (192.6 MHz, D_2_O) δ (ppm) = −22.5 (d, 2B, *J* = 120 Hz, B9,11), −24.8 (d, 2B, *J* = 111 Hz, B5,6), −30.0 (2d + s, 4B, *J* = 129 Hz, B2,4,3,10), −52.1 (d, 1B, *J* = 128 Hz, B1); ^1^H-NMR (600 MHz, D_2_O: δ (ppm) = 4.12 (t, 4 H, *J* = 4.2 Hz, CH_2_O), 3.73 (t, 4 H, *J* = 4.8 Hz, CH_2_O), 0.65 (br. s, 2H, CH_carborane_); ^13^C-NMR (100.6 MHz, CD_3_CN) δ (ppm) = 79.6 (CH_2_O), 65.6 (CH_2_O), 24.0 (CH_carborane_).

[10-H_3_N-(CH_2_-CH_2_O)_2_-*nido*-7,8-C_2_B_9_H_11_] (**2**)

The zwitterion **1** (205 mg, 0.927 mmol) was dissolved in THF (15 mL) in a glass ACE^®^ pressure tube, which was cooled down to −78 °C under nitrogen and then ammonia (3.0 g, 0.176 mol) was condensed into this solution. The tube was closed by a stopper and heated up. The reaction mixture was stirred at room temperature for 1 h 30 min, and then the solvent was removed on a rotary evaporator. MS of the reaction mixture showed the mass of the expected product and, in addition also a peak of the respective double-cluster compound. This however was not isolated in analytical purity. The pure compound **2** was isolated by chromatography on silica gel column 15 × 1.5 cm I.D. elution with CH_2_Cl_2_-CH_3_CN mixture from 5:1 to 1:1 b.v. The respective fractions were evaporated and the product was dried in vacuum for 3 h; white solid, yield 0.103 g, 47%. ^11^B-NMR (192.6 MHz, CD_3_CN) δ (ppm) = −11.4 (s, 1B, B10), −13.5 (d, 2B, *J* = 138 Hz, B9,11), −18.3 (d, 2B, *J* = 133 Hz, B5,6), −24.6 (d, 2B, *J* = 151 Hz, B2,4), −25.8 (d, 1B, B3, *J* = 137 Hz), −41.5 (d, 1B, *J* = 137 Hz, B1); ^1^H-NMR (600 MHz, CD_3_CN): δ (ppm) = 6.82 (br. s, 2H, H_2_N), 3.64 (m, 4H), 3.55 (m, 2H), 3.05 (t, 2H), 1.63 (br. s, 2 H, CH_carborane_); ^1^H[^11^B]-NMR (400 MHz, CD_3_CN): δ (ppm) = 6.82 (br. s, 3H, H_3_N), 3.64 (m, 4H), 3.55 (m, 2H), 3.05 (t, 2H, *J* = 5 Hz), 2.06 (br. s, 2 H, BH), 1.94 (br. s, 1 H, BH), 1.63 (br. s, 2 H, CH_carborane_), 1.38 (br. s, 2 H, BH), 1.24 (br. s, 1 H, BH), 1.09 (br. s, 1 H, BH), −0.60 (br. s, 1 H, μ-H); ^13^C-NMR (100.6 MHz, CD_3_CN) δ (ppm) = 71.0 (CH_2_N), 70.0 (CH_2_O), 66.1 (CH_2_O), 39.7 (CH_2_O), 39.4 (CH_carborane_); MS (ESI^−^) *m*/*z* (%) found: 237.40 (100), 238.36 (50) [M − H]^−^, calcd. 237.25 (100), 238.24 (48); Analysis found (%): C 30.12, H 9.04, N 5.86; calcd. for C6B9H21O2N C 30.47, H 8.95, N 5.92.

[10-HO-C_6_H_4_-H_2_N-(CH_2_-CH_2_O)_2_-*nido*-7,8-C_2_B_9_H_11_] (**3**)

The zwitterion **1** (300 mg, 1.36 mmol) was dissolved in THF (25 mL) and then solid 4-aminophenol (190 mg, 1.74 mmol) was added under stirring. The reaction mixture was stirred at room temperature for 24 h until the spot of starting compound disappeared on the TLC, and then the solvent was removed on a rotary evaporator. The product was crystallized from CH_2_Cl_2_-hexane, filtered and dried in vacuum; yellowish solid, yield 416 mg, 93%: ^11^B-NMR (192.6 MHz, CD_3_CN) δ (ppm) = −11.3 (s, 1B, B10), −13.5 (d, 2B, *J* = 133 Hz, B9,11), −18.3 (d, 2B, *J* = 131 Hz, B5,6), −24.6 (d, 2B, *J* = 153 Hz, B2,4), −25.7 (d, 1B, B3), −41.5 (d, 1B, *J* = 142 Hz, B1); ^1^H-NMR (600 MHz, CD_3_CN): δ (ppm) = 7.16 (dd, 2 H, *J* = 9.0 Hz, ArH), 6.85 (dd, 2 H, *J* = 8.4 Hz, ArH), 4.26 (br. s, 2 H, H_2_N), 3.72 (m, 2 H, CH_2_N), 3.61 (m, 4 H, CH_2_O), 3.35 (br t, 2 H, *J* = 4.8 Hz, CH_2_O), 1.61 (br. s, 2 H, CH_carborane_); ^1^H[^11^B]-NMR (400 MHz, CD_3_CN): δ (ppm) = 7.16 (dd, 2 H, *J* = 9.0 Hz, ArH), 6.85 (dd, 2 H, *J* = 8.4 Hz, ArH), 4.26 (br. s, 2 H, H_2_N), 3.72 (m, 2 H, CH_2_N), 3.61 (m, 4 H, CH_2_O), 3.35 (br t, 2 H, *J* = 4.8 Hz, CH_2_O), 2.06 (br. s, 2 H, BH), 1.78 (br. s, 1 H, BH), 1.61 (br. s, 2 H, CH_carborane_), 1.59 (br. s, 2 H, BH), 1.24 (br. s, 1 H, BH), 1.06 (br. s, 1 H, BH), −0.58 (br. s, 1 H, μ-H); ^13^C-NMR (100.6 MHz, CD_3_CN) δ (ppm) = 156.9 (ArC), 127.9 (ArC), 123.9 (ArC), 116.3 (ArC), 70.3 (CH_2_N), 70.1 (CH_2_O), 64.1 (CH_2_O), 49.8 (CH_2_O), 39.6 (CH_carborane_); MS (ESI^−^) *m*/*z* (%) found: 329.40 (100), 330.36 (54) [M − H]^−^, calcd. 329.27 (100), 330.27 (53); Analysis found (%): C 43.79, H 7.57, N 4.40; calcd. for C12B9H25O3N 43.86, H 7.67, N 4.26.

[*Nido*-7,8-C_2_B_9_H_11_-10-(CH_2_-CH_2_O)_2_-H_2_N-C_6_H_4_-*O*-10′-(CH_2_-CH_2_O)_2_-*nido*-7,8-C_2_B_9_H_11_]K (K.**4**)

The zwitterion **1** (330 mg, 1.50 mmol) was dissolved in dry CH_3_CN (15 mL) and then solid K_2_CO_3_ (1.0 g, 7.2 mmol) was added followed by 1-HO-C_6_H_4_-4-NH_2_ (390 mg, 3.38 mmol). The reaction mixture was stirred at 80 °C for 24 h. After cooling down, the excess of K_2_CO_3_ was filtered off under nitrogen and the solvent was removed in vacuum. The crude product, which contained ca. 10% of compounds **3** was dissolved in CH_2_Cl_2_ and poured on a top of silica gel column. The pure compound **4^-^** was then isolated by chromatography on silica gel column 25 × 1.5 cm I.D. elution with CH_2_Cl_2_-CH_3_CN mixture from 5:1 to 2:1 b.v. The respective fractions were evaporated and the product was dried in vacuum for 3 h; white solid, yield of **4^−^** in the form of salt with 1-HO-C_6_H_4_-4-NH_3_^+^ cation after isolation was 181 mg, 37%. This salt was then dissolved in ethyl acetate (25 mL) shaken with diluted HCl (4 × 20 mL), then with 5% KOH (4 × 20 mL) and aqueous KCl (10%, 5 × 20 mL) and water. The organic layer was then separated, water (5 mL) was added and the volatiles were removed in vacuum. The K.**4** was then dried at 40 °C in vacuum for 6 h, yield 135 mg, 31%. [HOC_6_H_4_NH_3_]**4**: ^11^B-NMR (192.6 MHz, CD_3_CN) δ (ppm) = −10.7 (s, 1B, B10), −13.5 (d, 2B, *J* = 135 Hz, B9,11), −18.5 (d, 2B, *J* = 133 Hz, B5,6), −24.8 (d, 2B, *J* = 151 Hz, B2,4), −26.2 (d, 1B, *J* = 164 Hz, B3), −41.6 (d, 1B, *J* = 140 Hz, B1); ^1^H-NMR (600 MHz, CD_3_CN): δ (ppm) = 6.92 (d, 2 H, *J* = 8.4 Hz, ArH), 6.73 (d, 2 H, *J* = 9.0 Hz, ArH), 6.60 (d, 2 H, *J* = 9.0 Hz, ArH), 6.54 (d, 1 H, *J* = 9.0 Hz, ArH), 6.52 (d, 1 H, *J* = 9.0 Hz, ArH), 6.48 (d, 2 H, *J* = 9.0 Hz, ArH), 3.70 (br. s, 2 H, H_2_N), 3.57 (t, 4 H, *J* = 4.2 Hz CH_2_O), 3.5 (br s, 2 H, CH_2_N), 3.47 (t, 2 H, *J* = 4.8 Hz, CH_2_O), 3.43 (t, 2 H, *J* = 4.8 Hz, CH_2_O), 3.38 (t, 2 H, *J* = 4.8 Hz, CH_2_O), 3.11 (t, 2 H, *J* = 5.4 Hz, CH_2_O), 3.09 (t, 2 H, *J* = 5.4 Hz, CH_2_O), 1.58 (br. s, 4 H, CH_carborane_); ^1^H[^11^B]-NMR (400 MHz, CD_3_CN): δ (ppm) = 6.92 (d, 2 H, *J* = 8.4 Hz, ArH), 6.73 (d, 2 H, *J* = 9.0 Hz, ArH), 6.60 (d, 2 H, *J* = 9.0 Hz, ArH), 6.54 (d, 1 H, *J* = 9.0 Hz, ArH), 6.52 (d, 1 H, *J* = 9.0 Hz, ArH), 6.48 (d, 2 H, *J* = 9.0 Hz, ArH), 3.70 (br. s, 2 H, H_2_N), 3.57 (t, 4 H, *J* = 4.2 Hz CH_2_O), 3.5 (br s, 2 H, CH_2_N), 3.47 (t, 2 H, *J* = 4.8 Hz, CH_2_O), 3.43 (t, 2 H, *J* = 4.8 Hz, CH_2_O), 3.38 (t, 2 H, *J* = 4.8 Hz, CH_2_O), 3.11 (t, 2 H, *J* = 5.4 Hz, CH_2_O), 3.09 (t, 2 H, *J* = 5.4 Hz, CH_2_O), 2.17 (br. s, BH), 2.07 (br. s, BH), 1.78 (br. s, 1 H, BH), 1.60 (br. s, 2 H, CH_carborane_), 1.25 (br. s, 1 H, BH), 1.07 (br. s, BH), 0.69 (br. s, 1 H, BH), 0.32, −0.53 (br. s, 1 H, μ-H), −0.61 (br. s, 1 H, μ-H); ^13^C-NMR (100.6 MHz, CD_3_CN) δ (ppm) = 152.4 (ArC), 148.9 (ArC), 148.8 (ArC), 142.4 (ArC), 142.3 (ArC), 140.7 (ArC), 122.9 (ArC), 115.9 (ArC), 115.9 (ArC), 115.8 (ArC), 115.8 (ArC), 114.5 (ArC), 71.6 (CH_2_), 71.4 (CH_2_), 69.4 (CH_2_), 68.7 (CH_2_O), 67.7 (CH_2_O), 55.0 (CH_2_O), 44.6 (CH_2_O), 39.2 (CH_carborane_), 39.2 (CH_carborane_), 38.9 (CH_carborane_);) K.**4**: ^11^B-NMR (192.6 MHz, CD_3_CN) δ (ppm) = −10.4 (s, 1B, B10), −13.5 (d, 2B, *J* = 135 Hz, B9,11), −18.5 (d, 2B, *J* = 133 Hz, B5,6), −24.9 (d, 2B, *J* = 151 Hz, B2,4), −26.2 (d, 1B, *J* = 164 Hz, B3), −41.6 (d, 1B, *J* = 140.18 Hz, B1); ^1^H-NMR (600 MHz, CD_3_CN) δ (ppm) = 6.92 (d, 2 H, *J* = 8.4 Hz, ArH), 6.76 (d, 2 H, *J* = 9.0 Hz, ArH), 3.54 (br. t, 4 H, CH_2_O, CH_2_N), 3.51 (m, 4 H, CH_2_O), 3.45 (t, 4 H, *J* = 4.8 Hz, CH_2_O), 1.55 (br. s, 2 H, CH_carborane_); ^1^H[^11^B]-NMR (400 MHz, CD_3_CN): δ (ppm) −0.61 (br. s, 1 H, μ-H); ^13^C-NMR (100.6 MHz, CD_3_CN) δ (ppm) = 116.9 (ArC), 115.9 (ArC), 71.6 (CH_2_), 71.5 (CH_2_), 69.5 (CH_2_), 55.4 (CH_2_O), 55.2 (CH_2_O), 38.9 (CH_carborane_); MS (ESI^−^) *m*/*z* (%) found: 549.56 (100), 551.52 (15) [M]^−^, calcd. 549.50 (100), 552.49 (14); Analysis found (%): C 36.48, H 7.33, N 2.38, calcd. for C12B9H25O3NK: C 36.82, H 7.38, N 2.39.

[10-(HOCH_2_)_2_CHNH_2_-(CH_2_-CH_2_O)_2_-*nido*-7,8-C_2_B_9_H_11_] (**5**) and [(HOCH_2_)_2_CHNH-*N*,*N*-(10,10′-(CH_2_-CH_2_O)_2_-*nido*-7,8-C_2_B_9_H_11_)_2_]^−^ (**6**^−^)

The reaction of **1** (300 mg, 1.36 mmol) in THF (25 mL) 2-Amino-1,3-propanediol (Serinol) (0.250 g, 2.75 mmol) was carried out in analogous way to the synthesis of **3**. The reaction mixture was stirred at room temperature for 16 h until the spot of starting compound disappeared on the TLC, and then the solvent was removed on a rotary evaporator. The crude product was dissolved in aqueous methanol and the disubstituted compound **6^−^** was precipitated by Me_4_NCl and the solid contained also a small amount of Me_4_N**5.** This mixture was separated by column chromatography on silica gel using CH_2_Cl_2_-CH_3_CN from 5:1 to 3:1 b.v. isolating Me_4_N**6**. The solution was made slightly alkaline by few drops of 5% KOH and the anionic form of compound **5^−^** was precipitated by aqueous Et_4_NCl and left to stand for 1h. The semi-solid material that settled down to glass was separated by decantation, washed with water (4 × 5 mL) and dried in vacuum. The solid material was dissolved in ethyl acetate (30 mL) and treated by diluted HCl (3 M, 30 mL). The organic layer was separated and the aqueous phase extracted by ethyl acetate (2 × 15 mL). Combined organic extracts were evaporated to dryness and the zwitterionic product **5** was then dried in vacuum at 50 °C for 8 h.

**Data for 5:** Semi-solid material, yield 320 mg, 73%: ^11^B-NMR (192.6 MHz, CD_3_CN) δ (ppm) = −11.8 (s, 1B, B10), −13.4 (d, 2B, *J* = 135 Hz, B9,11), −18.4 (d, 2B, *J* = 130 Hz, B5,6), −24.4 (d, 2B, *J* = 149 Hz, B2,4), −25.5 (d, 1B, B3), −41.4 (d, 1B, *J* = 144 Hz, B1); ^1^H-NMR (600 MHz, CD_3_CN): δ (ppm) = 7.56 (q, 2H, *J* = 7.8 Hz, NH), 4.77 (br s, 2 H, OH), 4.29 (dd, 2 H, *J* = 4.2 Hz, CH_2_O), 4,22 (d, 1H, *J* = 5.4 Hz, CH), 3.91 (t, 4 H, *J* = 5.4 Hz, CH_2_O), 3.76 (t, 2 H, *J* = 5.4 Hz, CH_2_O), 3.75 (m, 2 H, CH), 3.64 (t, 2 H, *J* = 4.8 Hz, CH_2_O), 3.28 (br. t, 2 H, *J* = 4.2 Hz, CH_2_O), 1.66 (br. s, 2 H, CH_carborane_); ^1^H[^11^B]-NMR (400 MHz, Acetone-*d*_6_): δ (ppm) = 4.37 (br s, 2 H, OH), 3.80 (br s, 2 H, CH_2_N), 3.71 (br. s, 1 H, CHN), 3.77 (br. t, 2 H, *J* = 7.2 Hz, CH_2_O), 3.59 (br t, 2 H, *J* = 7.2 Hz, CH_2_O), 3.54 (2 H, *J* = 4.2 Hz, CH_2_O), 3.41 (s, 2 H, *J* = 4.2 Hz, CH_2_O), 3.22 (br. t, 4 H, CH_2_O), 2.15 (br. s, 2 H, BH), 1.53 (br. s, 1 H, BH), 1.52 (br. s, 2 H, CH_carborane_), 1.41 (br. s, 2 H, BH), 1.17 (br. s, 2 H, BH), 0.45 (br. s, 1 H, BH), −0.51 (br. s, 2 H, μ-H); ^13^C-NMR (100.6 MHz, Acetone-*d*_6_) δ (ppm) = 70.5 (CH_2_N), 64.8 (CH_2_O), 60.0 (CH_2_O), 55.1 (CHN), 45.2 (CH_2_O), 40.0 (CH_carborane_); MS (ESI^−^) *m*/*z* (%) found: 311.40 (100), 312.40 (52) [M − H]^−^, calcd. 311.28 (100), 312.28 (51); Analysis found (%): C 35.73, H 8.87, N 4.40 calcd. for C9B9H27N1O4 35.59 H 8.72, N 4.49.

**Data for 6^−^:** Viscous semi-solid material, yield 55 mg, 6%: ^11^B-NMR (192.6 MHz, Acetone-*d*_6_) δ (ppm) = −11.0 (s, 2B, B10), −13.4 (d, 4B, *J* = 137 Hz, B9,11), −18.2 (d, 4B, *J* = 129 Hz, B5,6), −24.6 (d, 4B, *J* = 151 Hz, B2,4), −25.8 (d, 2B, B3), −41.3 (d, 2B, *J* = 136 Hz, B1); ^1^H-NMR (600 MHz, Acetone-*d*_6_): δ (ppm) = 7.98 (br s, 1 H, NH), 4.70 (br s, 2 H, OH), 3.96 (dd, 4 H, *J* = 7.8 and 4.2 Hz, CHCH_2_N), 3.88 (t, 4 H, *J* = 7.2 Hz, CH_2_O), 3.85 (q, 4 H, *J* = 6.0 Hz, CH_2_N), 3.61 (s, Me_4_N), 3.58 (t, 4 H, *J* = 5.4 Hz, CH_2_O), 3.48 (m, 1 H, CH), 3.60 (4 H, CH_2_O), 1.53 (br. s, 4 H, CH_carborane_); ^1^H[^11^B]-NMR (400 MHz, Acetone-*d*_6_): δ (ppm) = 7.98 (br s, 1 H, NH), 3.80 (br s, 2 H, CH_2_N), 3.71 (br. s, 1 H, CHN), 3.77 (br. t, 2 H, *J* = 7.2 Hz, CH_2_O), 3.59 (br t, 2 H, *J* = 7.2 Hz, CH_2_O), 3.54 (2 H, *J* = 4.2 Hz, CH_2_O), 3.41 (s, 2 H, *J* = 4.2 Hz, CH_2_O), 3.21 (br. t, 4 H, CH_2_O), 2.151 (br. s, 4 H, BH), 1.57 (br. s, 2 H, BH), 1.48 (br. s, 2 H, CH_carborane_), 1.37 (br. s, 4 H, BH), 1.25 (br. s, 4 H, BH), 0.48 (br. s, 2 H, BH), −0.50 (br. s, 2 H, μ-H); ^13^C-NMR (100.6 MHz, Acetone-*d*_6_) δ (ppm) = 71.3, 70.0, 65.1, 65.0, 58.1 (Me_4_N), 45.1, 38.4 (CH_carborane_); MS (ESI^−^) *m*/*z* (%) found: 531.56 (100), 534.50 (10) [M]^−^, calcd. 531.51 (100), 534.50 (13); Analysis for Ph**_4_**P**6** found (%): C 53.5, H 7.61, N 1.65 calcd. for C39B18H64O6N1P1 C 53.94, H 7.43, N 1.61.

[10-HO-7,8-*nido*-C_2_B_9_H_11_]K (K.**7**)

The zwitterion **1** (300 mg, 1.36 mmol) was poured into 2.5 M KOH (25 mL) and the reaction mixture was stirred and heated to reflux temperature for 3 h. After cooling down the crude product was extracted from the aqueous reaction mixture into ethylacetate (3 × 20 mL). The combined organic extracts were washed with water (20 mL), water (3 mL) was added and the solvent was removed in a rotary evaporator and then the crude product was dried in vacuum for 2 h. The crude product was purified by column chromatography on a silica gel column 25 × 2.5 cm I.D. using CH_2_Cl_2_-CH_3_CN solvent mixture 7:3 b.v.; white solid, yield 195 mg, 76%: ^11^B-NMR (128.32 MHz, CD_3_CN) δ (ppm) = −12.1, −12.5 (s + d, 3B, B10, 9,11), −17.5 (d, 2B, B5,6), −25.1 (d, 2B, B2,4), −27.0 (d, 1B, B3), −41.6 (d, 1B, B1); ^1^H[^11^B]-NMR (600 MHz, CD_3_CN): δ (ppm) = 2.18 (br. s, 2 H, BH), 1.50 (br. s, 1 H, CH_carborane_), 1.45 (br. s, 2 H, BH), 1.37 (br. s, 2 H, BH), 1.14 (br. s, 1 H, BH), 1.434 (br. s, 1 H, BH), −0.38 (br. s, 1 H, μ-H); ^13^C-NMR (100.6 MHz, CD_3_CN) δ (ppm) = 38.3 (CH_carborane_); MS (ESI^−^) *m*/*z* (%) found: 150.32 (100), 151.28 (50), (M-H), calcd. 149.17 (100), 151.17 (48); Analysis found (%): C 12.89, H 6.01, calcd. for C2B9H12OK: C 12.74, H 6.42. The data are in agreement with those reported for synthesis of **7^–^** by another method [60].

[10-HO-(CH_2_CH_2_O)_2_-*nido*-7,8-C_2_B_9_H_11_]K (**K8**)

The zwitterion **1** (300 mg, 1.36 mmol) was dissolved in Et_2_O (30 mL) and then degassed 2.5 M KOH (25 mL) was injected through septum. The reaction mixture was stirred and heated to reflux temperature of ether for 48 h. After cooling down the ether layer was separated, the aqueous phase was extracted twice with ether (10 mL), water (3 mL) was added to combined ether extracts and the organic solvent was evaporated in a rotary evaporator and then dried in vacuum for 2 h. The crude product was purified by column chromatography on a silica gel column 25 × 2.5 cm I.D. using CH_2_Cl_2_-CH_3_CN solvent mixture 9:1 to 2:1 b.v.; white solid, yield 228 mg, 61%; ^11^B-NMR (192.6 MHz, CD_3_CN) δ (ppm) = −11.3 (s, 1B, B10), −13.5 (d, 2B, B9,11), −18.4 (d, 2B, B5,6), −24.6 (d, 2B, B2,4), −25.9 (d, 1B, B3), −41.5 (d, 1B, B1); ^1^H-NMR (600 MHz, CD_3_CN): δ (ppm) = 3.62 (t, 4 H, CH_2_O), 3.492–3.435 (m, 4 H, CH_2_O), 1.604 (br. s, 2 H, CH_carborane_); ^13^C-NMR (100.6 MHz, CD_3_CN) δ (ppm) = 71.4 (CH_2_O), 71.1 (CH_2_O), 69.51 (CH_2_O), 60.9 (CH_2_O), 39.2 (CH_carborane_); MS (ESI^−^) *m*/*z* (%) found: 238.33 (100), 239.25 (45), calcd. 238.23 (100), 239.22 (48); Analysis found for PPh_4_**8** (%): C 62.26, H 6.64, calcd. for C30B9H40O3P1 C 62.46, H 6.99.

[10-(2-CH_3_O-C_6_H_4_O)-(CH_2_-CH_2_O)_2_-*nido*-7,8-C_2_B_9_H_11_]K (K**9**)

The zwitterion **1** (300 mg, 1.36 mmol) was dissolved in dry CH_3_CN (15 mL) and then solid K_2_CO_3_ (1.0 g, 7.25 mmol) was added and 1-HO-2-CH_3_O-C_6_H_4_ (0.3 mL, 2.69 mmol) was injected through septum. The reaction mixture was stirred at room temperature for 24 h, then the excess of K_2_CO_3_ was filtered off under nitrogen and the solvent was removed in vacuum. The residue was dissolved in CH_2_Cl_2_ and poured on top of a silica gel column 25 × 2.2 cm I.D. Elution with CH_2_Cl_2_ led to recovery of a small amount of the starting material. The product was then isolated by continuous elution with CH_2_Cl_2_-CH_3_CN mixture from 9:1 to 1:1 b.v. The respective fractions were evaporated and the product was dried in vacuum for 2.5 h; white solid, yield 185 mg, 40%: ^11^B-NMR (192.6 MHz, CD_3_CN) δ (ppm) = −10.8 (s, 1B, B10), −13.5 (d, 2B, *J* = 132 Hz, B9,11), −18.4 (d, 2B, *J* = 131 Hz, B5,6), −24.7 (d, 2B, *J* = 151 Hz, B2,4), −26.1 (d, 1B, *J* = 164 Hz, B3), −41.6 (d, 1B, *J* = 140 Hz, B1) ^1^H-NMR (600 MHz, CD_3_CN): δ (ppm) = 6.99–6.86 (m, 4 H, ArH), 4.09 (t, 2 H, *J* = 4.2 Hz, CH_2_O), 3.82 (s, 3 H, CH_3_O), 3.73 (t, 2 H, *J* = 4.2 Hz, CH_2_O), 3.61 (br. t, 2 H, *J* = 3.6 Hz, CH_2_O), 3.54 (t, 2 H, *J* = 4.2 Hz, CH_2_O), 1.59 (br. s, 1 H, CH_carborane_); ^1^H[^11^B]-NMR (400 MHz, CD_3_CN): δ (ppm) = 6.99–6.86 (m, 4 H, ArH), 4.09 (t, 2 H, *J* = 4.2 Hz, CH_2_O), 3.82 (s, 1 H, CH_3_O), 3.73 (t, 2 H, *J* = 4.2 Hz, CH_2_O), 3.61 (br. t, 2 H, *J* = 3.6 Hz, CH_2_O), 3.53 (t, 2 H, *J* = 4.2 Hz, CH_2_O), 2.16 (br. s, 2 H, BH), 2.07 (br. s, 1 H, BH), 1.93 (br. s, 2 H, BH), 1.60 (br. s, 2 H, CH_carborane_), 1.25 (br. s, 1 H, BH), 1.09 (br. s, 1 H, BH), −0.57 (br. s, 1 H, μ-H); ^13^C-NMR (100.6 MHz, CD_3_CN) δ (ppm) = 148.7 (ArC), 147.3 (ArC), 121.7 (ArC), 121.2 (ArC), 113.0 (ArC), 111.7 (ArC), 71.4 (CH_3_O), 68.8 (CH_2_O), 68.3 (CH_2_O), 67.6 (CH_2_O), 55.5 (CH_2_O), 39.0 (CH_carborane_); MS (ESI^−^) *m*/*z* (%) found: 344.40 (100), 345.40 (55) [M − H]^−^, calcd. 344.27 (100), 345.27 (53); Analysis found (%): C 40.62, H 7.04, calcd. for C13B9H26O4K C 40.80, H 6.85.

[*^t^*Bu-calix[4]arene-1,3-(10-(CH_2_-CH_2_O)_2_-*nido*-7,8-C_2_B_9_H_11_)_2_]^−^ (**10**^2−^)

Dry acetonitrile (20 mL) was injected into flask containing carefully dried (8 h at 95 °C) *^t^*Bu-calix[4]arene (430 mg, 66 mmol) and solid K_2_CO_3_ (1.0 g, mmol) and the slurry was stirred for 30 min. Then, a solution of compound **1** (300 mg, 1.36 mmol in CH_3_CN, 5 mL) was injected through rubber septum. The reaction mixture was stirred at a temperature of 50 °C for 36 h, then the excess of K_2_CO_3_ was filtered off under nitrogen, washed with CH_3_CN (2 × 5 mL) and the combined organic fractions were evaporated on a vacuum rotary evaporator. The residue was dissolved in ether (30 mL), the solution was filtered through a paper filter from oily brownish impurities and then shaken with diluted HCl (3 M, 3 × 30 mL). Water (25 mL) was added and ether was evaporated with a part of water reducing volume to 20 mL. Methanol was added until dissolution of a turbid solid and the product was precipitated by excess of aqueous CsCl. The voluminous white precipitate was collected and crystallized twice from hot water, to which MeOH was added dropwise until dissolution; the bath was then left slowly cool down. The product was collected by filtration after two days of crystallization, washed with water, and dried in a vacuum for 3 h at room temperature and then at 50 °C for 6 h; white solid, yield 375 mg, 42%: ^11^B-NMR (192.6 MHz, acetone-*d*_6_) δ (ppm) = −10.4 (s, 2B, B10), −14.0 (d, 4B, *J* = 124 Hz, B9,11), −18.2 (d, 4B, *J* = 128 Hz, B5,6), −24.5 (d, 4B, *J* = 146 Hz, B2,4), −26.1 (d, 2B, *J* = 146 Hz, B3), −41.4 (d, 2B, *J* = 137 Hz, B1); ^1^H-NMR (600 MHz, Acetone-*d*_6_): δ (ppm) = 7.18 (s, 4 H, ArH), 6.90 (s, 4 H, ArH), 4.38 (d, 4 H, *J* = 20.4 Hz, CH_2_), 4.20 (m, 4 H, CH_2_O), 3.95 (br. t, 4 H, *J* = 3.6 Hz, CH_2_O), 3.70 (br. t, 8 H, CH_2_O), 3.42 (d, 4 H, *J* = 18.0 Hz, CH_2_), 1.42 (br. s, 2 H, CH_carborane_), 1.27 (s, 18 H, ^t^Bu), 0.91 (s, 18 H, ^t^Bu); ^1^H[^11^B]-NMR (400 MHz, Acetone-*d*_6_): δ (ppm) = 7.18 (s, 4 H, ArH), 6.90 (s, 4 H, ArH), 4.38 (d, 4 H, *J* = 20.4 Hz, CH_2_), 4.20 (m, 4 H, CH_2_O), 3.95 (br. t, 4 H, *J* = 3.6 Hz, CH_2_O), 3.70 (br. t, 8 H, CH_2_O), 3.42 (d, 4 H, *J* = 18.0 Hz, CH_2_), 2.33 (br. s, 4 H, BH), 1.54 (br. s, 4 H, BH), 1.51 (br. s, 4 H, BH), 1.42 (br. s, 2 H, CH_carborane_), 1.18 (br. s, 2 H, BH), 1.27 (s, 18 H, ^t^Bu), 0.91 (s, 18 H, ^t^Bu); 0.51 (br. s, 2 H, BH), −0.40 (br. s, 2 H, μ-H); ^13^C-NMR (100.6 MHz, Acetone-*d*_6_) δ (ppm) = 150.6 (ArC), 146.9 (ArC), 141.6 (ArC), 132.8 (ArC), 127.9 (ArC), 125.8 (ArC), 125.5 (ArC), 75.8 (CH_2_O), 72.9 (CH_2_O), 69.4 (CH_2_O), 69.0 (CH_2_O), 39.1 (CH_carborane_), 33.7 (CH_2_), 33.6 (CH_2_), 31.3 (^t^Bu), 30.6 (^t^Bu); MS (ESI^−^) *m*/*z* (%) found: 544.08 (100), 545.48 (24) [M]^2−^, calcd. 543.93 (100), 545.43 (24); Analysis found (%): C 49.15, H 6.80, calcd. for C56B18H92O8Cs2 C 49.54, H 6.84.

General Procedure for Synthesis of [10-X-(CH_2_-CH_2_O)_2_-*nido*-7,8-C_2_B_9_H_11_]Bu_4_N (X = Cl, Br, I) (**11**–**13**)

Dry diethyl ether (20 mL) was injected into flask containing the starting zwitterion **1** (270 mg, 1.22 mmol) and Bu_4_NX (X=Cl, Br and I) (2.50 mmol). The reaction mixture was stirred at room temperature for 24 h. Ether solutions were the washed with water (4 × 20 mL), separated, evaporated on a rotary evaporator to dryness and then dried for 3 h in vacuum. The products were then crystalized from CH_2_Cl_2_-hexane.

**Data for [10-Cl-(CH_2_-CH_2_O)_2_-*nido-*7,8-C_2_B_9_H_11_]Bu_4_N (11):** White solid, yield 395 mg, 65%: ^11^B-NMR (192.6 MHz, CD_3_CN) δ (ppm) = −10.2 (s, 1B, B10), −13.5 (d, 2B, *J* = 133 Hz, B9,11), −18.5 (d, 2B, *J* = 133 Hz, B5,6), −25.1 (d, 2B, *J* = 148 Hz, B2,4), −26.4 (d, 1B, B3), −41.7 (d, 1B, *J* = 140 Hz, B1); ^1^H-NMR (600 MHz, CD_3_CN): δ (ppm) = 3.63 (dd, 4 H, *J* = 9.6 Hz, CH_2_O), 3.50 (br. t, 2 H, CH_2_O), 3.46 (t, 2 H, *J* = 5.4 Hz, CH_2_Cl), 3.05 (m, 8 H, CH_2_N, Bu_4_N^+^), 1.56 (m, 8 H, CH_2_, Bu_4_N^+^), 1.50 (br. s, 2 H, CH_carborane_), 1.32 (m, 8 H, CH_2_, Bu_4_N^+^), 0.94 (t, 12 H, CH_3_, Bu_4_N^+^); ^1^H[^11^B]-NMR (400 MHz, CD_3_CN): δ (ppm) = 3.63 (dd, 4 H, *J* = 9.6 Hz, CH_2_O), 3.50 (br. t, 2 H, CH_2_O), 3.46 (t, 2 H, *J* = 5.4 Hz, CH_2_), 3.05 (m, 8 H, CH_2_N, Bu_4_N^+^), 1.88 (br. s, 2 H, BH), 1.56 (m, 8 H, CH_2_, Bu_4_N^+^), 1.50 (br. s, 2 H, CH_carborane_), 1.32 (m, 8 H, CH_2_, Bu_4_N^+^), 0.94 (t, 12 H, CH_3_, Bu_4_N^+^), 1.41 (br. s, 2 H, BH), 1.17 (br. s, 2 H, BH), 0.45 (br. s, 1 H, BH), −0.50 (br. s, 1 H, μ-H); ^13^C-NMR (100.6 MHz, CD_3_CN) δ (ppm) = 72.3 (CH_2_), 70.9 (CH_2_), 69.3 (CH_2_), 58.39 (CH_2_N, Bu_4_N^+^), 43.6 (CH_2_), 38.4 (CH_carborane_), 23.5 (CH_2_, Bu_4_N^+^), 19.4 (CH_2_, Bu_4_N^+^), 12.9 (CH_3_, Bu_4_N^+^); MS (ESI^-^) *m*/*z* (%) found: 256.32 (100), 257.28 (65) [M]^−^, calcd. 256.19 (100), 257.19 (67); Analysis found (%): C 52.96, H 10.66, N 2.70 calcd. for C22B9H55O2ClN C 53.01, H 11.12, N 2.80.

**Data for [10-Br-(CH_2_-CH_2_O)_2_-*nido-*7,8-C_2_B_9_H_11_]Bu_4_N (12):** White solid, yield 456 mg, 69%: ^11^B-NMR (192.6 MHz, CD_3_CN) δ (ppm) = −10.2 (s, 1B, B10), −13.5 (d, 2B, *J* = 135 Hz, B9,11), −18.5 (d, 2B, *J* = 130 Hz, B5,6), −25.1 (d, 2B, *J* = 148 Hz, B2,4), −26.4 (d, 1B, *J* = 161 Hz, B3), −41.7 (d, 1B, *J* = 137 Hz, B1); ^1^H-NMR (600 MHz, CD_3_CN): δ (ppm) = 3.71 (t, 2 H, *J* = 6.0 Hz, CH_2_O), 3.49 (br t, 2 H, CH_2_O), 3.47 (m, 4 H, *J* = 9.6 Hz, CH_2_O, CH_2_Br), 3.06 (m, 8 H, CH_2_N, Bu_4_N^+^), 1.57 (m, 8 H, CH_2_, Bu_4_N^+^), 1.50 (br. s, 2 H, CH_carborane_), 1.32 (m, 8 H, CH_2_, Bu_4_N^+^), 0.94 (t, 12 H, CH_3_, Bu_4_N^+^); ^1^H[^11^B]-NMR (400 MHz, CD_3_CN): δ (ppm) = 3.71 (t, 2 H, *J* = 6.0 Hz, CH_2_O), 3.49 (br t, 2 H, CH_2_O), 3.47 (m, 4 H, *J* = 9.6 Hz, CH_2_O, CH_2_Br), 3.06 (m, 8 H, CH_2_N, Bu_4_N^+^), 2.21 (br. s, 1 H, BH), 1.57 (m, 8 H, CH_2_, Bu_4_N^+^), 1.50 (br. s, 2 H, CH_carborane_), 1.32 (m, 8 H, CH_2_, Bu_4_N^+^), 1.03 (br. s, 4 H, BH) 0.94 (t, 12 H, CH_3_, Bu_4_N^+^); 0.26 (br. s, 1 H, BH), −0.64 (br. s, 1 H, μ-H); ^13^C-NMR (100.6 MHz, CD_3_CN) δ (ppm) = 71.9 (CH_2_), 70.8 (CH_2_), 69.3 (CH_2_), 58.4 (CH_2_N, Bu_4_N^+^, CH_2_), 38.2 (CH_carborane_), 23.4 (CH_2_, Bu_4_N^+^), 19.4 (CH_2_, Bu_4_N^+^), 13.0 (CH_3_, Bu_4_N^+^); MS (ESI^−^) *m*/*z* (%) found: 300.32 (100), 302.20 (90) [M]^−^, calcd. 300.15 (100), 301.14 (92); Analysis found (%): C 48.61, H 10.18, N 2.52 calcd. for C22B9H55O2BrN C 48.67, H 10.21, N 2.58.

**Data for [10-I-(CH_2_-CH_2_O)_2_-*nido-*7,8-C_2_B_9_H_11_]Bu_4_N (13):** White solid, yield 516 mg, 71%: ^11^B-NMR (192.6 MHz, CD_3_CN) δ (ppm) = −10.4 (s, 1B, B10), −13.5 (d, 2B, *J* = 135 Hz, B9,11), −18.5 (d, 2B, *J* = 133 Hz, B5,6), −25.0 (d, 2B, *J* = 149 Hz, B2,4), −26.3 (d, 1B, *J* = 169 Hz, B3), −41.6 (d, 1B, *J* = 138 Hz, B1); ^1^H-NMR (600 MHz, CD_3_CN): δ (ppm) = 3.66 (t, 2 H, *J* = 6.0 Hz, CH_2_O), 3.53 (br t, 2 H, CH_2_O), 3.47 (t, 2 H, *J* = 5.4 Hz, CH_2_O), 3.26 (t, 2 H, *J* = 6.3 Hz, CH_2_I), 3.05 (m, 8 H, CH_2_N, Bu_4_N^+^), 1.57 (m, 8 H, CH_2_, Bu_4_N^+^), 1.52 (br. s, 2 H, CH_carborane_), 1.33 (m, 8 H, CH_2_, Bu_4_N^+^), 0.94 (t, 12 H, CH_3_, Bu_4_N^+^); ^1^H[^11^B]-NMR (400 MHz, CD_3_CN): δ (ppm) = 3.66 (t, 2 H, J = 6.0 Hz, CH_2_O), 3.53 (br t, 2 H, CH_2_O), 3.47 (t, 2 H, *J* = 5.4 Hz, CH_2_O), 3.26 (t, 2 H, *J* = 6.3 Hz, CH_2_I), 3.08 (m, 8 H, CH_2_N, Bu_4_N^+^), 1.88 (br. s, 1 H, BH), 1.56 (m, 8 H, CH_2_, Bu_4_N^+^), 1.50 (br. s, 2 H, CH_carborane_), 1.58 (m, 8 H, CH_2_, Bu_4_N^+^), 1.53 (br. s, 4 H, BH), 1.20 (br. s, 2 H, BH), 1.01 (t, 12 H, CH_3_, Bu_4_N^+^), 0.97 (br. s, 1 H, BH), 0.23 (br. s, 1 H, BH), −0.64 (br. s, 1 H, μ-H); ^13^C-NMR (100.6 MHz, CD_3_CN) δ (ppm) = 71.5 (CH_2_O), 71.4 (CH_2_), 69.4 (CH_2_), 58.4 (CH_2_N, Bu_4_N^+^, CH_2_), 38.9 (CH_carborane_), 23.4 (CH_2_, Bu_4_N^+^), 19.4 (CH_2_, Bu_4_N^+^), 13.0 (CH_3_, Bu_4_N^+^); MS (ESI^-^) *m*/*z* (%) found: 348.16 (100), 349.16 (45) [M]^−^, calcd. 348.13 (100), 349.13 (48); Analysis found (%): C 44.62, H 9.38, N 2.34 calcd. for C22B9H55O2IN C 44.80, H 9.40, N 2.37.

[10-HS-(CH_2_-CH_2_O)_2_-*nido*-7,8-C_2_B_9_H_11_]^−^ (14^−^) and [S-(10,10’-(CH_2_-CH_2_O)_2_-*nido*-7,8-C_2_B_9_H_11_)_2_]^2−^

Distilled THF (60 mL) was injected into flask containing the starting zwitterion **1** (423 mg, 1.92 mmol). 724 mg of NaHS.H_2_O was added the reaction mixture was stirred at room temperature for 24 h. The mixture was extracted by ether, evaporated on a rotary evaporator to dryness and then dried for 3 h in vacuum. This mixture was separated by column chromatography on silica gel using CH_2_Cl_2_-CH_3_CN from 5:1 to 1:1 b.v. isolating **14^−^** and **15^2−^**. **14^−^** and **15^2−^** were dried under vacuum for 8 h at 80 °C.

**Data for** Na**14:** White solid, yield 137 mg, 26%: ^11^B-NMR (192.6 MHz, CD_3_CN) δ (ppm) = −10.5 (s, 1B, B10), −13.4 (d, 2B, *J* = 137 Hz, B9,11), −18.5 (d, 2B, *J* = 133 Hz, B5,6), −24.9 (d, 2B, *J* = 150 Hz, B2,4), −26.2 (d, 1B, *J* = 168 Hz, B3), −41.6 (d, 1B, *J* = 142 Hz, B1); ^1^H-NMR (600 MHz, CD_3_CN): δ (ppm) = 3.51 (t, 4 H, *J* = 6.0 Hz, CH_2_O), 3.44 (t, 2 H, *J* = 5.0 Hz, CH_2_O), 2.62 (q, 2 H, *J* = 7.0 Hz, CH_2_S), 1.70 (t, 1H, *J* = 8.0 Hz, SH), 1.54 (br. s, 2 H, CH_carborane_). ^1^H[^11^B]-NMR (400 MHz, CD_3_CN): δ (ppm) = 3.51 (t, 4 H, *J* = 6.0 Hz, CH_2_O), 3.44 (t, 2 H, *J* = 5.0 Hz, CH_2_O), 2.62 (q, 2 H, *J* = 7.0 HzHz, CH_2_S), 1.70 (t, 1H, *J* = 8.0 Hz, SH), 2.03 (br. s, BH), 1.54 (br. s, 2 H, CH_carborane_), 1.33 (br. s, BH), 1.21 (br. s, BH), 1.02 (br. s, BH), −0.61 (br. s, 1 H, μ-H); ^13^C-NMR (100.6 MHz, CD_3_CN) δ (ppm) = 72.4, 71.4, 69.1, 38.4 (CH_carborane_), 23.69. MS (ESI^−^) *m*/*z* (%) found: 254.32 (100), 255.32 (45) [M]^−^, calcd. 254.21 (100), 255.20 (48); Analysis found (%): C 25.97, H 7.02 calcd. for C6B9H20O2SNa C 26.06, H 7.29.

**Data for** Na_2_**15:** White solid, yield 27 mg, 5%: ^11^B-NMR (192.6 MHz, CD_3_CN) δ (ppm) = ^11^B-NMR (192.6 MHz, CD_3_CN) δ (ppm) = −10.8 (s, 2B, B10), −13.4 (d, 4B, *J* = 137 Hz, B9,11), −18.5 (d, 4B, *J* = 133 Hz, B5,6), −24.9 (d, 4B, *J* = 150 Hz, B2,4), −26.2 (d, 2B, *J* = 168 Hz, B3), -41.6 (d, 2B, *J* = 161 Hz, B1); ^1^H-NMR (600 MHz, CD_3_CN): δ (ppm) = 3.59 (t, 2 H, *J* = 7.0 Hz, CH_2_O), 3.56 (br. t, 2 H, CH_2_O), 3.48 (t, 2 H, *J* = 5.0 Hz), 2.71 (t, 2H, *J* = 6.0 Hz, CH_2_S), 1.57 (br. s, 2 H, CH_carborane_). ^1^H[^11^B]-NMR (400 MHz, CD_3_CN): δ (ppm) = 3.59 (t, 2 H, *J* = 7.0 Hz, CH_2_O), 3.56 (br. t, 2 H, CH_2_O), 3.48 (t, 2 H, *J* = 5.0 Hz), 2.71 (t, 2H, *J* = 6.0 Hz, CH_2_S), 2.06 (br. s, BH), 1.35 (br. s, BH), 1.57 (br. s, 2 H, CH_carborane_), 1.23 (br. s, BH), 1.04 (br. s, BH), −0.55 (br. s, 1 H, μ-H); ^13^C-NMR (100.6 MHz, CD_3_CN) δ (ppm) = 71.3, 69.7, 68.9, 38.9 (CH_carborane_), 31.2. MS (ESI^−^) *m*/*z* (%) found: 496.56 (100), 497.60 (72) [M]^−^, calcd. 496. 42 (100), 497.41 (82); Analysis found (%): C 27.46, H 7.41 calcd. for C12B18H38O4S1Na2 C 27.77, H 7.38.

## 4. Conclusions

In this article we have been focussing on the basic chemistry of the dioxane derivative **1** of the [*nido*-C_2_B_9_H_12_]^−^ ion. Ring cleavage reactions with various nucleophilic reagents have been explored, revealing various patterns in reactivity that we have documented in this manuscript. Unlike in previously reported reactions of compounds that contain a cyclic ether attached via an oxonium atom to other (*closo-*) boron cages, the zwitterion **1** exhibits some different features that apparently are a consequence of its *nido*-structure. These differences are connected with the presence of the extra hydrogen atom, its unexpectedly easy abstraction from the molecule and dual reaction pathways observed for the resulting anion, which exchanges dioxane ring with OH^−^ as the nucleophile. On the other hand, attacks of nucleophiles such as NH_2_R, Cl^−^, Br^−^, I^−^, S^−^, SH^−^, and RO^−^ proceed at the carbon atom in the α-position relative to the oxonium atom of the ether ring and produce compounds in which the terminal nucleophile moiety is bound to the polyhedral cage via a six-atom diethyleneglycol pendant group. Therefore, the neutral compound **1** and products of its reactions described herein can serve as valuable building blocks for a variety of purposes in the construction of advanced functional molecules, materials, compounds for BNCT, drug design, etc. They also offer the possibility to serve as ligands in synthesis of a broad range of new metal dicarbollide complexes. The advantage of all the compounds described herein is the symmetric position of the substituent on the cage, which may be of high relevance for the synthesis of biologically active compounds.

## Supplementary Materials

The following are available online: CIF file for crystallographic structures of compounds **1** and **2**, Crystallographic collection data, Table S1: NMR and MS spectral data for compounds **2** to **15**, Figures S1–S16 and Figures S17–S33, resp.
