# Supplementary material for: Focus on Chemistry of the 10-Dioxane-nido-7,8-dicarba-undecahydrido Undecaborate Zwitterion; Exceptionally Easy Abstraction of Hydrogen Bridge and Double-Action Pathways Observed in Ring Cleavage Reactions with OH− as Nucleophile
Source: Molecules. 2020 Feb 13;25(4):814. doi: 10.3390/molecules25040814 (PMC7070711; doi:10.3390/molecules25040814)
Supplement: Supplementary file 1 [file molecules-25-00814-s001.zip › Supplementary material/Supplementary Material-C2B9dioxane F.pdf]

Supporting Material

Focus on chemistry of the 10-dioxane-*nido*- 7,8-dicarba-undecahydrido undecaborate zwitterion; exceptionally easy abstraction of hydrogen bridge and double-action pathways observed in ring cleavage reactions with OH^- as nucleophile.

Mário Bakardjiev [1,#], Suzan El Anwar[1,#], Dmytro Bavol[1], Zdeňka Růžičková[2] and Bohumír Grűner[1,*]

[1] Institute of Inorganic Chemistry of Czech Academy of Sciences, 25068 Řež, Czech Republic, gruner@iic.cas.cz

[2] Faculty of Chemical Technology, University of Pardubice, Studentská 95, Pardubice, Czech Republic.

* Correspondence: gruner@iic.cas.cz
[#] These authors contributed equally

Content

Crystallography…………………………………………….……………… 2

NMR Spectra of compounds 1-15, Figs. S1-S16……………………….. 3-26

MS Spectra of compounds 1-15, Figs. S17-S30……………………… ... 27-33

I. Crystallography

Table 1. Data collection and structure refinement for compounds **1** and **2**.

**Theta range for data collection**	2.37 to 27.51°	2.11 to 27.54°
**Index ranges**	-9<=h<=10, -14<=k<=14, -17<=l<=16	-9<=h<=9, -16<=k<=16, -19<=l<=19
**Reflections collected**	12334	32867
**Independent reflections**	2895 [R(int) = 0.0587]	3123 [R(int) = 0.0548]
**Coverage of independent reflections**	99.8%	99.8%
**Absorption correction**	Multi-Scan	Multi-Scan
**Structure solution technique**	direct methods	direct methods
**Structure solution program**	XT, VERSION 2014/5	XT, VERSION 2014/5
**Refinement method**	Full-matrix least-squares on F^2	Full-matrix least-squares on F^2
**Refinement program**	SHELXL-2014/7 (Sheldrick, 2014)	SHELXL-2014/7 (Sheldrick, 2014)
**Function minimized**	$\Sigma w(F_o^2 - F_c^2)^2$	$\Sigma w(F_o^2 - F_c^2)^2$
**Data / restraints / parameters**	2895 / 0 / 198	3123 / 0 / 208
**Goodness-of-fit on F^2**	1.079	1.171
**Final R indices**	2487 data; I>2σ(I), R1 = 0.0442, wR2 = 0.0928	2655 data; I>2σ(I), R1 = 0.0521, wR2 = 0.1296
	all data R1 = 0.0564, wR2 = 0.0978	all data R1 = 0.0637, wR2 = 0.1349
**Weighting scheme**	$w=1/[\sigma^2(F_o^2)+(0.0501P)^2+0.1134P]$ where $P=(F_o^2+2F_c^2)/3$	$w=1/[\sigma^2(F_o^2)+(0.0549P)^2+0.7643P]$ where $P=(F_o^2+2F_c^2)/3$
**Largest diff. peak and hole**	0.143 and -0.268 eÅ^{-3}	0.284 and -0.227 eÅ^{-3}
**R.M.S. deviation from mean**	0.051 eÅ^{-3}	0.056 eÅ^{-3}

II. NMR Spectra

Deprotonation of compound 1⁻

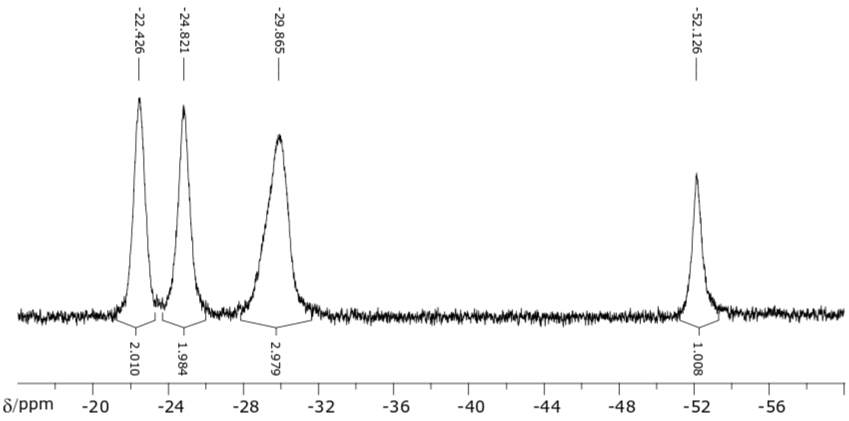

Fig S1a. ^{11}B NMR of deprotonated anion 1^- (in CD_3CN-D_2O)

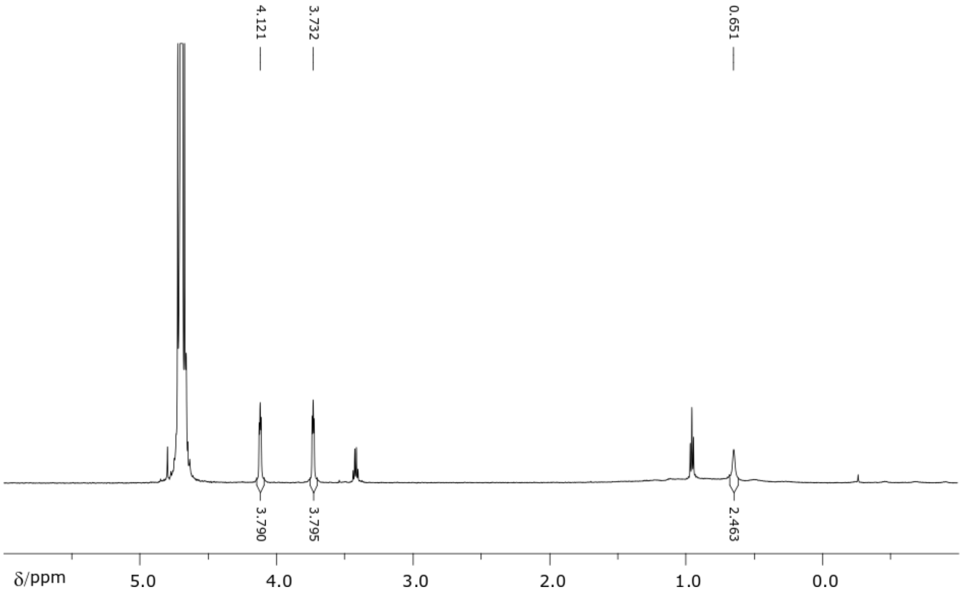

Fig S1b. ^{1}H NMR of deprotonated anion **1^-** (in CD_3CN-D_2O)

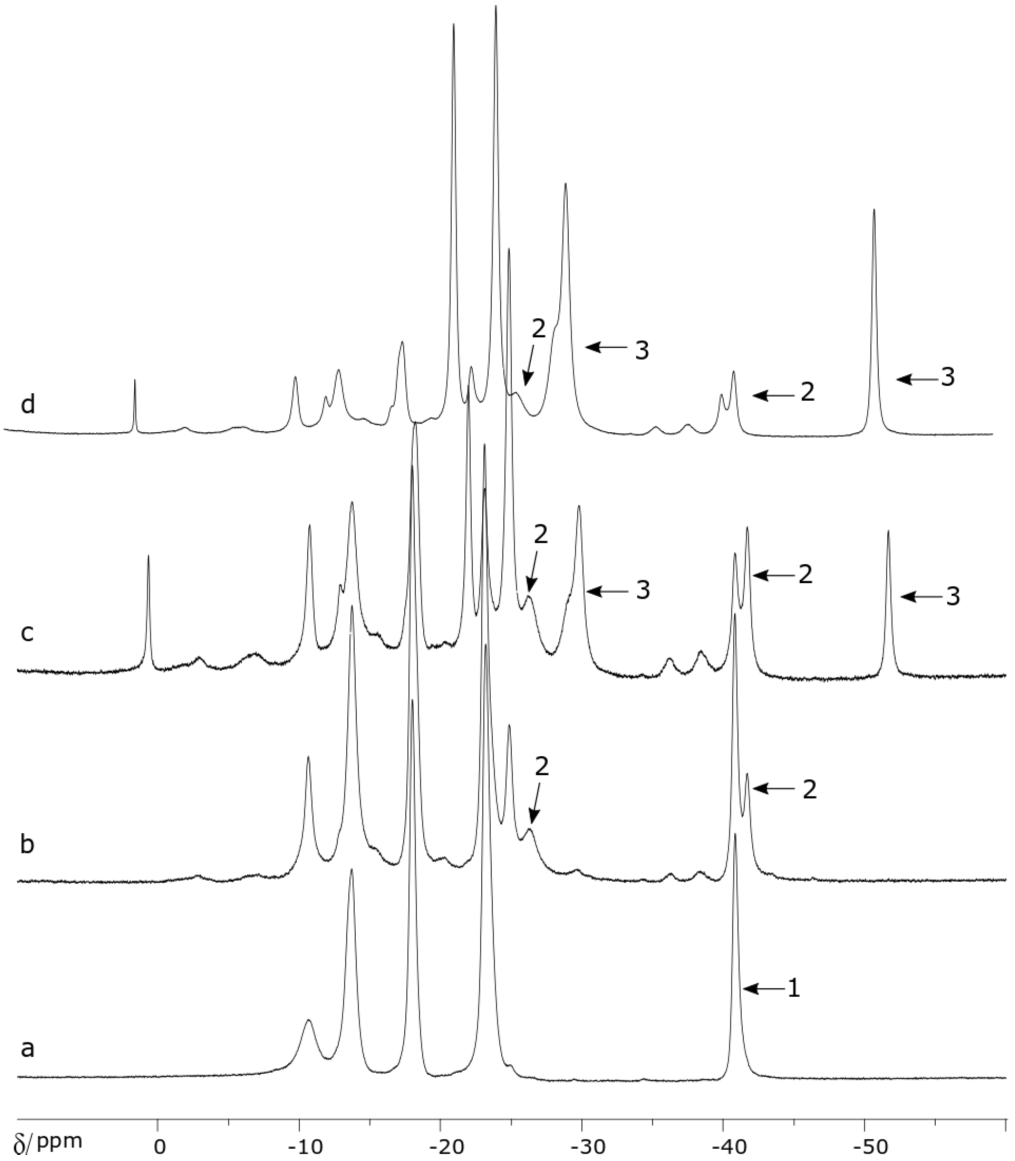

Fig S2. ^{11}B NMR spectrum of deprotonated anion 1^{-} (in THF_8-D_2O 1: 1 b.v.), from bottom to top: a compound 1, b 1 + ½ eq KOH, c 1 + 1 eq KOH, d 1 + 1.5 eq KOH, peaks marked with numbers 2 and 3 correspond: 2- exchange of extra hydrogen atom for deuterium in from D_2O, 3- to the deprotonated anionic form **1**$^{-}$.

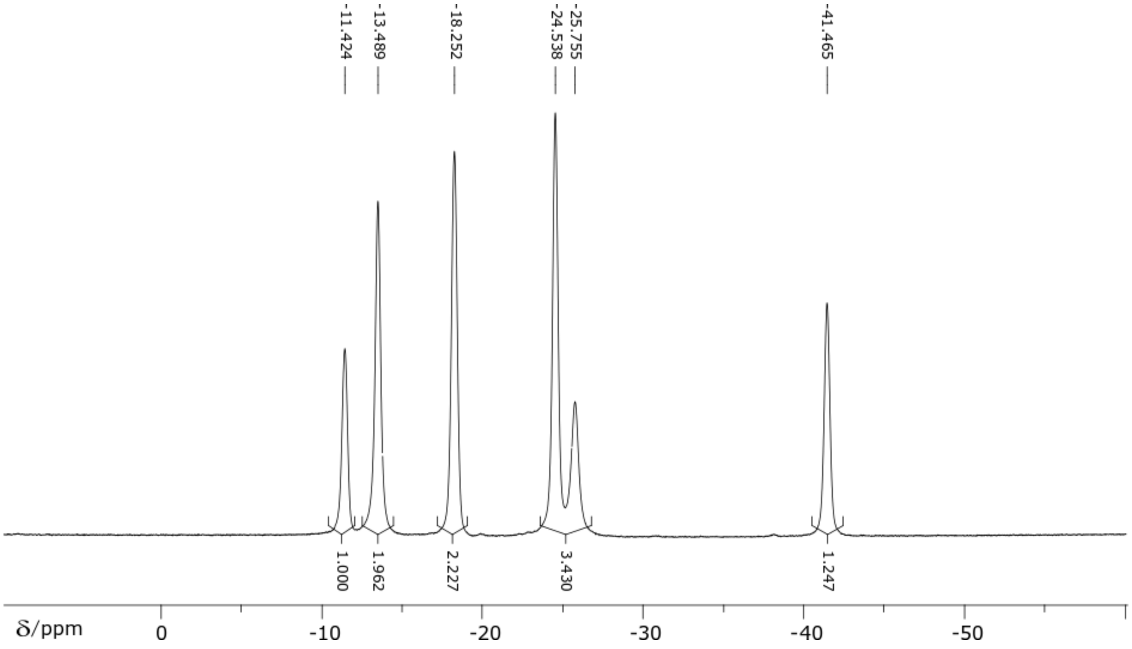

Fig S3a. ^{11}B NMR of compound 2

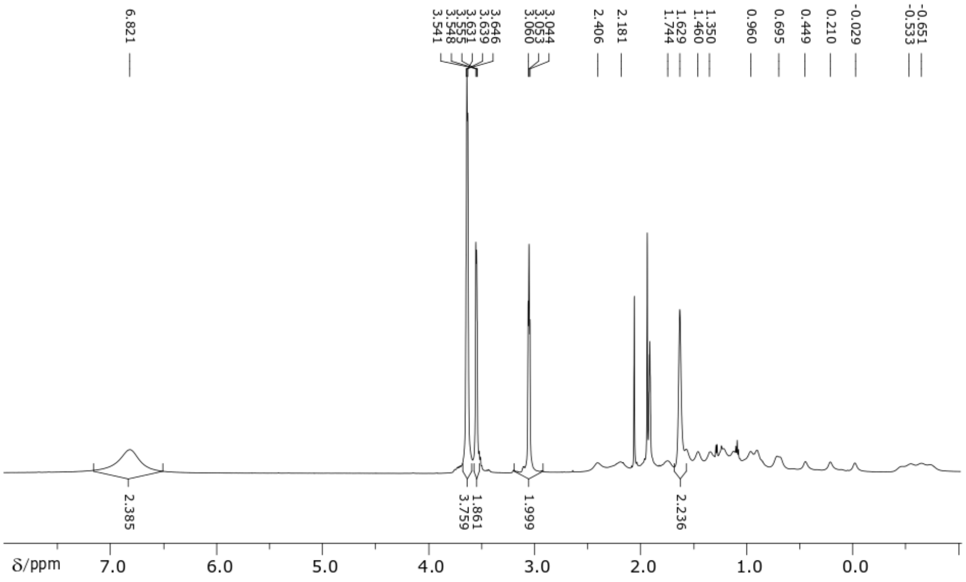

Fig S3b. ^{1}H NMR of compound 2

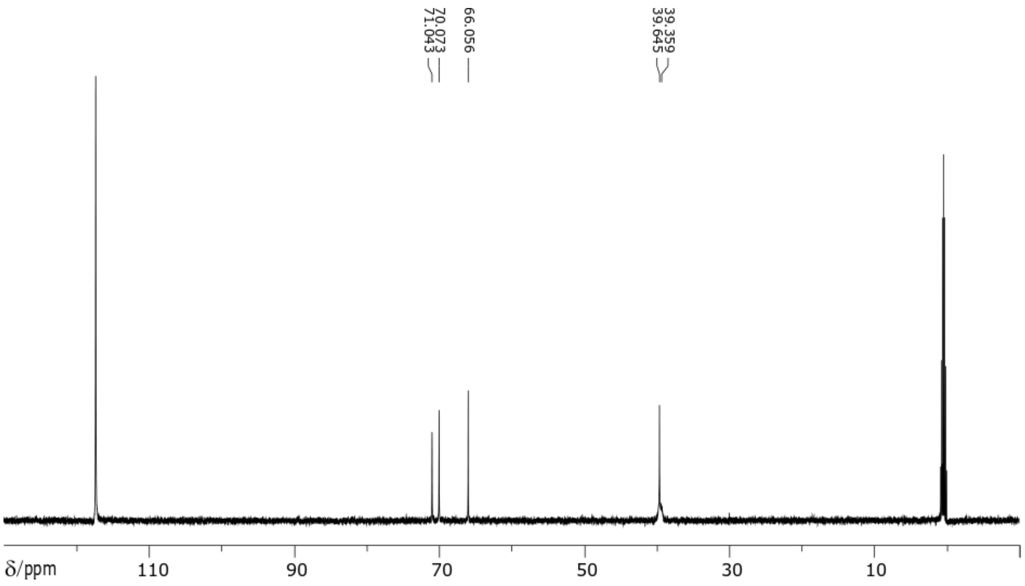

Fig S3c. ^{13}C NMR of compound **2**

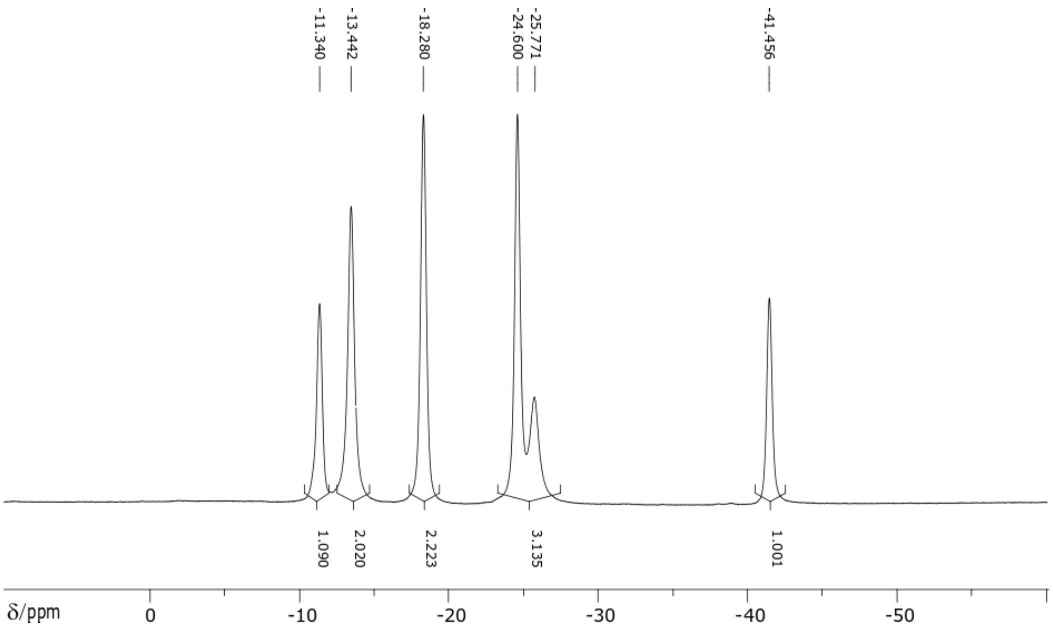

Fig S4a. ^{11}B NMR of compound **3**

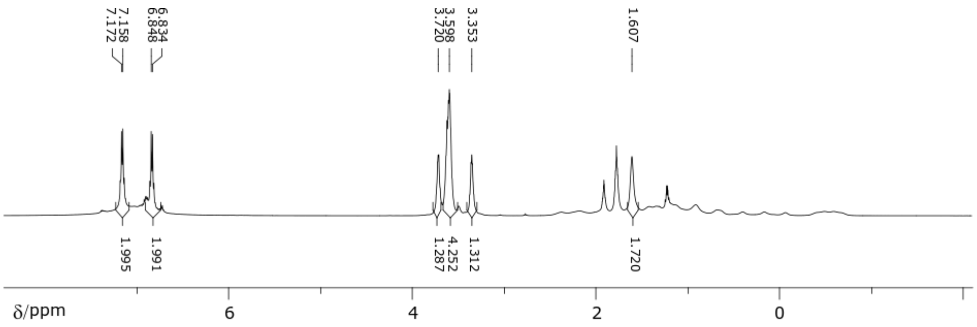

Fig S4b. ^{1}H NMR of compound **3**

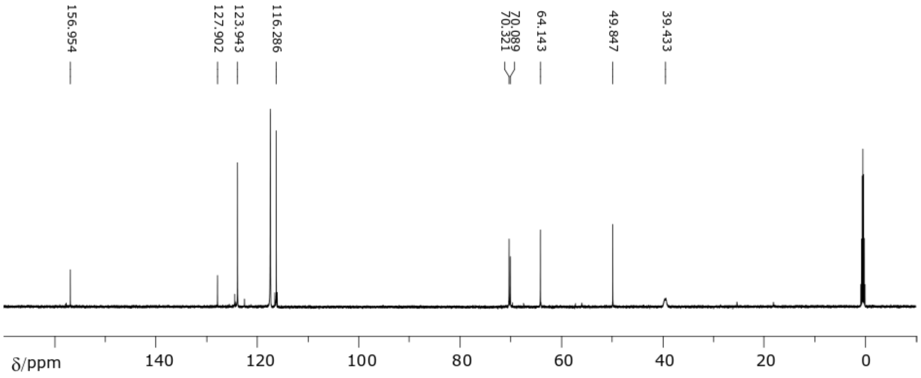

Fig S4c. ^{13}C NMR of compound **3**

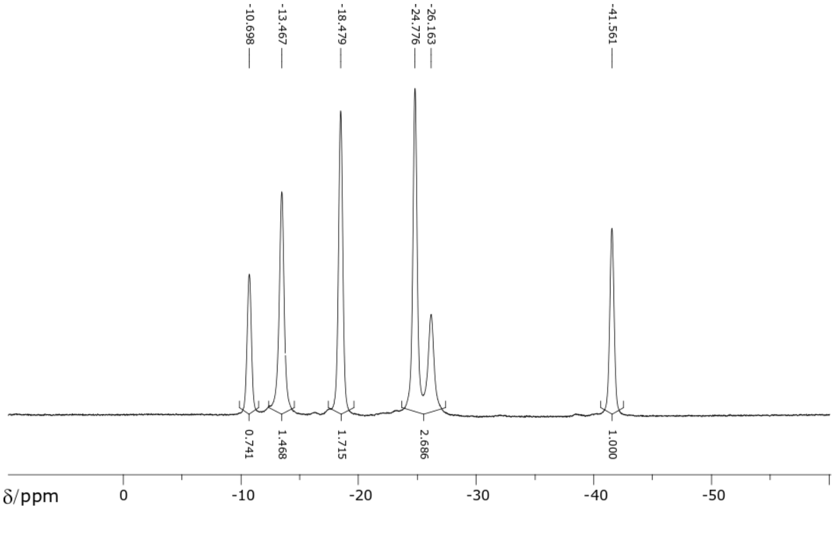

Fig S5a. ^{11}B NMR of compound $[HOC_6H_4NH_3]$**4**

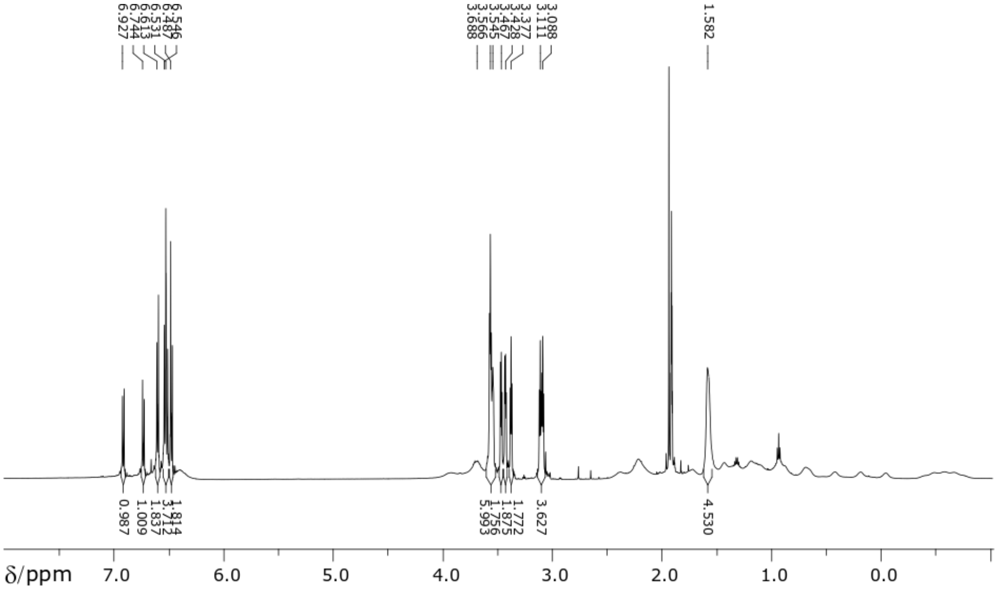

Fig S5b. ^{1}H NMR of compound $[HOC_6H_4NH_3]$**4**

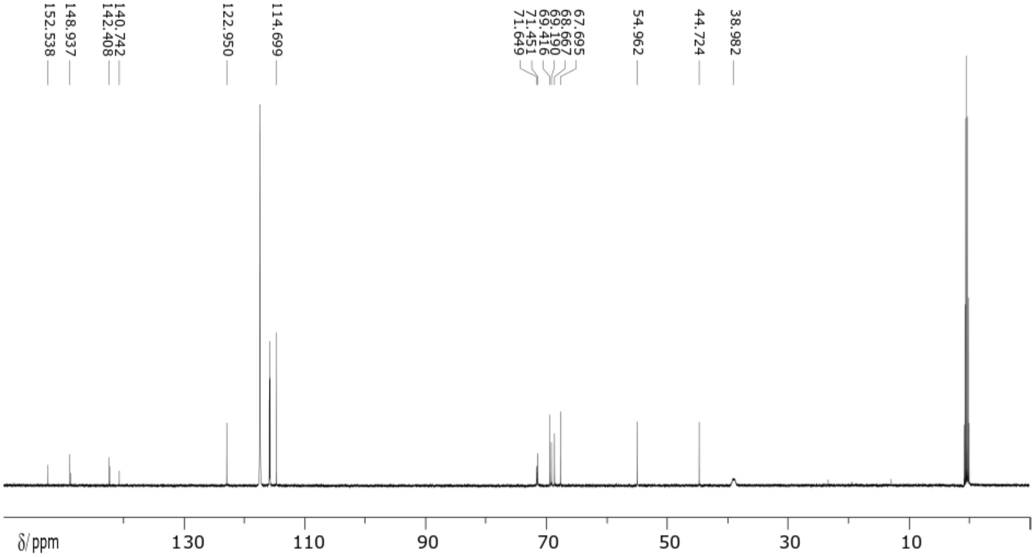

Fig S5c. ^{13}C NMR of compound $[HOC_6H_4NH_3]$**4**

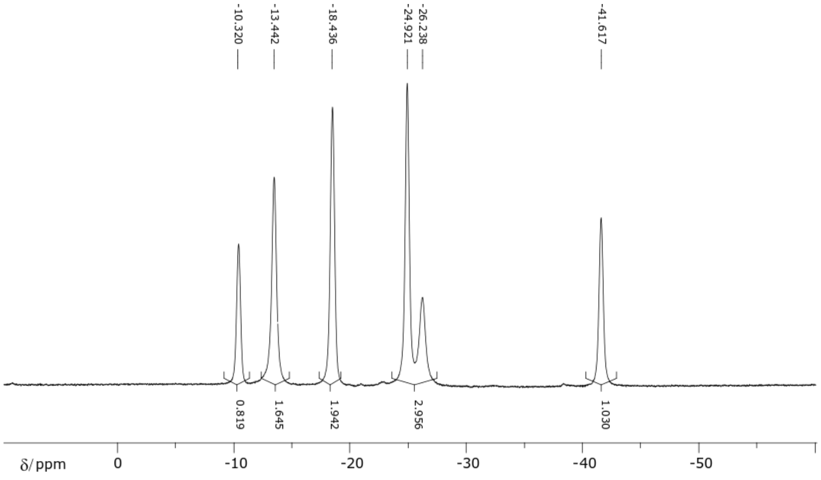

Fig S5d. ^{11}B NMR of compound K**4**

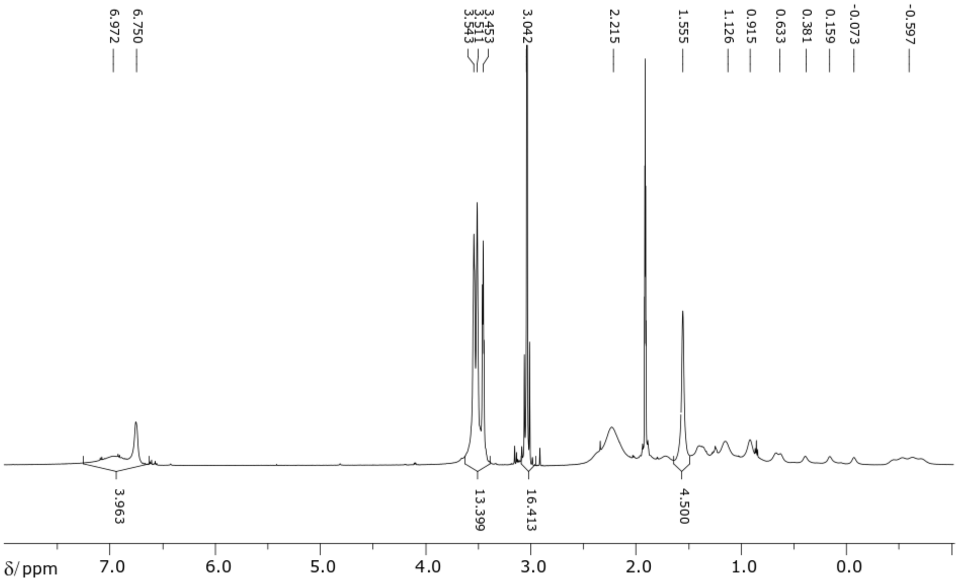

Fig S5e. ^{1}H NMR of compound K**4**

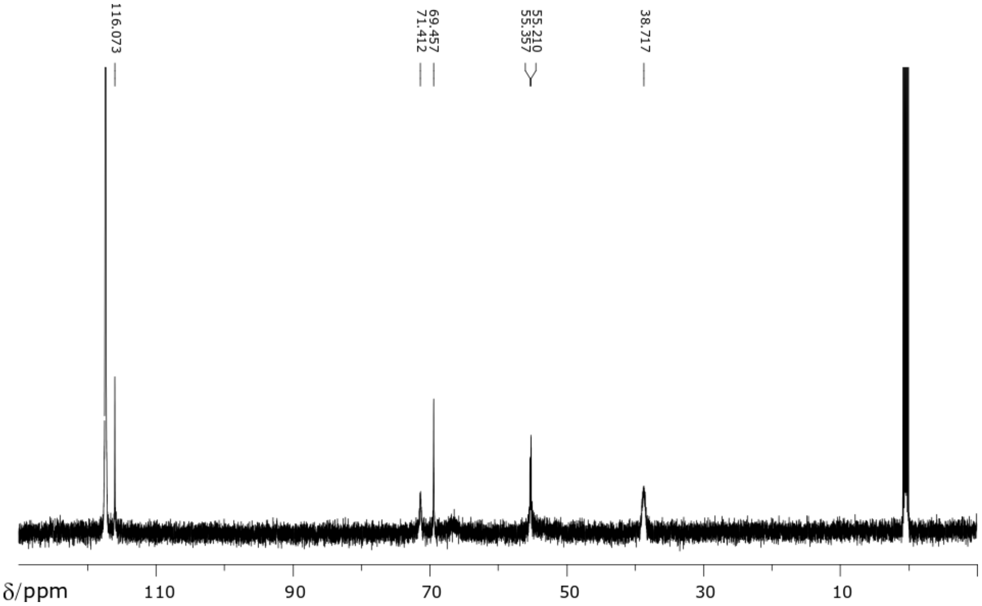

Fig S5f. ^{13}C NMR of compound K**4**

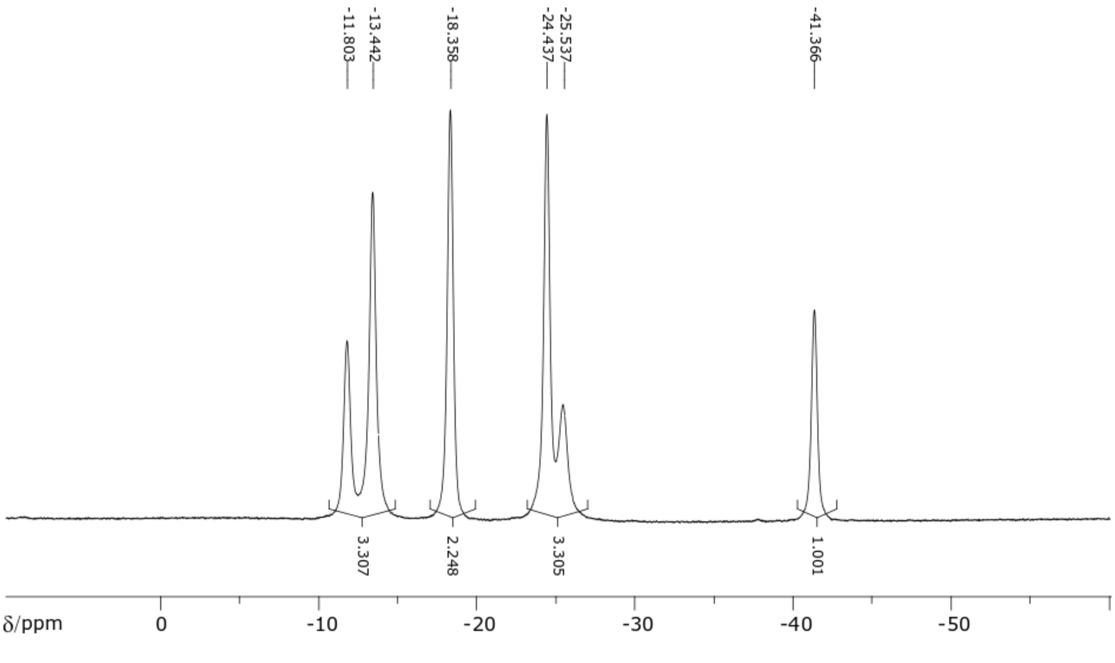

Fig S6a. ^{11}B NMR of compound 5 **(zwitterion)**

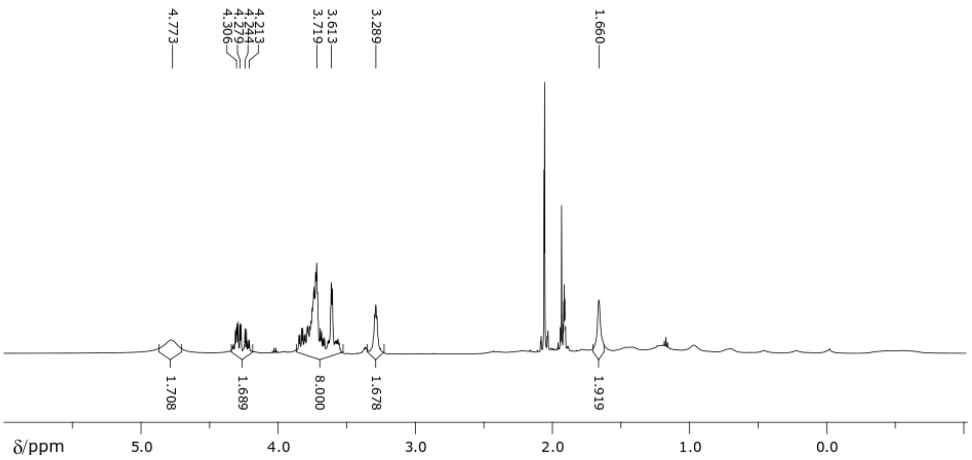

Fig S6b. ^{1}H NMR of compound 5 **(zwitterion)**

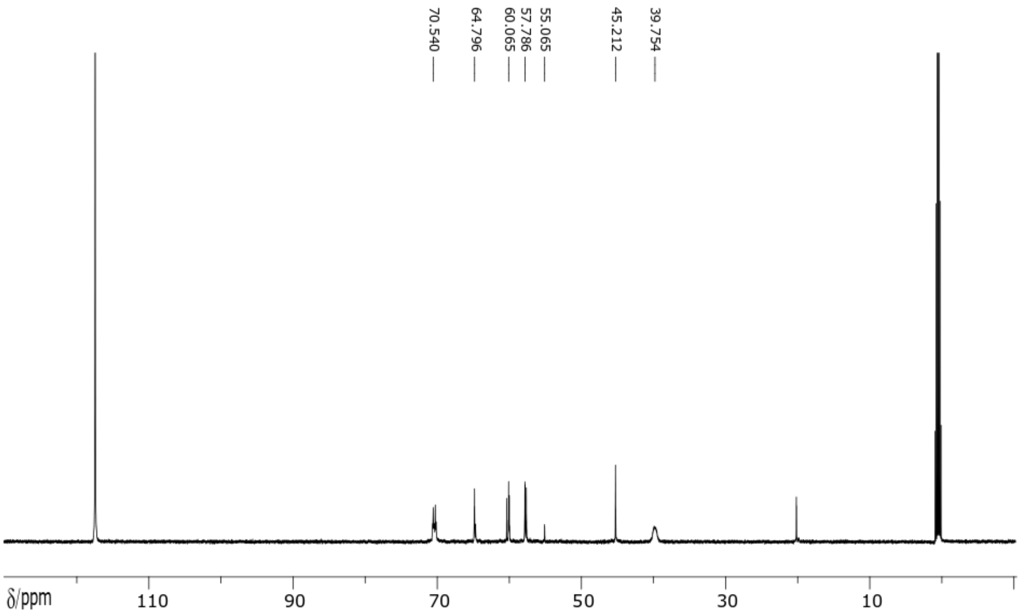

Fig S6c. ^{13}C NMR of compound 5 **(zwitterion)**

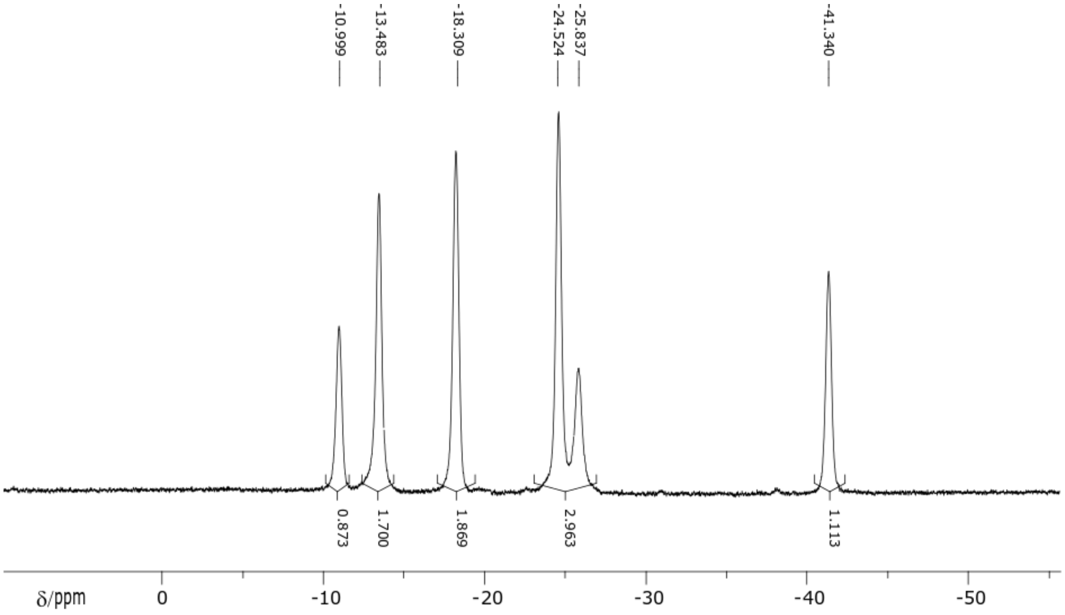

Fig S7a. ^{11}B NMR of compound **6**$^{-}$

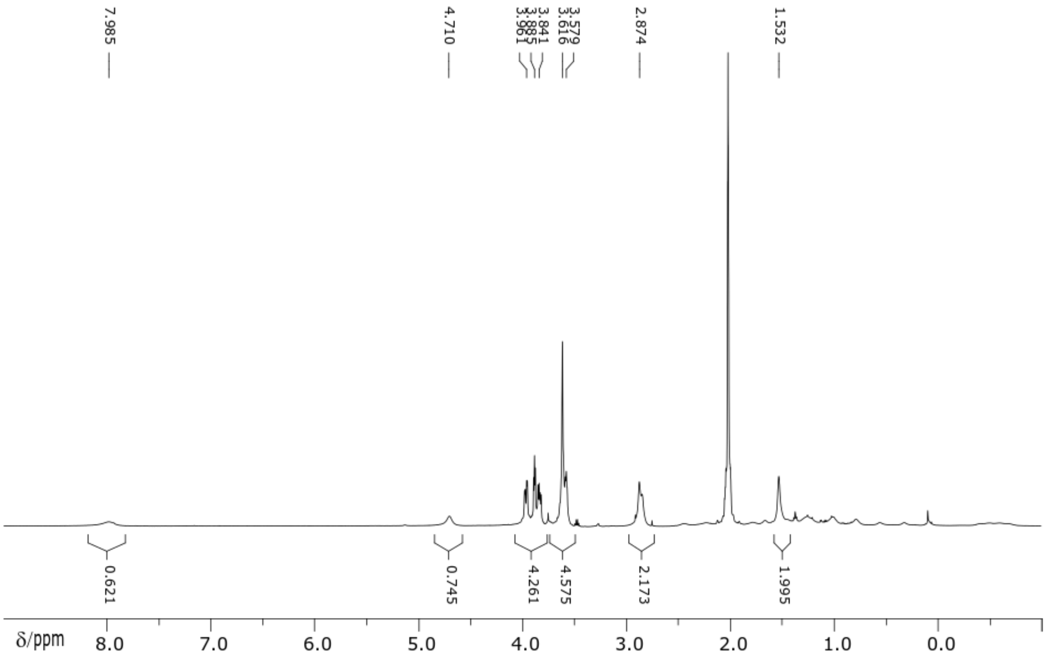

Fig S7b. ^{1}H NMR of compound **6**$^{-}$

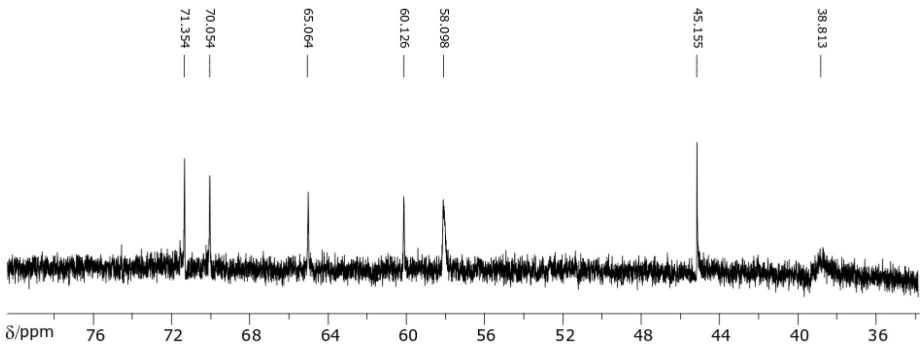

Fig S7c. ^{13}C NMR of compound **6**$^{-}$

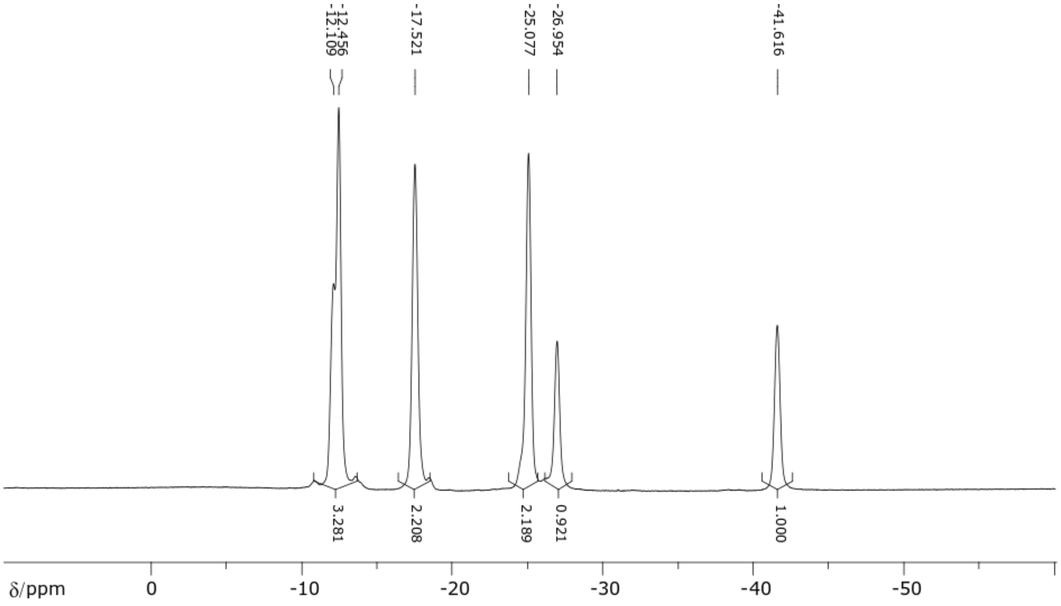

Fig S8a. ^{11}B NMR of compound 7^{-}

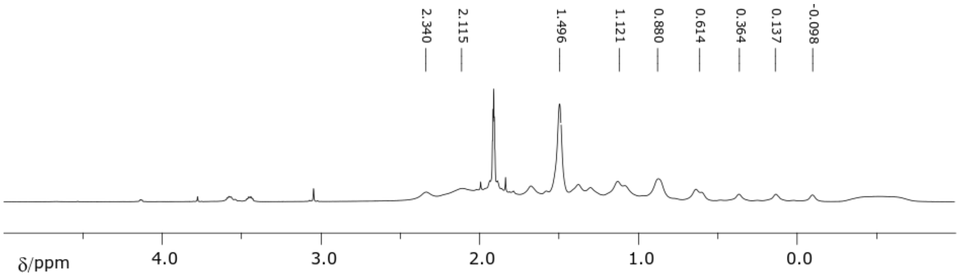

Fig S8b. ^{1}H NMR of compound **7**$^{-}$

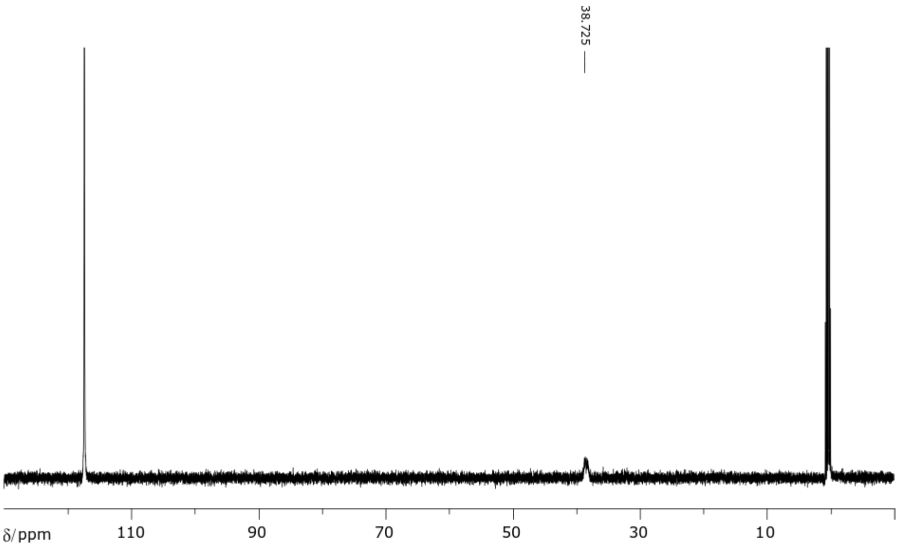

Fig S8c. ^{13}C NMR of compound **7**$^{-}$

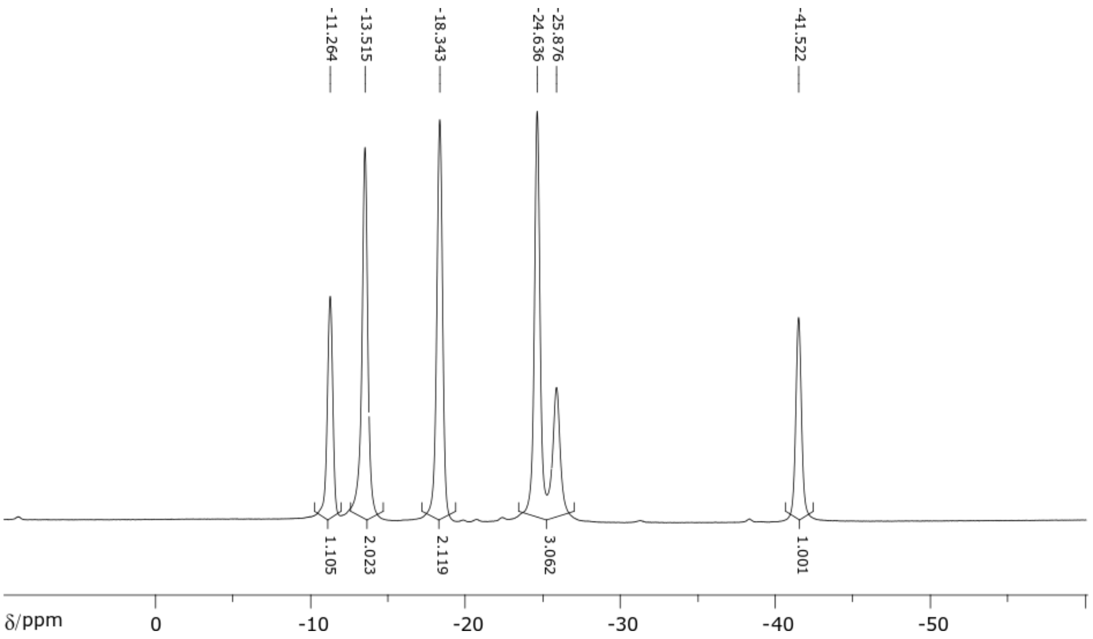

Fig S9a. ^{11}B NMR of compound **8**$^{-}$

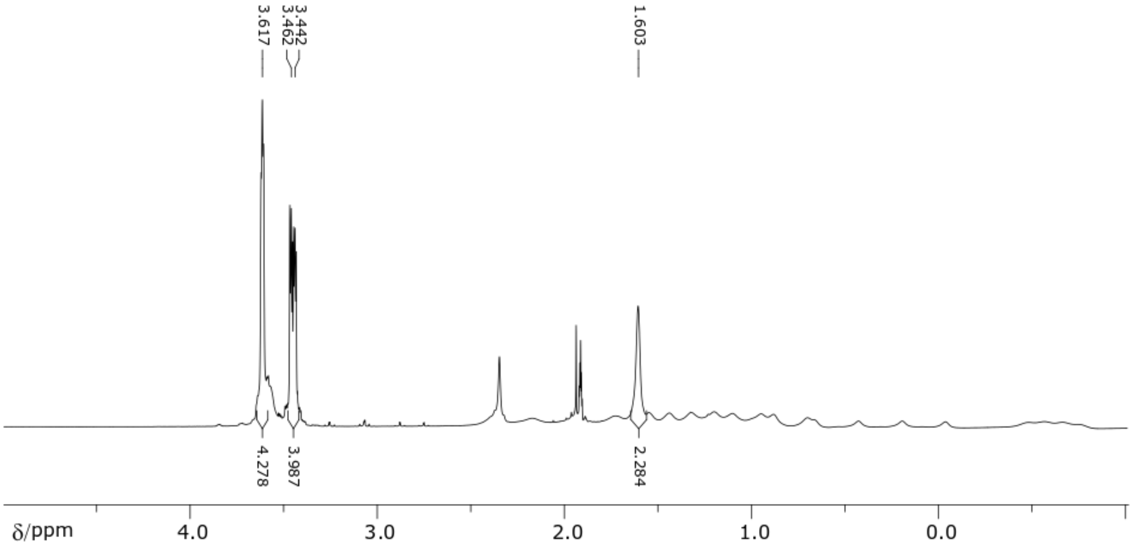

Fig S9b. ^{1}H NMR of compound **8**$^{-}$

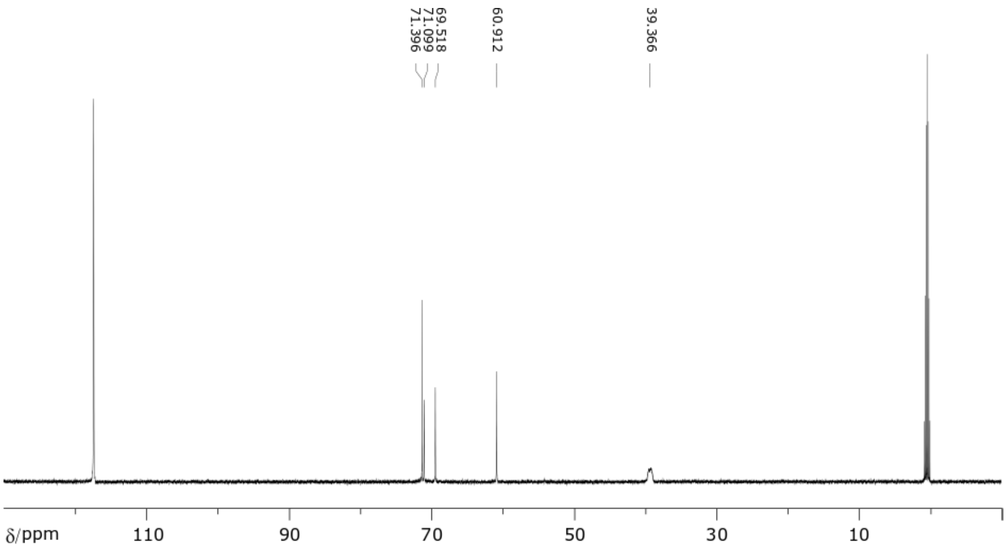

Fig S9c. ^{13}C NMR of compound **8$^-$**

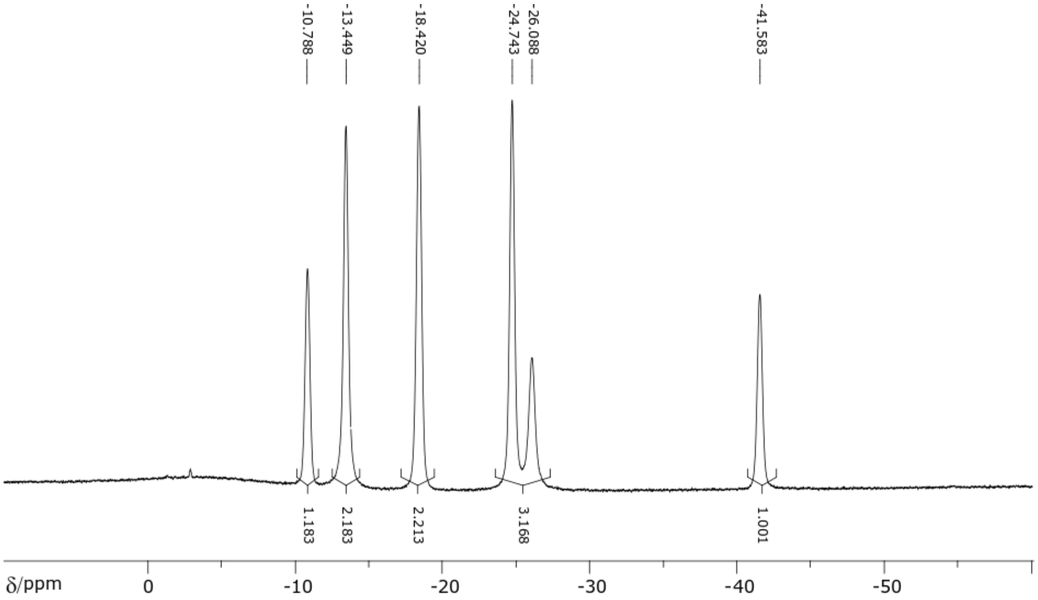

Fig S10a. ^{11}B NMR of compound **9$^-$**

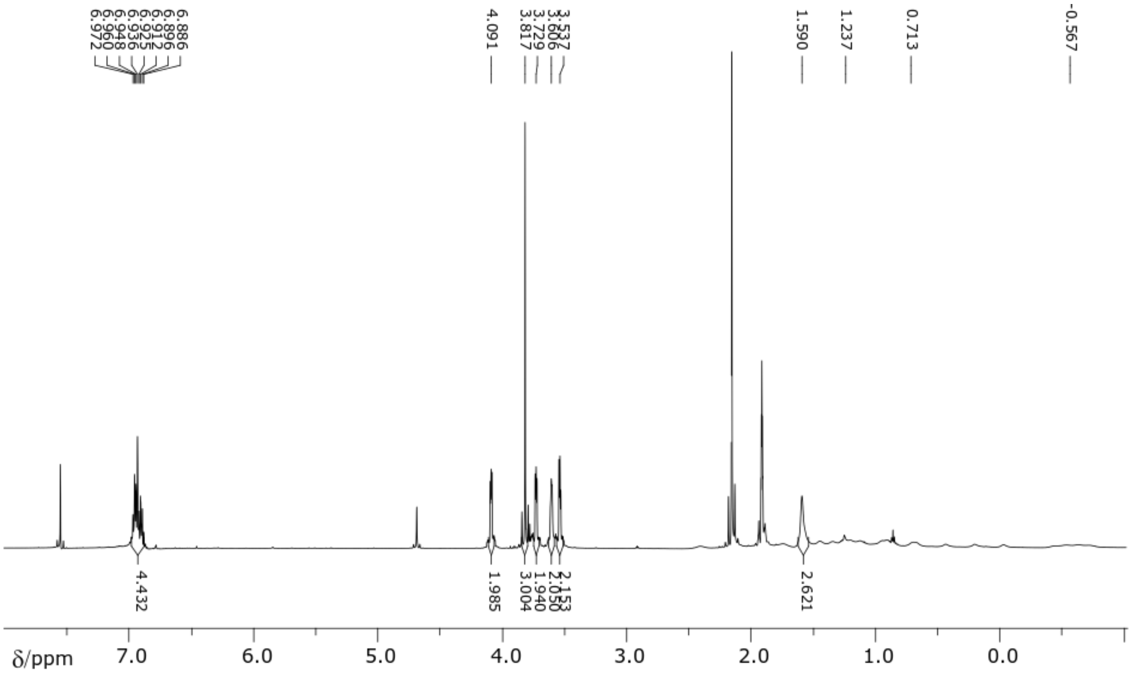

Fig S10b. ^{1}H NMR of compound **9**$^{-}$

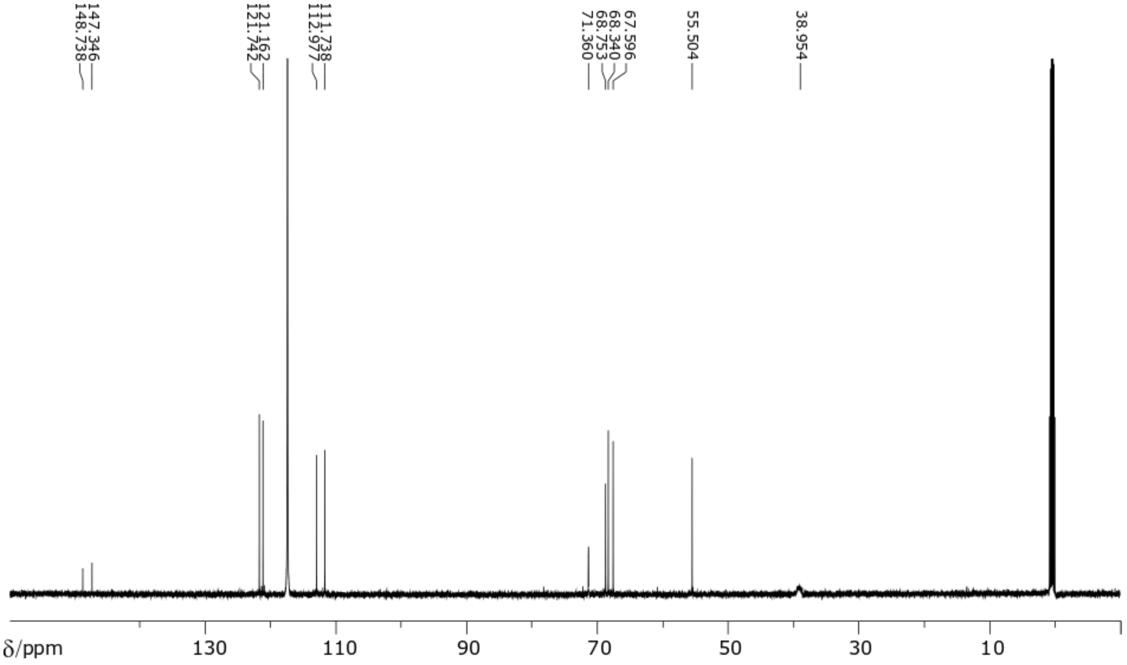

Fig S10c. ^{13}C NMR of compound **9**$^{-}$

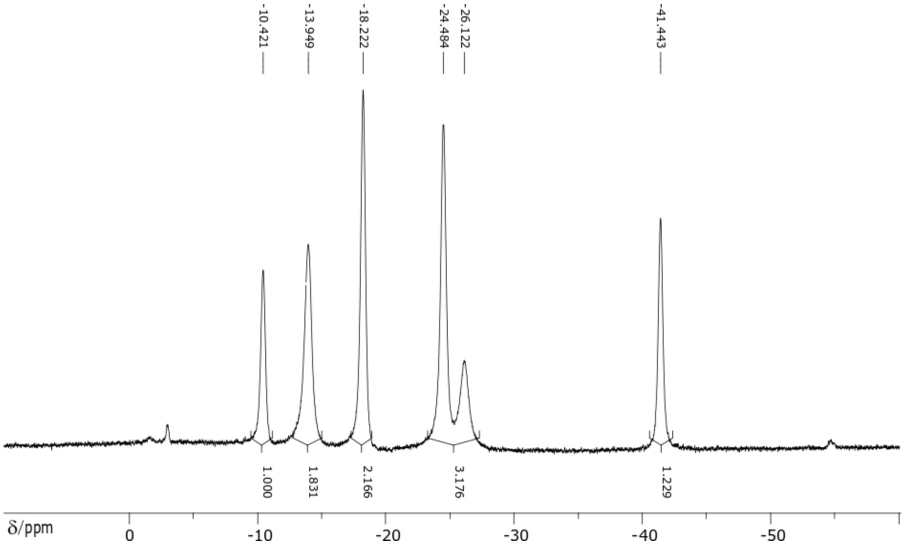

Fig S11a. ^{11}B NMR of compound $\mathbf{10^{2-}}$

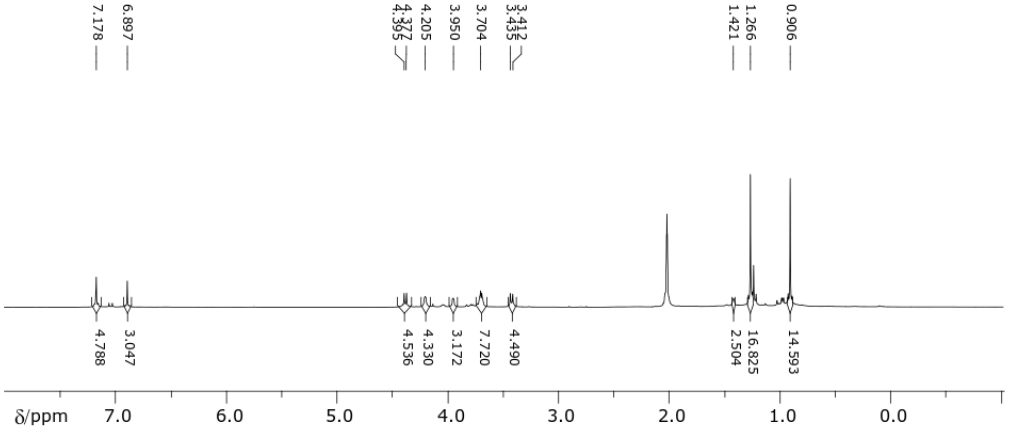

Fig S11b. ^{1}H NMR of compound $\mathbf{10^{2-}}$

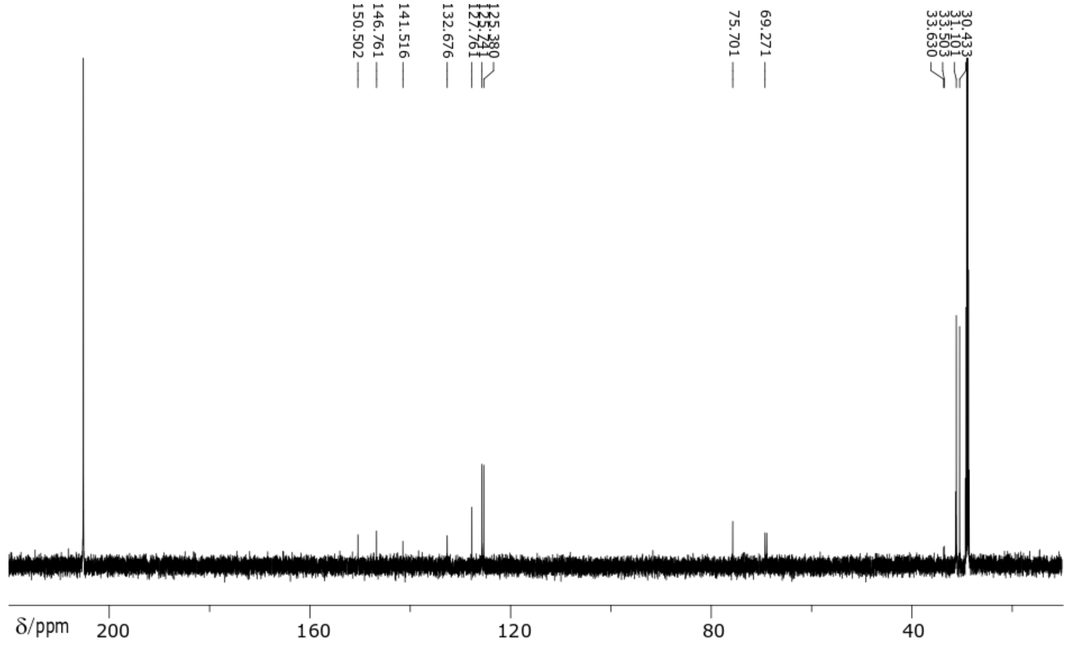

Fig S11c. ^{13}C NMR of compound **10^{2-}**

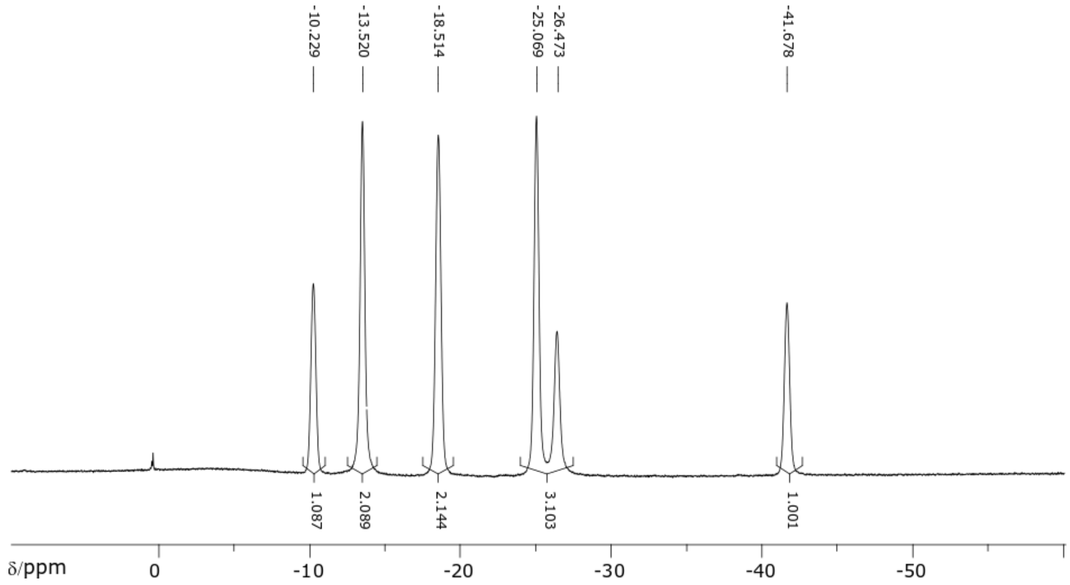

Fig S12a. ^{11}B NMR of compound **11^{-}**

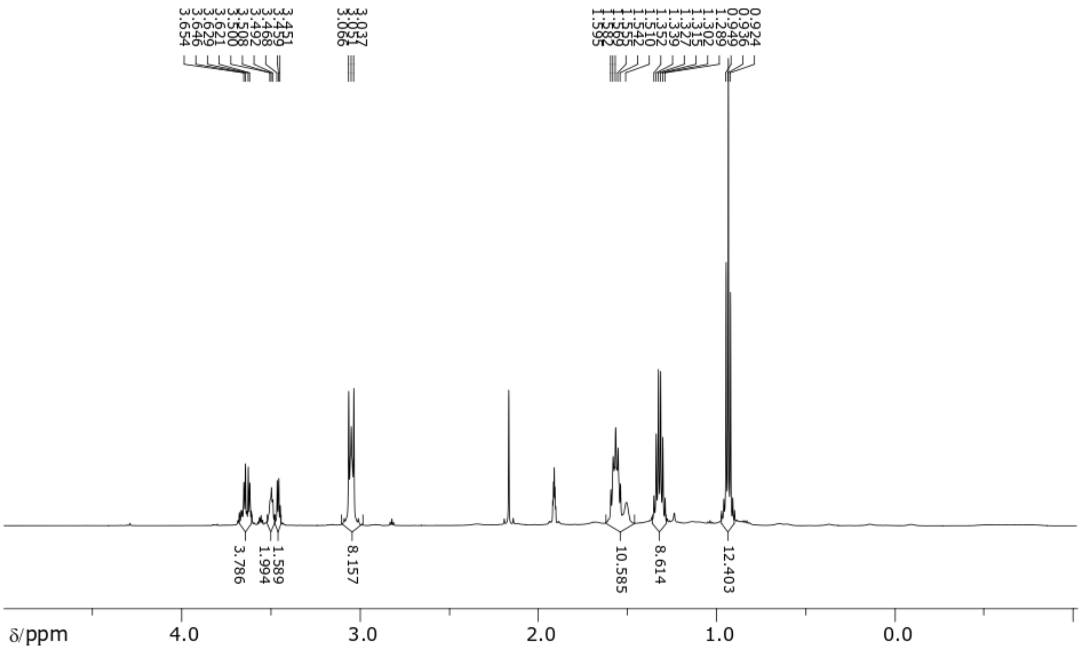

Fig S12b. ^{1}H NMR of compound **11**$^{-}$

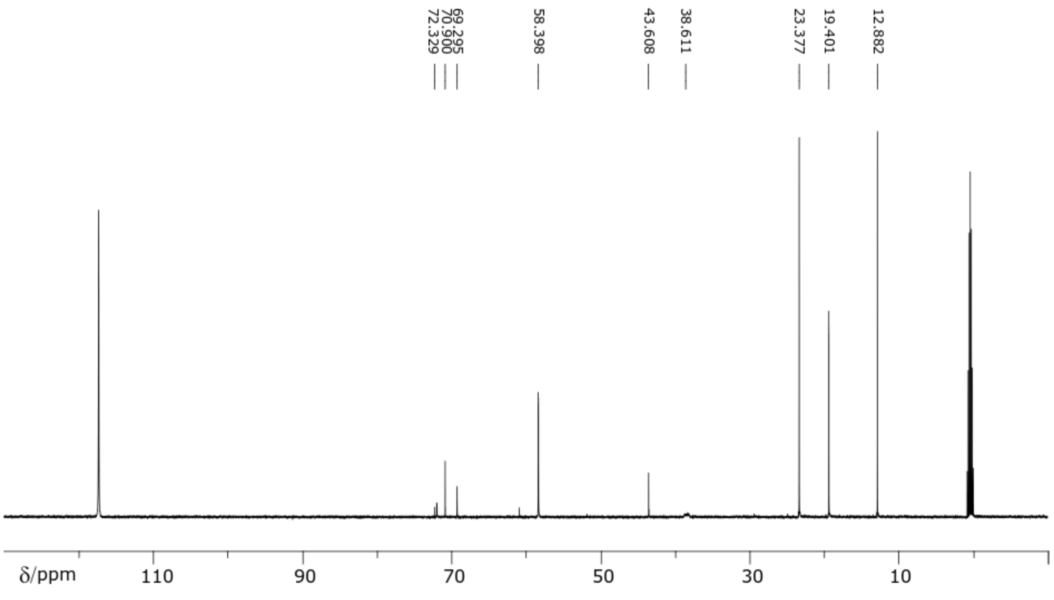

Fig S12c. ^{13}C NMR of compound **11**$^{-}$

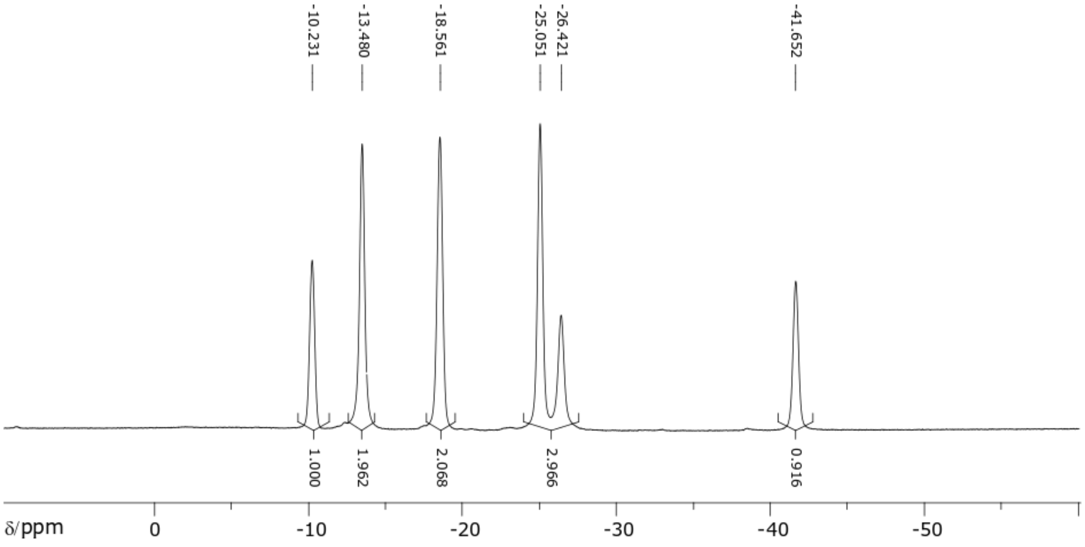

Fig S13a. ^{11}B NMR of compound **12^{-}**

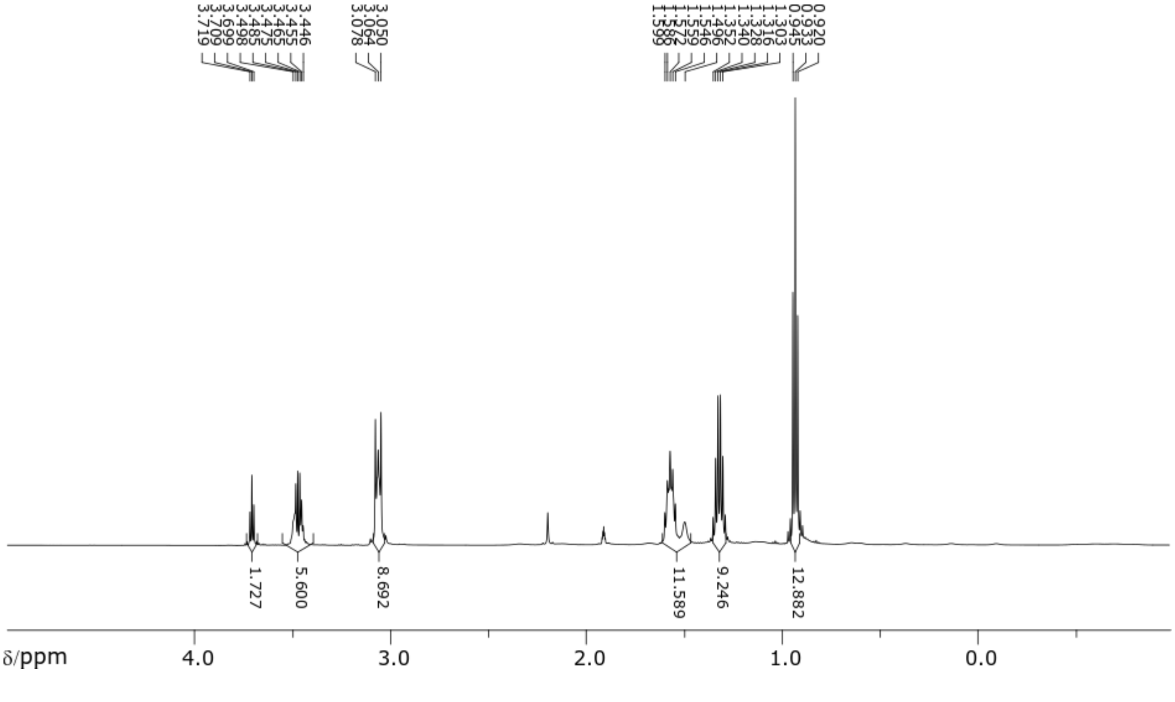

Fig S13b. ^{1}H NMR of compound **12^{-}**

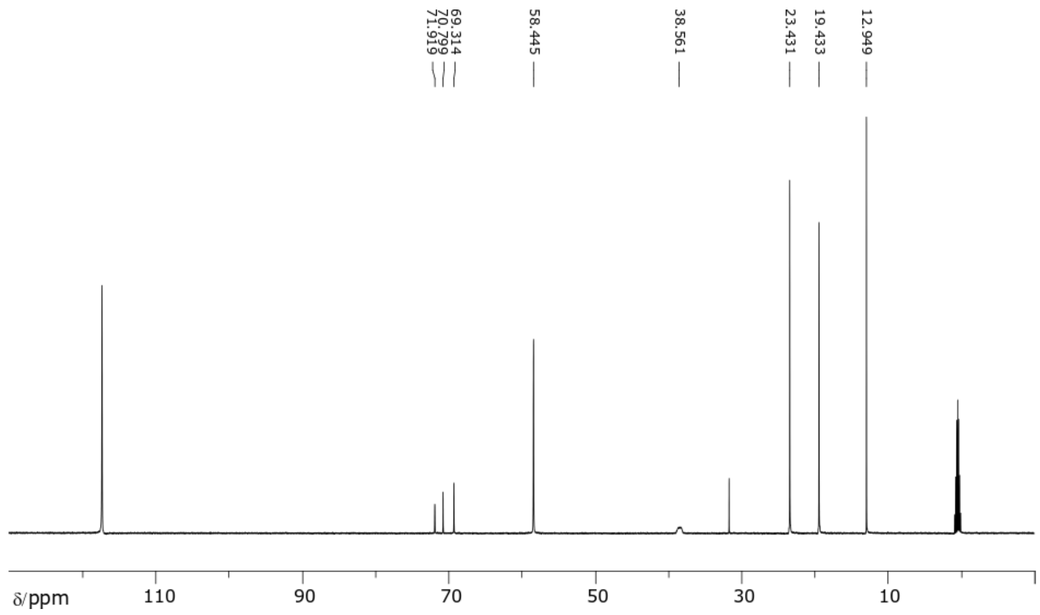

Fig S13c. ^{13}C NMR of compound **12**$^{-}$

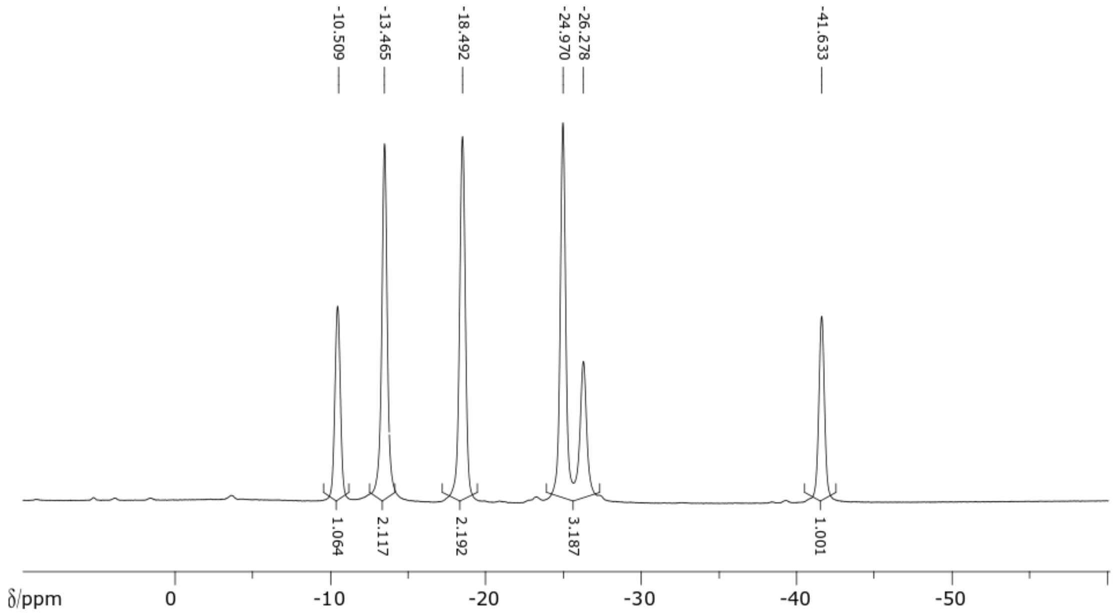

Fig S14a. ^{11}B NMR of compound **13**$^{-}$

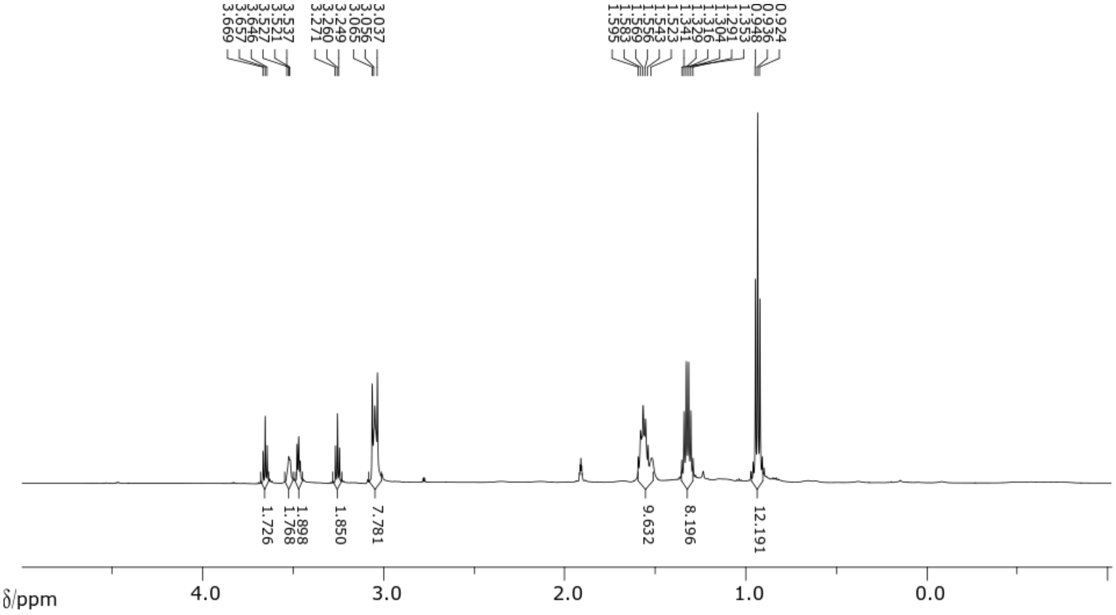

Fig S14b. ^{1}H NMR of compound **13**$^{-}$

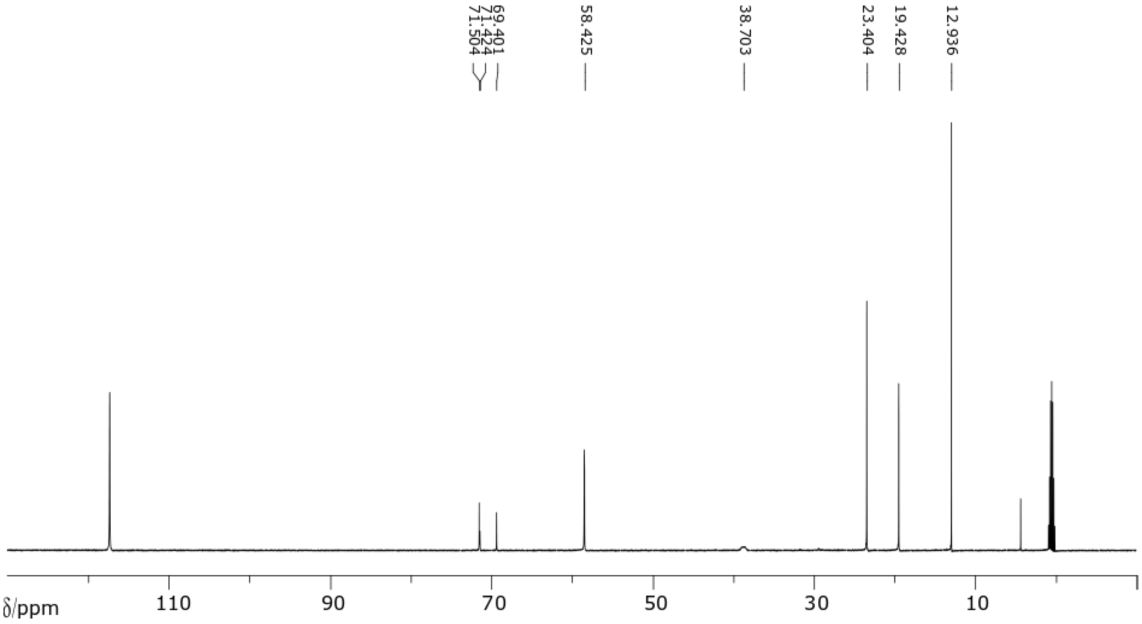

Fig S14c. ^{13}C NMR of compound **13**$^{-}$

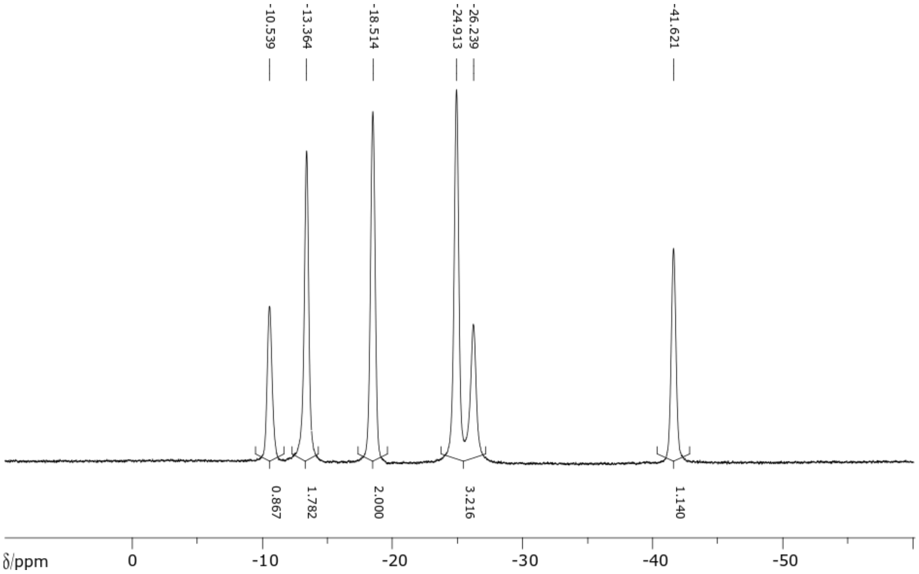

Fig S15a. ^{11}B NMR of compound **14^{-}**

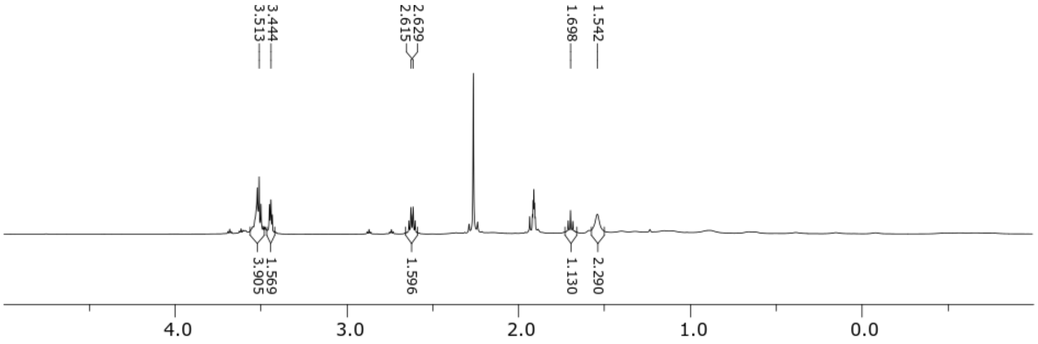

Fig S15b. ^{1}H NMR of compound **14^{-}**

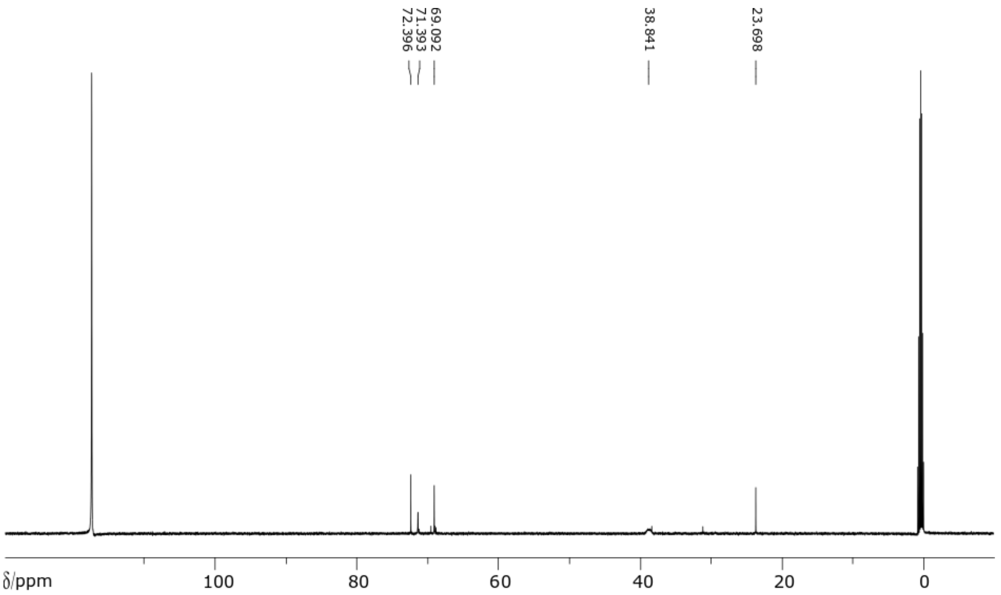

Fig S15c. ^{13}C NMR of compound **14^{-}**

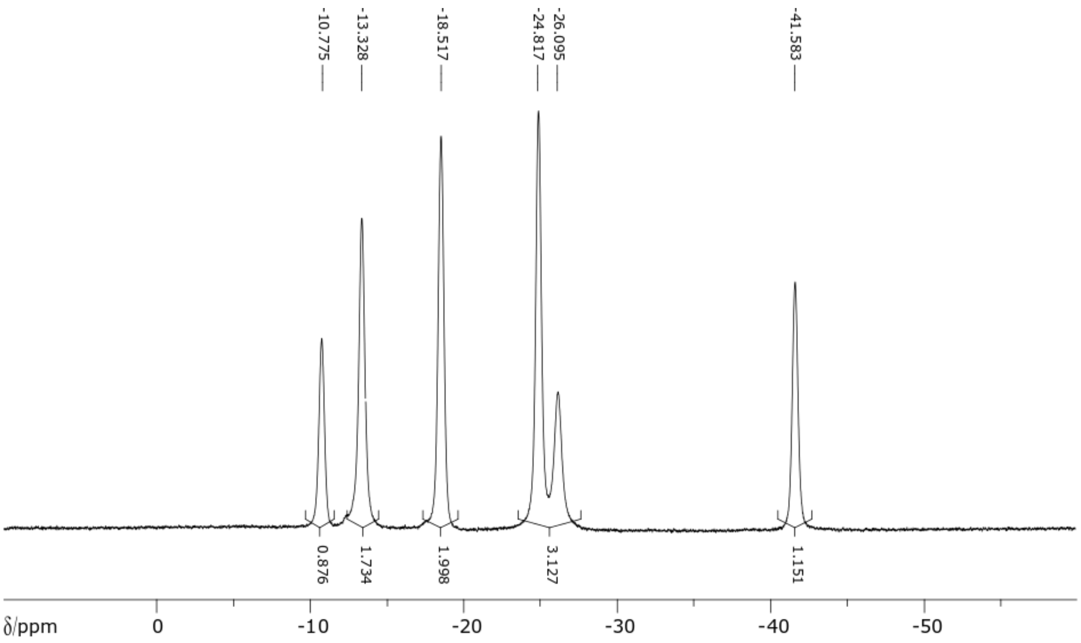

Fig S16a. ^{11}B NMR of compound **15^{2-}**

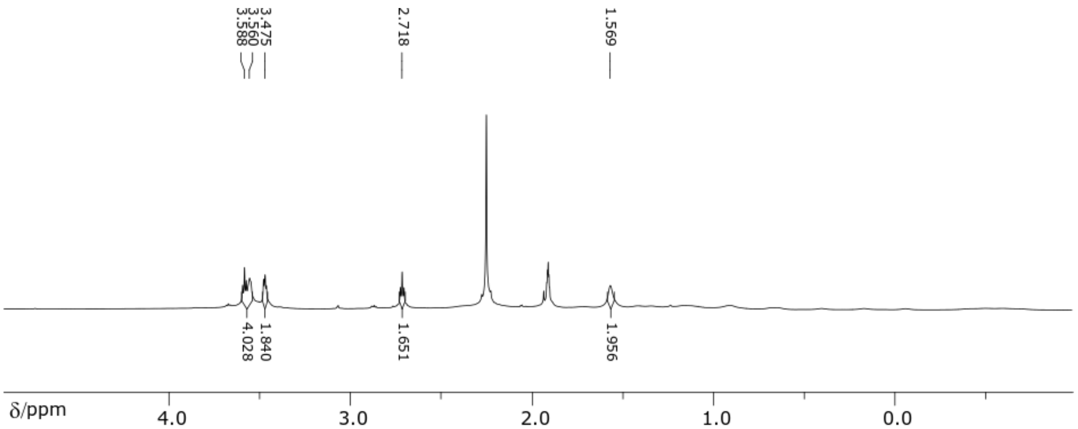

Fig S16b. ^{1}H NMR of compound **15^{2-}**

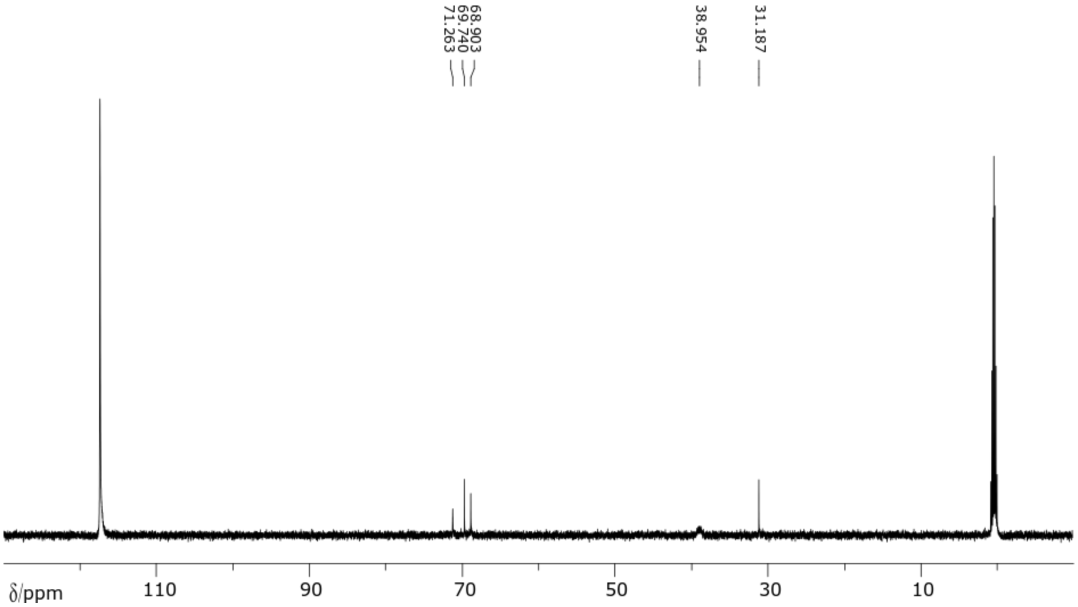

Fig S16c. ^{13}C NMR of compound **15^{2-}**

III. Mass Spectra

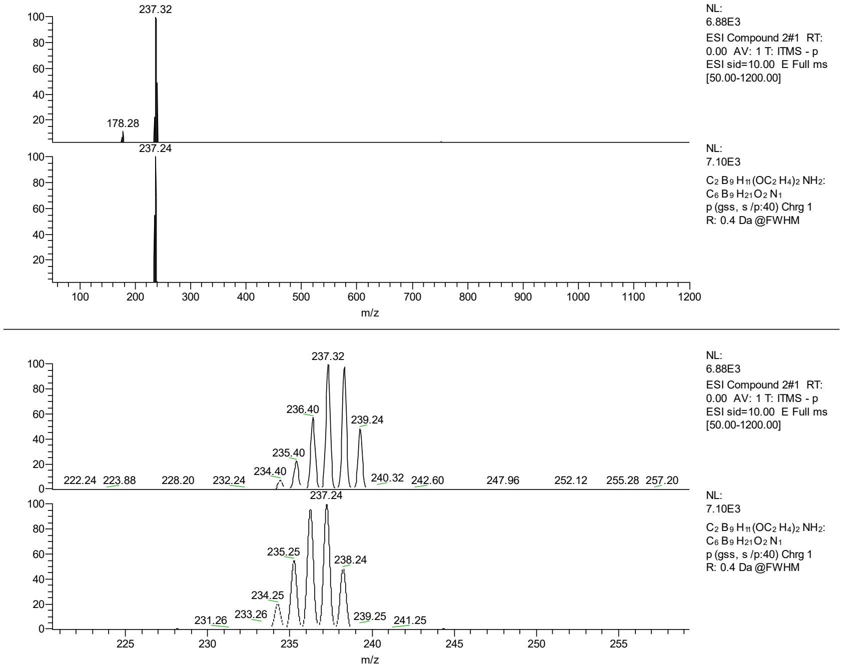

Fig S17. MS spectra of compound **2**

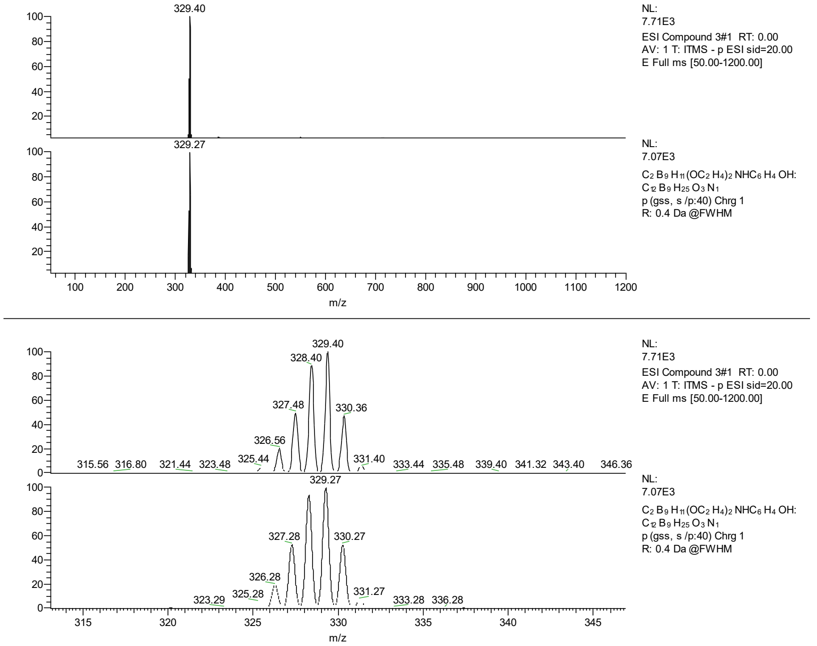

Fig S18. MS spectra of compound **3**

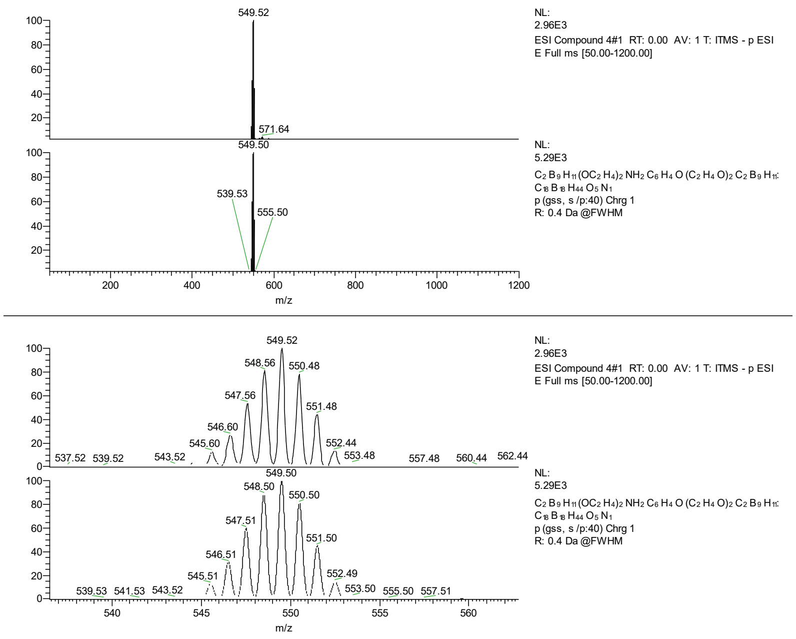

Fig S19. MS spectra of compound **4**$^{-}$

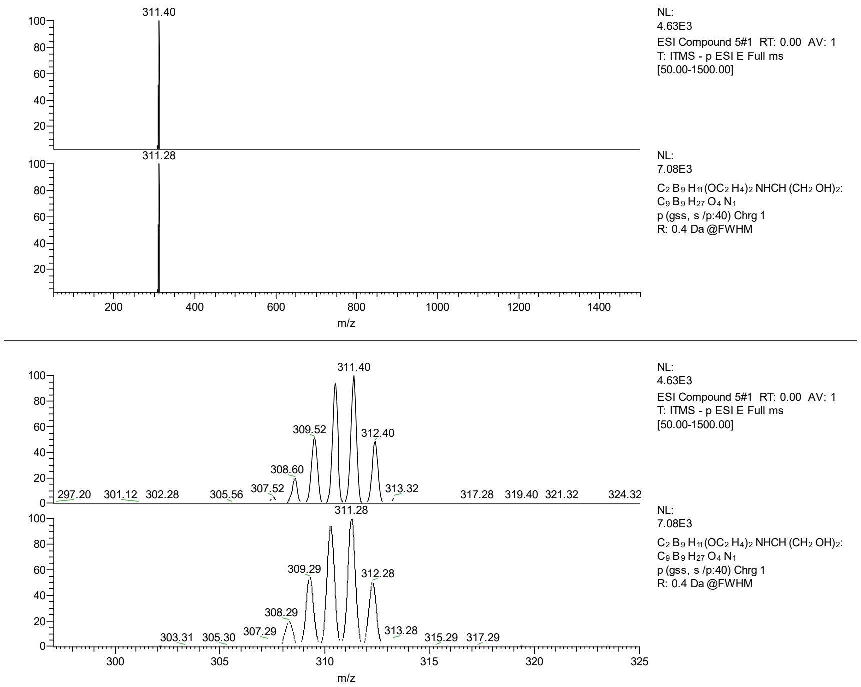

Fig S20. MS spectra of compound **5**

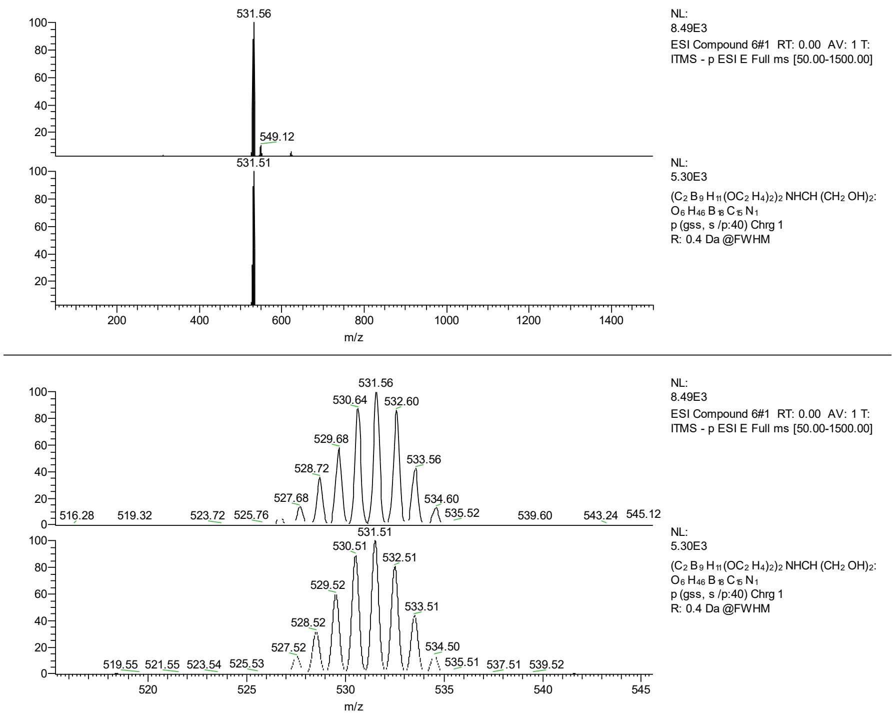

Fig S21. MS spectra of compound **6**$^{-}$

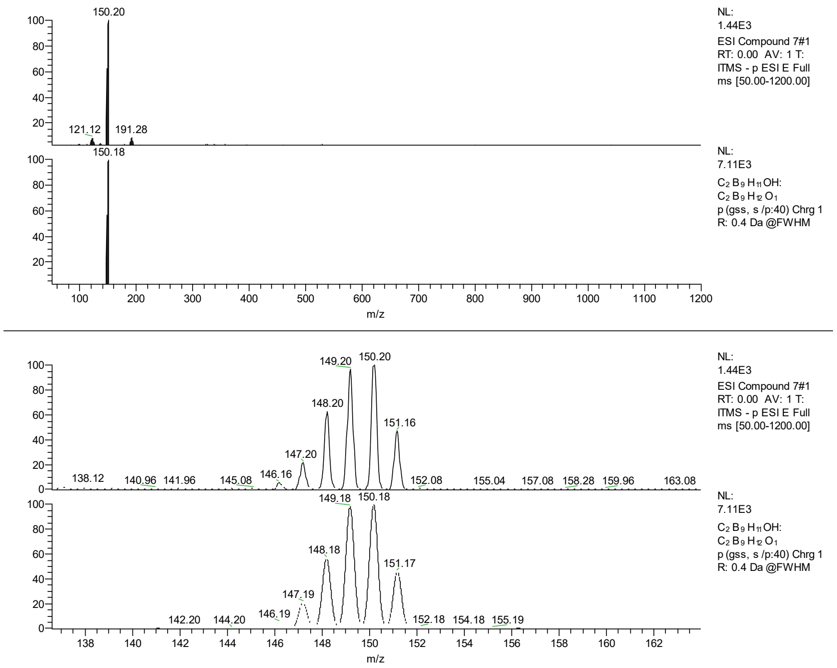

Fig S22. MS spectra of compound **7**$^{-}$

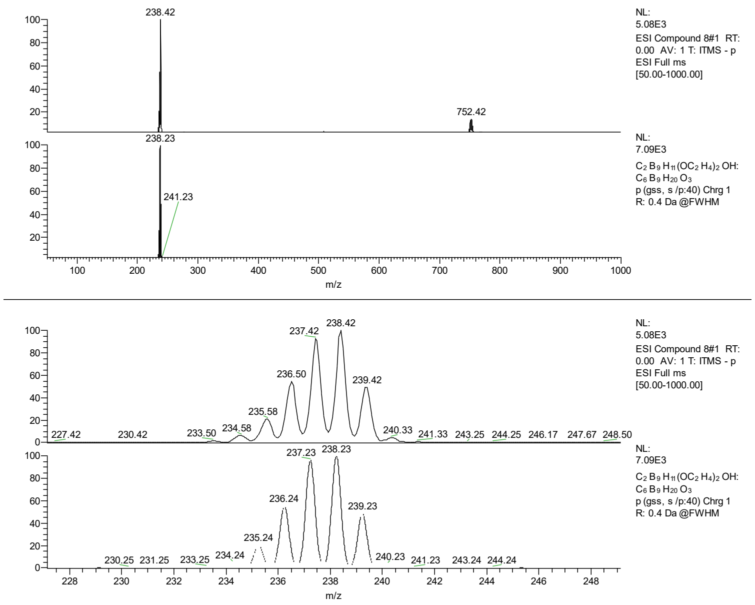

Fig S23. MS spectra of compound **8**⁻

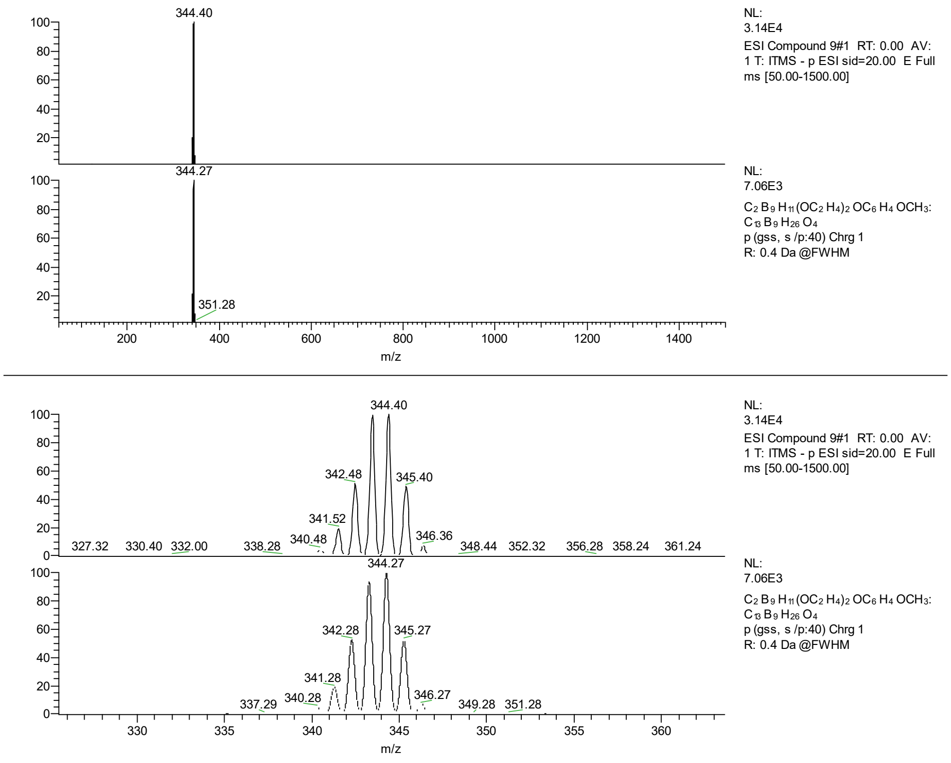

Fig S24. MS spectra of compound **9**⁻

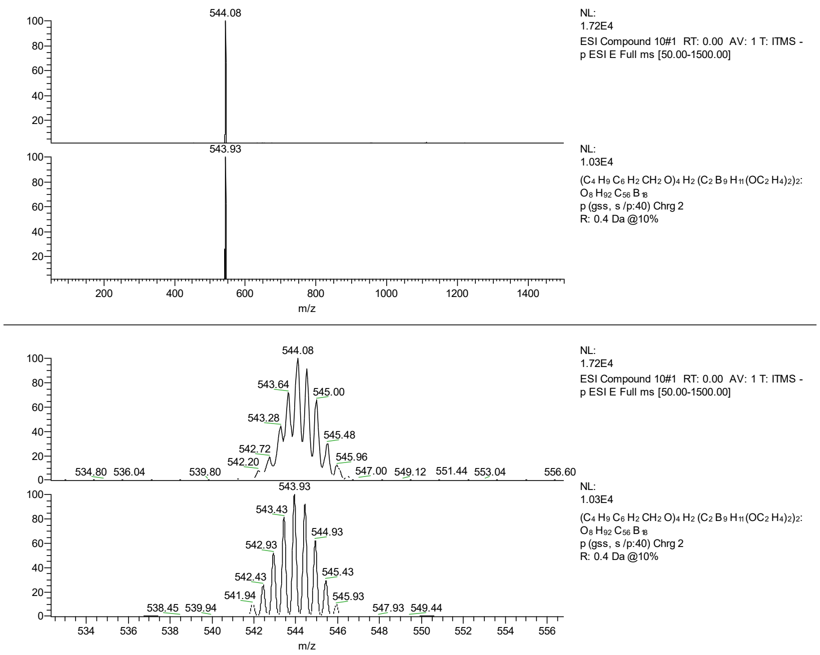

Fig S25. MS spectra of compound $\mathbf{10}^{2-}$

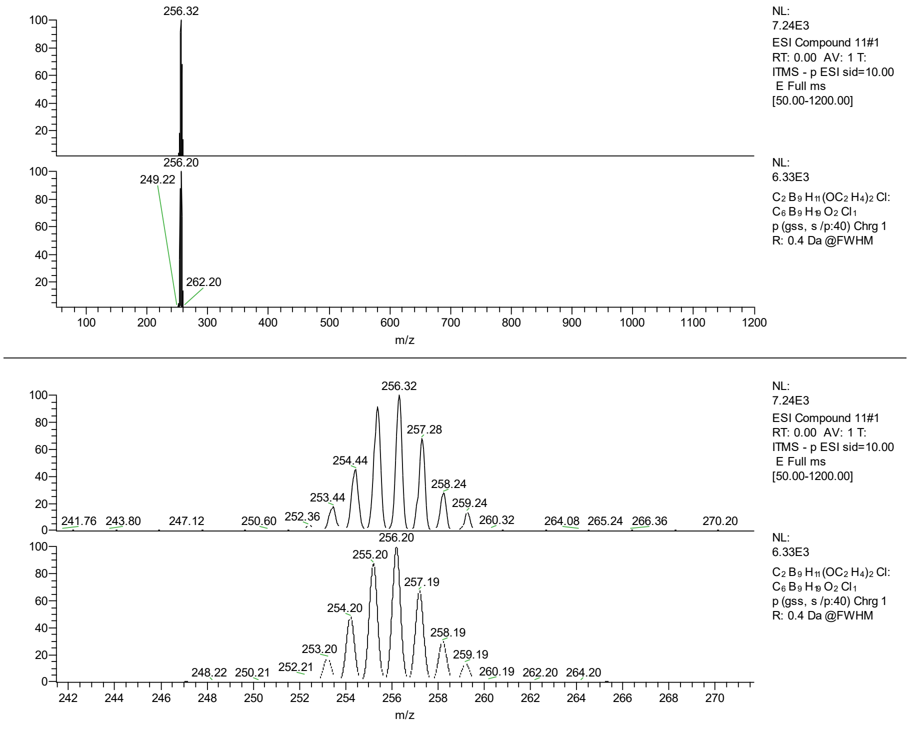

Fig S26. MS spectra of compound $\mathbf{11}^{-}$

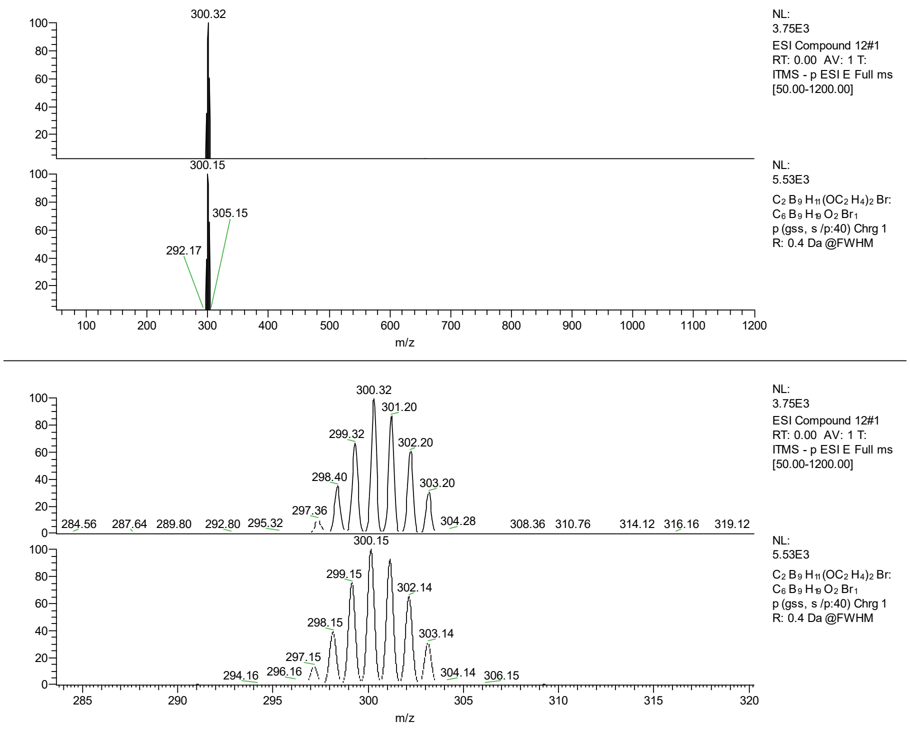

Fig S27. MS spectra of compound **12**$^-$

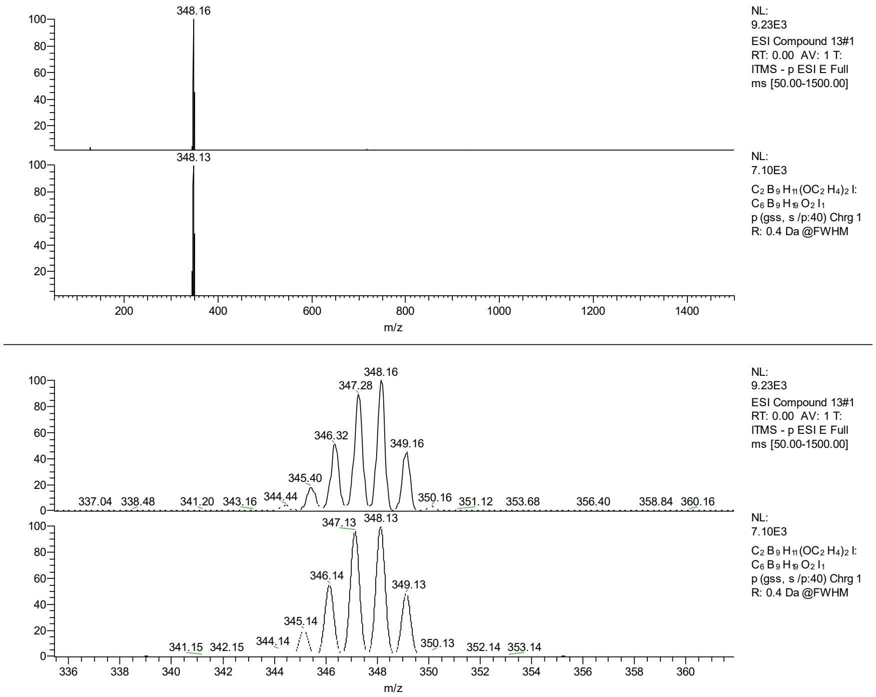

Fig S28. MS spectra of compound **13**$^-$

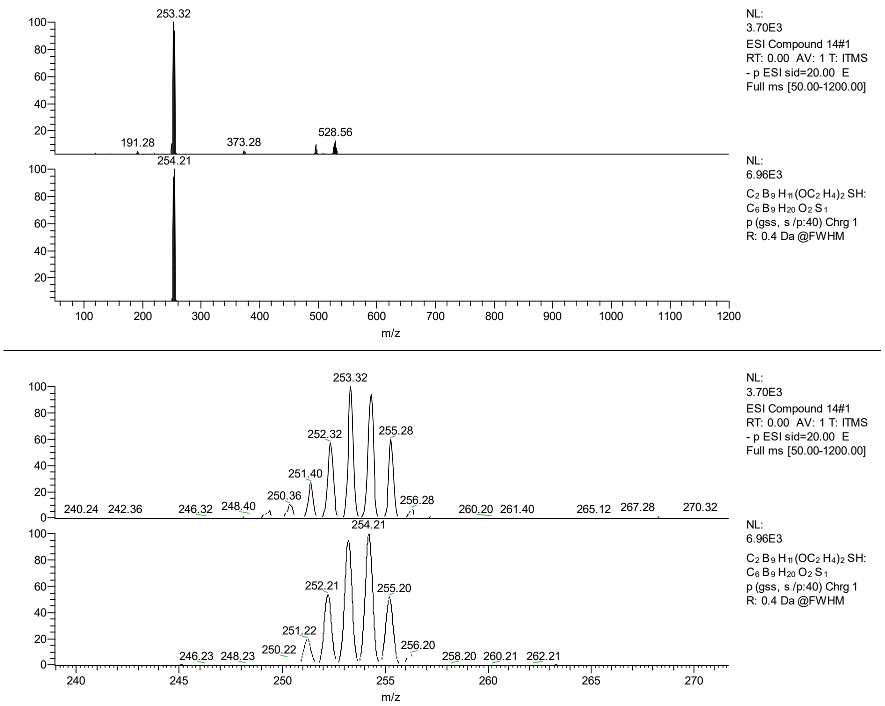

Fig S29. MS spectra of compound **14**$^{-}$

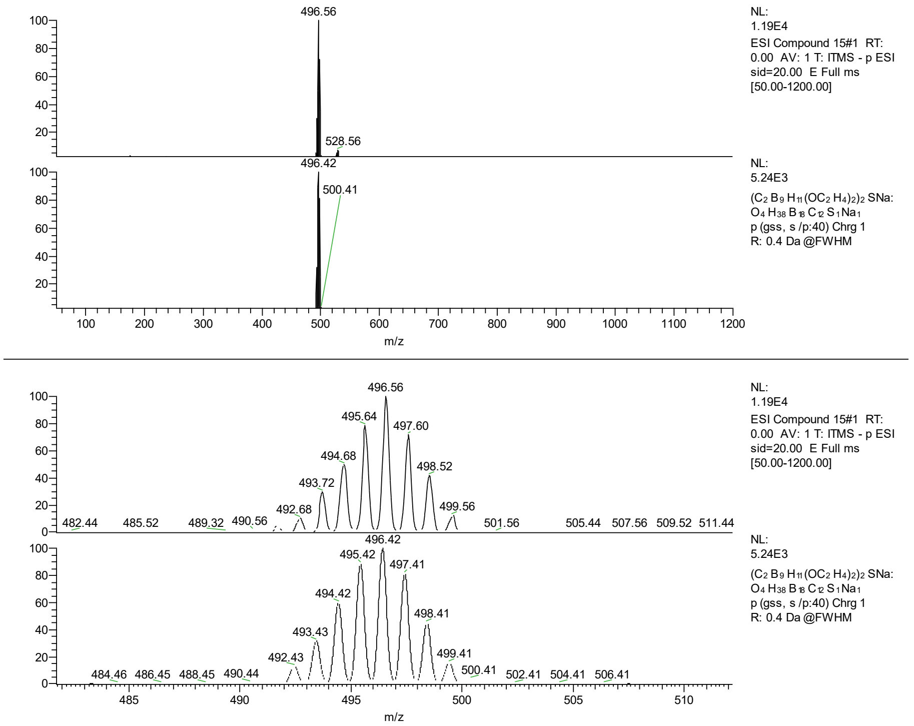

Fig S30. MS spectra of compound **15**$^{2-}$
